# Enhanced Hydrazine Electrooxidation through Benzofuran Derivatives Containing α,β-Unsaturated Dicyano Groups: Synthesis, Electrocatalytic Performance, and Insights from DFT and Topological Analysis

**DOI:** 10.1021/acsomega.5c02472

**Published:** 2025-06-20

**Authors:** Bassam A. Najri, Katia Mohand Saidi, Sefika Kaya, Arif Kivrak, Hilal Kivrak

**Affiliations:** † Department of Chemistry, Science Faculty, 53004 Eskisehir Osmangazi University, Eskisehir 26040, Turkey; ‡ Department of Chemical Engineering, Faculty of Engineering and Architectural Sciences, 53004 Eskisehir Osmangazi University, Eskisehir 26040, Turkey

## Abstract

This study presents an efficient synthetic strategy for the preparation of benzofuran derivatives (**4A-4L**) featuring an α,β-unsaturated dicyano moiety and evaluates their electrocatalytic performance in hydrazine electrooxidation. The synthesis proceeds through a four-step sequence under mild conditions, combining Sonogashira coupling, iodocyclization, Suzuki-Miyaura coupling, and Knoevenagel condensation, resulting in high-yield products. The structures of the synthesized compounds were characterized using ^1^H NMR, ^13^C NMR, FT-IR, and mass spectrometry. Their electrochemical properties were investigated as potential anode catalysts for hydrazine fuel cells through cyclic voltammetry (CV), chronoamperometry (CA), and electrochemical impedance spectroscopy (EIS). Among the screened catalysts, compound (**4A**) demonstrated the highest electrocatalytic activity, achieving a current density of 38.85 mA/cm^2^, establishing it as an effective candidate for hydrazine fuel cells. Additionally, computational studies using density functional theory (DFT) calculations and topology analysis provided further insights into the catalytic performance of compound (**4A**), reinforcing its potential for use in hydrazine fuel cells.

## Graphical abstract

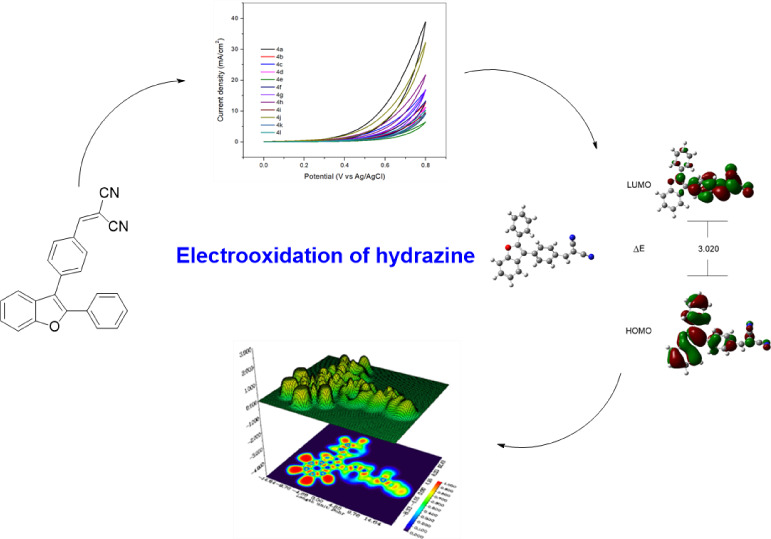

## 1 Introduction

Energy is very critical and important topics all over the worlds. The growing global energy demand, added to the excessive reliance on fossil fuels, is poised to lead to an energy crisis within the next decades.^1–5^ Energy is crucial for essential activities like transportation, communication, production, consumption, and everyday life.^6–9^ However, these resources are finite and will eventually be depleted, making long-term reliance on them unsustainable.^10,11^ Fossil fuels are finite, contribute to global warming, cause environmental harm, and incur high economic costs, underscoring the urgent need for renewable energy sources to ensure energy security and sustainability.^12–16^ Among the solutions, fuel cells have found widespread applications across various industries. Fuel cells utilize chemical energy to produce electrical energy through electrooxidation reactions which are a cleaner and more environmental-friendly solution.^17–19^ Electrooxidation or anodic oxidation or electrochemical oxidation is an electrochemical reaction that arises in an electrochemical system to enable effective energy conversion of fuel to electricity with reduced greenhouse gas emissions.^20–26^ By minimizing reliance on nonrenewable energy resources, fuel cells contribute to the establishment of a more supportable energy situation for industry and society. Besides fossil fuels, other sources of energy like nuclear power^27–29^ and biomass^30^ are also included in the world energy. However, they are also faced with the same challenges related to environmental considerations and sustainable development in the long term.^31,32^ Hydrazine (N_2_H_4_) is a typical organic compound with many uses. It has been widely utilized as a rocket propellant and explosive; however, it also acts a crucial role as a fuel in fuel cells and has significant applications in filmmaking and photography.^33–35^ Hydrazine fuel cell is utilizing as the fuel and oxygen as oxidizer, promising an alternative to conventional energy generation methods. The electrooxidation of hydrazine is highly efficient in the alkaline medium compared to an acidic or neutral medium.^36,37^ The dominant electrooxidation reactions in an alkaline solution can be stated as follows:

$$\mathrm{Anode},\mathrm{reaction}:\;N_{2}H_{4}\;+\;4{\mathrm{OH}}^{-}\;\to \;N_{2}\;+\;4H_{2}O\;+\;4e^{-}$$

$$\mathrm{Cathode}\;\mathrm{reaction}:\;O_{2}\;+\;2H_{2}O\;+\;4e^{-}\;\to \;4{\mathrm{OH}}^{-}$$

$$\mathrm{Overall}\;\mathrm{reaction}:\;N_{2}H_{4}\;+\;O_{2}\;\to \;N_{2}\;+\;2H_{2}O$$

Hydrazine fuel cells offer several advantages, including high energy density, low cost, zero CO_2_ emissions, and straightforward storage and transportation.^38^ However, they also face several limitations, such as lower power density compared to hydrogen fuel cells,^39^ catalytic instability,^40^ and hydrazine toxicity.^41^ Addressing these challenges is crucial for the advancement of next-generation hydrazine fuel cells. A key area of optimization lies in the development of stable and efficient electrooxidation catalysts. Platinum,^42,43^ silver,^44^ cobalt,^45^ nickel,^46^ iron,^47^ and copper^48^ have been widely studied as catalysts for hydrazine electrooxidation. Despite their potential, metal catalysts present several drawbacks, including high costs, limited activity, low selectivity, and susceptibility to poisoning. Due to these limitations, there is a growing demand for alternative materials that can offer enhanced performance. In response, researchers are increasingly exploring organic-based anode catalysts as viable alternatives to metal-based systems. These innovative materials hold the potential to improve the electrocatalytic activity, stability, and selectivity of hydrazine fuel cells, ultimately making them a more sustainable and efficient energy solution. For example, benzofurans, oxygen-containing heterocycles with widespread medicinal and material applications,^49^ are gaining attention as potential catalysts for electrooxidation reactions. Modifying the aromatic ring with the oxygen heterocycle can alter the electron density and modify the compound’s physical, chemical, and biological properties.^50^ As a result, the synthesis of functionalized benzofuran derivatives has become an area of significant interest among researchers. Several synthetic methods of benzofurans are stated in the literature. One of the major strategies is founded on the palladium/copper (Pd/Cu)-catalyzed Sonogashira cross-coupling of 2-iodoanisoles and alkynes, followed by electrophilic cyclization with iodine.^51^ This method allows the formation of 2,3-disubstituted benzofurans with various substituents on the benzene and furan rings. Moreover, the resulting iodobenzofurans can be further modified by palladium-catalyzed Suzuki-Miyaura cross-coupling with different boronic acids or esters. α,β-Unsaturated dicyano compounds have recently fascinated large attention due to their application in ultrafast and ultrasensitive molecular electronic devices and ultrahigh-density data storage.^52^ The synthesis of α,β-unsaturated dicyano compounds or associated compounds via condensation reaction of aldehydes or ketones with active methylene compounds (e.g., malononitrile) catalyzed by weak bases such as amines (e.g., ammonia, urea, pyridine), aluminum oxide (Al_2_O_3_), etc. commonly referred to as the Knoevenagel condensation.^53^

Herein, we report an efficient four-step synthetic strategy for benzofuran derivatives (**4A-4L**), whose chemical structures are shown in Figure 1, featuring an α,β-unsaturated dicyano moiety and evaluate their electrocatalytic performance in hydrazine electrooxidation. The synthesized compounds were characterized using spectroscopic techniques and assessed as anode catalysts through CV, CA, and EIS. Among them, compound (**4A**) exhibited the highest catalytic activity, demonstrating significant potential for hydrazine fuel cells. Additionally, DFT calculations and topology analysis provided insights into its electronic structure, further supporting its promising application in energy conversion.

**Figure 1:**
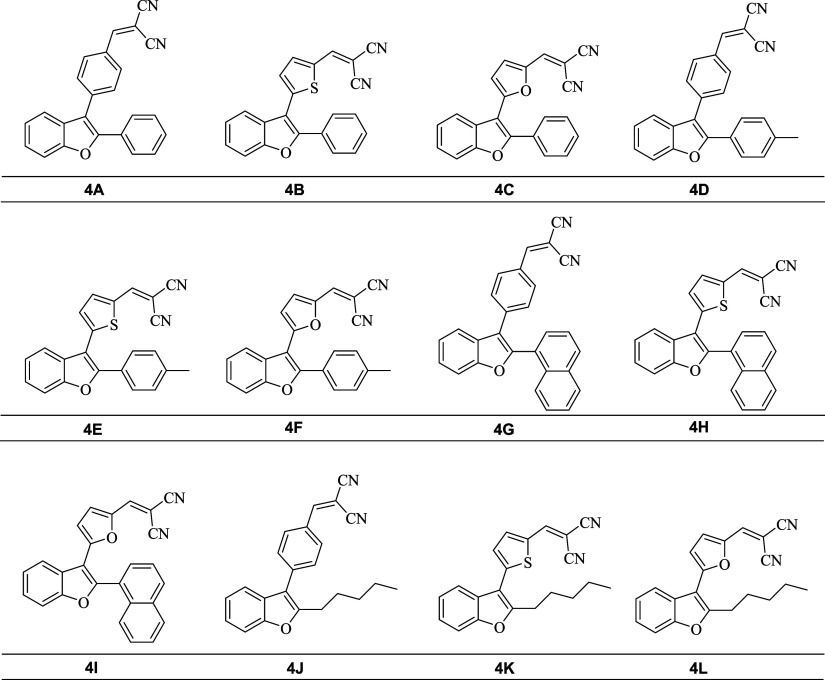
Chemical structures of compounds (**4A-4L**).

## 2 Experimental Section

### 2.1 Synthesis of 2-((5-(2-Aryl/alkyl-benzofuran-3-yl)­aryl-2 yl)­methylene)­malononitrile (4A-4L)

The synthesis of the target compounds (**4A-4L**) was carried out in a multistep process involving Sonogashira coupling, iodocyclization, Suzuki-Miyaura coupling, and Knoevenagel condensation reactions.

#### 2.1.1 Synthesis of 1-Methoxy-2-(aryl/alkyl-ethynyl)­benzene (1A-1D) via Sonogashira Coupling

Following the procedure outlined by Najri et al. (2024),^54^ (1 equiv) of 2-iodoanisole in tetrahydrofuran (THF) (10 mL) was treated with the corresponding alkyne (1.2 equiv), triethylamine (Et_3_N) (15 mL), palladium­(II) bis­(triphenylphosphine) dichloride (PdCl_2_(PPh_3_)_2_) (2.5 mol %), and copper­(I) iodide (CuI) (1 mol %). The reaction mixture was stirred under an argon atmosphere at room temperature for 16 h. Upon completion, the reaction mixture was diluted with ethyl acetate (3 × 25 mL) and brine. The organic layer was dried over anhydrous magnesium sulfate (MgSO_4_), and the crude product was purified by silica gel column chromatography using hexane as the eluent to afford compounds (1A-1D).

#### 2.1.2 Synthesis of 3-Iodo-2-aryl-benzofuran (2A-2D) via Iodocyclization

The iodocyclization reaction was performed according to the procedure described by Najri et al. (2024).^54^ In the preparation of 3-iodo-2-aryl-benzofuran (2A-2C), a solution of compounds (1A-1C) (1 equiv) in dichloromethane (DCM) (25 mL) was exposed to iodine (I_2_) (1 equiv) and stirred at room temperature for 4 h under an argon-rich atmosphere. The reaction was quenched by a saturated solution of sodium thiosulfate (Na_2_S_2_O_3_) and then extracted with chloroform (3 × 25 mL). The organic phase was dried over anhydrous MgSO_4_, and the crude product was purified through silica gel column chromatography using a combination of hexane and ethyl acetate as the eluent. For the synthesis of 3-iodo-2-alkyl-benzofuran (2D), compound (1D) (1 equiv) in 1,2-dichloroethane (DCE) (25 mL) was mixed with sodium bicarbonate (NaHCO_3_) (3 equiv) and I_2_ (3 equiv) and was refluxed at 70 °C for an overnight period under an argon atmosphere. The reaction mixture was subsequently quenched through a saturated sodium Na_2_S_2_O_3_ and extracted with chloroform (3 × 25 mL). The combined organic layers were dried over anhydrous MgSO_4_, and the collected product was purified by silica gel column chromatography with hexane/ethyl acetate as the eluent.

#### 2.1.3 Synthesis of 5-(2-Aryl/alkyl-benzofuran-3-yl)­aryl-2-carbaldehyde (3A-3L) via Suzuki-Miyaura Coupling

The reaction was performed according to the procedure described by Najri et al. (2025),^55^ where the compounds (2A-2D) (1 equiv) were dissolved in THF (5 mL) in a microwave vial, followed by the addition of palladium­(II) acetate (Pd­(OAc)_4_) (10 mol %), triphenylphosphine (PPh_3_) (20 mol %), boronic acid (1.5 equiv), and sodium hydroxide (NaOH) (3 equiv) under an argon atmosphere. After the addition of water (1 mL), the reaction mixture was heated in a microwave reactor at 70 °C for 45 min. The reaction mixture was then cooled to room temperature, diluted with ethyl acetate (3 × 25 mL), and washed with brine. The organic layers were combined, dried over MgSO_4_, and filtered. The crude product was purified by silica gel column chromatography using hexane/ethyl acetate as the eluent to give the corresponding aldehyde derivatives (3A-3L).

#### 2.1.4 Synthesis of 2-((5-(2-Aryl/alkyl-benzofuran-3-yl)­aryl-2-yl)­methylene)­malononitrile (4A-4L) via Knoevenagel Condensation

A mixture of the aldehyde derivatives (3A-3L) (1 equiv), malononitrile (4 equiv), and aluminum oxide (Al_2_O_3_) (1 equiv) in toluene (5 mL) was stirred at room temperature overnight under an argon atmosphere. After completion, the reaction mixture was filtered using chloroform, and the filtrate was purified by silica gel column chromatography using hexane/ethyl acetate as the eluent.

### 2.2 Electrochemical Measurements

Electrochemical measurements were performed using CV, CA, and EIS to evaluate the electrooxidation performance of the organic catalysts (**4A-4L**) in the presence of hydrazine, conducted in 1 M KOH and a mixture of 1 M KOH + 0.5 M N_2_H_4_ solutions. The electrochemical data were collected with a CHI660E potentiostat in a three-electrode system, comprising a glassy carbon electrode (GCE) as the working electrode with a surface area of 0.07065 cm^2^, a platinum wire as the counter electrode, and an Ag/AgCl reference electrode. CV measurements were conducted over a potential range of 0.0 to 0.8 V at a scan rate of 50 mV/s. To assess the catalyst stability, CA measurements were carried out at fixed potential values for a 1000-s duration, while EIS measurements were performed at potential values of 0.0, 0.2, 0.4, 0.6, and 0.8 V to evaluate the resistance of the organic catalysts (**4A-4L**) during hydrazine electrooxidation.

To prepare the catalyst, 3 mg of the organic catalysts (**4A-4L**) were evenly dispersed in 0.5 mL of Nafion solution to form a catalyst ink. A 3 μL aliquot (approximately 0.018 mg of catalyst) of the ink was applied to a GCE, which had been prepolished with alumina. The electrode was then dried at room temperature to remove any residual solvent.

### 2.3 Computational Studies

#### 2.3.1 DFT Calculations

The computational calculations for the catalyst (**4A**) were performed using Gaussian 09W software,^56^ employing the B3LYP exchange-correlation functional with LYP correction and the 6-31G­(d,p) basis set.^57^ Solvent effect was included using the default model (IEF-PCM: Integral-Equation-Formalism Polarizable Continuum Model)^58,59^ with toluene as a solvent. The optimized molecular structure, along with, the highest occupied molecular orbital (HOMO) and lowest unoccupied molecular orbital (LUMO), were visualized by GaussView 6.0 (license: IA32W-G09RevD.01 24-Apr-2013).^60^ A number of electronic properties were calculated, such as the HOMO and LUMO energies, the energy gap (Δ*E*_gap_), chemical potential (μ), hardness (η), and global electrophilicity index (ω). These parameters are important for assessing the electronic structure and reactivity of the catalyst. Particularly, the Δ*E*_gap_ is an indicator of molecular stability, where a larger gap is indicative of greater stability and a smaller gap of greater reactivity.^61^ Electronic properties were determined from the following equations:

$$\mu \;=\;[E_{\mathrm{HOMO}}\;+\;E_{\mathrm{LUMO}}]/2$$

$$\eta \;=\;[E_{\mathrm{LUMO}}\;-\;E_{\mathrm{HOMO}}]/2$$

S =  1/ η

Furthermore, the molecular electrostatic potential (MEP) plot was examined to gain deeper insight into the molecular reactivity and charge distribution within the system.

#### 2.3.2 Topology Analysis

The chk file was converted to an fch file using Gaussian 09W, after which Multiwfn version 3.8 was employed to analyze the electron localization function (ELF), localized orbital locator (LOL), average local ionization energy (ALIE), and interaction region indicator (IRI) of the catalyst (**4A**).

## 3 Results and Discussion

### 3.1 Synthesis

The synthesis of novel benzofuran derivatives containing an α,β-unsaturated dicyano moiety (**4A-4L**) was achieved through a four-step reaction sequence, as illustrated in Scheme 1. The process began with the Sonogashira cross-coupling reaction between 2-iodoanisole and alkyne derivatives, forming corresponding alkyne derivatives (1A-1D) in high yields. This step effectively introduced the alkyne functionality, setting the foundation for subsequent cyclization. The iodocyclization of the obtained 1-methoxy-2-(aryl/alkyl-ethynyl)­benzenes (1A-1D) with I_2_ in DCM at room temperature successfully yielded the corresponding 3-iodo-2-aryl/alkyl-benzofuran derivatives (2A-2D). However, for compound 1D, the reaction required reflux at 70 °C and the addition of NaHCO_3_ to achieve complete conversion, likely due to the electronic effects of the alkyl substituent. In the next step, the 2-substituted-3-iodobenzofurans (2A-2D) were subjected to Suzuki-Miyaura cross-coupling with various boronic acids, including 4-formylphenylboronic acid, 5-formylthiophene-2-boronic acid, and 5-formylfuran-2-boronic acid. This reaction effectively furnished the 5-(2-aryl/alkyl-benzofuran-3-yl)­aryl-2-carbaldehyde derivatives (3A-3L) in a good yields, demonstrating the efficiency of the Suzuki reaction in constructing structurally diverse benzofuran frameworks. Finally, the Knoevenagel condensation of aldehyde intermediates (3A-3L) with malononitrile, catalyzed by Al_2_O_3_, resulted in the formation of the target 2-((5-(2-aryl/alkyl-benzofuran-3-yl)­aryl-2-yl)­methylene)­malononitrile derivatives (**4A-4L**). This transformation proceeded smoothly under mild conditions, highlighting the effectiveness of Al_2_O_3_ as a heterogeneous catalyst. The synthetic pathway efficiently yielded the targeted benzofuran derivatives with high selectivity. Their structural integrity was verified using spectroscopic techniques such as NMR, FTIR and mass spectrometry.

**Scheme 1:**
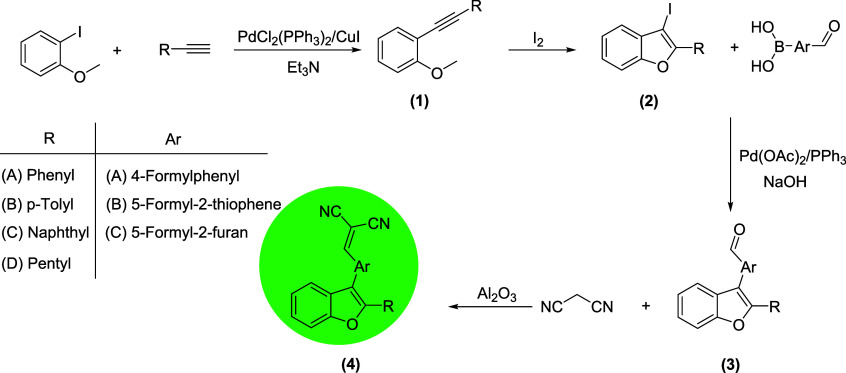
Synthesis of Benzofuran Derivatives (**4A-4L**) Featuring an α,β-Unsaturated Dicyano Moiety

To illustrate the spectral changes associated with the conversion from aldehyde to α,β-unsaturated dicyano compounds, compound (**4A**) was selected as a representative example. The FT-IR spectrum (Figure S55) of (**4A**) displayed characteristic absorption bands at 2226 and 1591 cm^–1^, corresponding to the nitrile (−CN) and C=C (double bond) functional groups, respectively. A significant observation was the absence of the carbonyl (C=O) absorption band at 1705 cm^–1^, along with the absence of aldehydic C–H stretch bands at 2828 and 2736 cm^–1^, confirming the complete transformation of the aldehyde moiety. Further structural validation was provided by ^13^C NMR (Figure S54), where the disappearance of the carbonyl signal at 190.01 ppm indicated the loss of the aldehyde group, while two new peaks at 112.89 and 114.03 ppm were attributed to the two nitrile (−CN) groups. Additionally, ^1^H NMR (Figure S53) confirmed the presence of the α,β-unsaturated dicyano (−HC=C­(CN)_2_) system, as evidenced by a singlet peak at 7.79 ppm, with a corresponding peak at 159.24 ppm in the ^13^C NMR spectrum. Mass spectrometry analysis (Figure S56) further supported the structural assignment of compound (**4A**). The LC-MS/MS spectrum showed a molecular ion peak corresponding to [M + H]^+^ at *m*/*z* 347.11859, which closely matched the calculated value of *m*/*z* 347.11789 for the molecular formula C_24_H_14_N_2_O, confirming the expected molecular composition. These spectroscopic and mass spectrometric findings confirm the successful conversion of the aldehyde precursor into the targeted α,β-unsaturated dicyano derivative, highlighting the effectiveness of the synthetic strategy employed.

### 3.2 Electrochemical Measurements

The electrooxidation activity of hydrazine on the catalysts (**4A-4L**) was evaluated using CV measurements in 1 M KOH and 1 M KOH + 0.5 M N_2_H_4_ solutions at a scan rate of 50 mV/s within a potential range of 0.0–0.8 V. The results, as shown in Figure 2 and Table 1, revealed the absence of oxidation peaks in the forward scan, indicating that electrocatalytic activity was assessed based on the total current density value. Among the tested catalysts, compound (**4A**) exhibited the highest electrocatalytic activity, with a current density of 38.85 mA/cm^2^ in N_2_H_4_ solution, outperforming all other derivatives. Compound (**4A**) contains an α,β-unsaturated dicyano group connected to a phenyl group, with the benzofuran ring also attached to a phenyl group. This simple and planar structure allows for efficient electron transfer, facilitating better adsorption and activation of hydrazine molecules on the electrode surface.^54^ As a result, (**4A**) achieves the highest current density in the series, reflecting its superior catalytic performance. In contrast, compounds such as (4B), (4E), (4H), and (4K), which contain thiophene groups, exhibit lower catalytic performance. The sulfur atom in thiophene donates electron density, reducing the electrophilicity of the molecules and impairing their ability to effectively interact with hydrazine. This leads to decreased catalytic efficiency, as evidenced by lower current densities. In N_2_H_4_ solution, compound (4B) has a current density of 12.60 mA/cm^2^, (4E) shows 6.27 mA/cm^2^, (4H) exhibits 21.40 mA/cm^2^, and (4K) shows 8.78 mA/cm^2^. Similarly, compounds like (4C), (4F), (4I), and (4L), which feature furan groups, also show diminished catalytic activity. Although furan groups provide some electron density, they do not enhance electrophilicity to the same extent as the phenyl group in (4**A**), leading to reduced catalytic performance. The current densities in N_2_H_4_ solution for these compounds are 16.63 mA/cm^2^ for (4C), 13.14 mA/cm^2^ for (4F), 9.27 mA/cm^2^ for (4I), and 10.07 mA/cm^2^ for (4L). Compounds such as (4D), (4E), and (4F), which contain tolyl groups, suffer from increased steric hindrance. The bulky tolyl group on the benzofuran ring obstructs the effective adsorption of hydrazine on the catalyst surface, leading to decreased catalytic activity. This steric hindrance is reflected in their lower current densities compared to (**4A**). In N_2_H_4_ solution, (4D) has a current density of 11.03 mA/cm^2^, (4E) has 6.27 mA/cm^2^, and (4F) shows 13.14 mA/cm^2^. Additionally, compounds (4G), (4H), and (4I), which have naphthyl groups attached to the benzofuran ring, experience even greater steric hindrance. The larger naphthyl groups prevent effective interaction with hydrazine molecules, further reducing catalytic performance. In N_2_H_4_ solution, their current densities are 16.55 mA/cm^2^ for (4G), 21.40 mA/cm^2^ for (4H), and 9.27 mA/cm^2^ for (4I). Compounds (4J), (4K), and (4L) also demonstrate lower catalytic activity due to steric hindrance from bulky aromatic substituents, such as pentyl, tolyl, and naphthyl groups. Although these compounds maintain a planar structure, (4J) exhibits a higher current density of 31.79 mA/cm^2^ compared to (4K) and (4L), as (4K) and (4L) have thiophene and furan rings, which further reduce their electrophilicity. These electron-donating groups weaken their ability to interact effectively with hydrazine, leading to lower catalytic performance. In N_2_H_4_ solution, (4K) shows 8.78 mA/cm^2^, and (4L) shows 10.07 mA/cm^2^.

**Figure 2:**
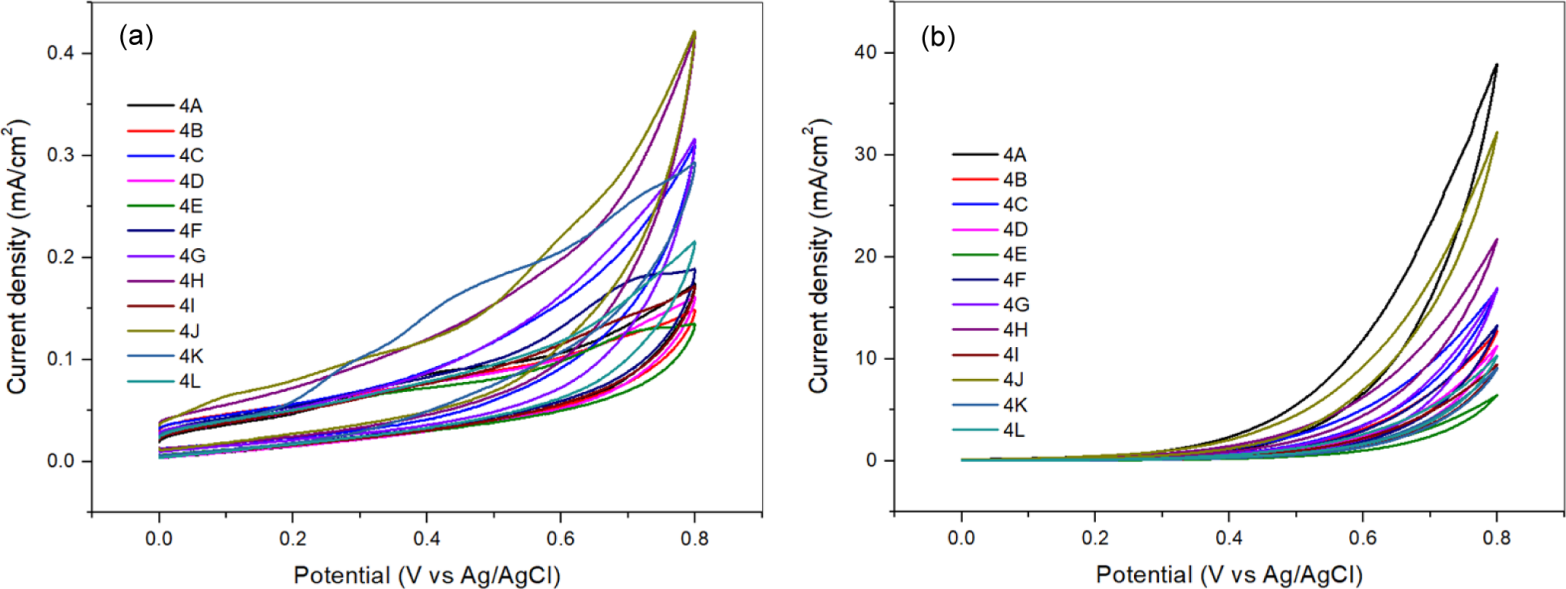
CV measurements of catalysts (a) in 1 M KOH solution and (b) in 1 M KOH + 0.5 M N_2_H_4_ solution.

**Table 1:** Current Density Values for Various Organic Catalysts in Different Electrolyte Solutions for Hydrazine Electrooxidation

**Organic catalyst**	**Current density in KOH solution**	**Current density in (KOH + N**_**2**_ **H**_**4**_ **) solution**	**Current density in N**_**2**_ **H**_**4**_
**4A**	**0.17**	**39.02**	**38.85**
**4B**	0.15	12.75	12.60
**4C**	0.31	16.94	16.63
**4D**	0.16	11.19	11.03
**4E**	0.13	6.40	6.27
**4F**	0.19	13.33	13.14
**4G**	0.32	16.87	16.55
**4H**	0.42	21.82	21.40
**4I**	0.17	9.44	9.27
**4J**	0.42	32.21	31.79
**4K**	0.30	9.08	8.78
**4L**	0.22	10.29	10.07

For better comprehension of the activity of catalyst (**4A**), the effect of hydrazine and KOH concentration on electrooxidation was also examined, and the corresponding CV results are shown in Figure 3. When KOH concentration was fixed at 1 M, varying hydrazine concentrations were tested to determine the optimal value (Figure 3a). The highest catalytic activity was observed at 2 M N_2_H_4_. Similarly, the effect of KOH concentration was evaluated while maintaining a constant 1 M N_2_H_4_ concentration, revealing that 2 M KOH provided the highest catalytic activity (Figure 3a). Due to the excessively high concentrations used in these experiments, CA and EIS measurements were conducted at 1 M KOH and 1 M KOH + 0.5 M N_2_H_4_ to ensure reliable electrochemical characterization.

**Figure 3:**
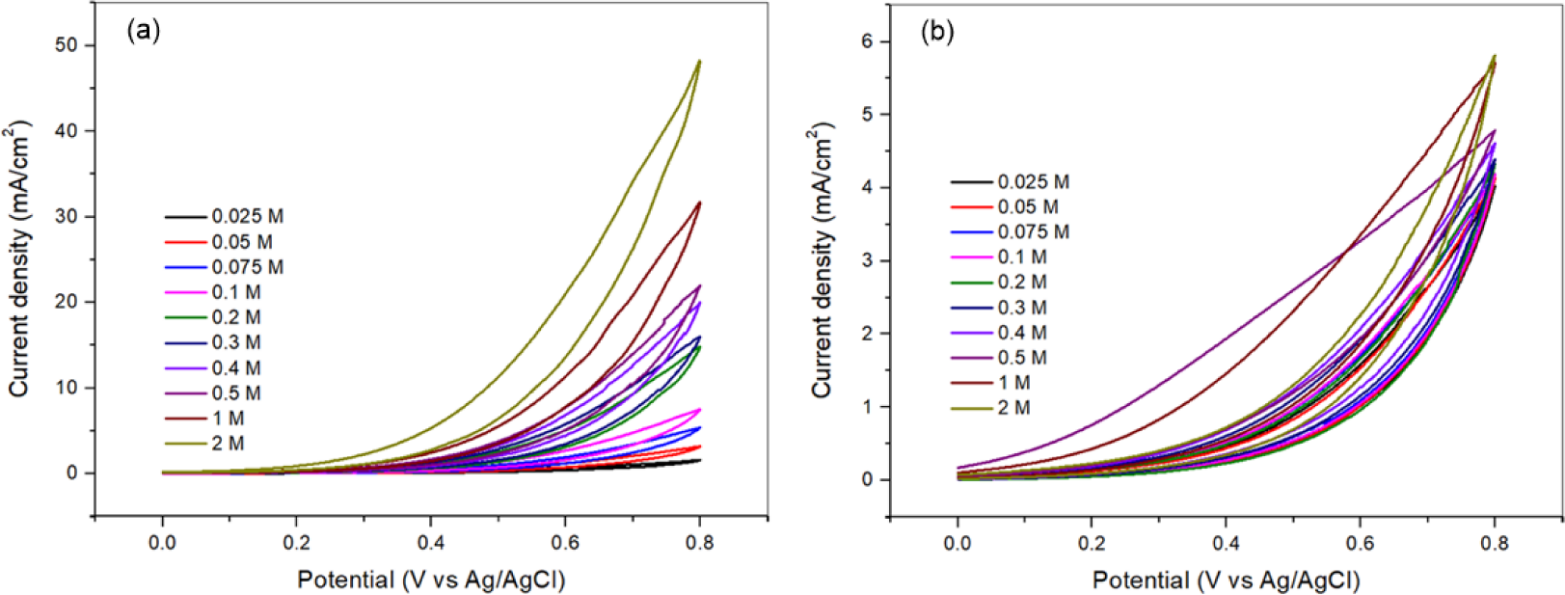
CV results of catalyst (**4A**): (a) Effect of hydrazine concentration in 1 M KOH; (b) Effect of KOH concentration in 1 M N_2_H_4_.

Evaluating the stability of the catalyst (**4A**) is essential for assessing its electrochemical performance. To achieve this, CA measurements were performed for 1000 s at different potentials (0.0, 0.2, 0.4, 0.6, and 0.8 V) in a 1 M KOH + 0.5 M N_2_H_4_ solution. The CA curves at various potentials are shown in Figure 4. The findings reveal that the catalyst (**4A**) exhibits the highest electrocatalytic stability at a potential of 0.8 V.

**Figure 4:**
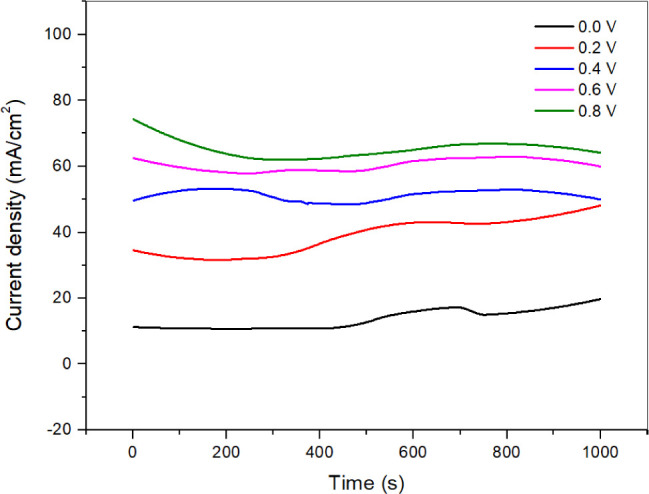
CA measurements of the catalyst (**4A**) at various potentials.

To evaluate the resistance of catalyst (**4A**) to hydrazine electrooxidation, EIS measurements were conducted at potentials ranging from 0.0 to 0.8 V. The resulting data were analyzed using Nyquist plots, as shown in Figure 5. In these plots, the real part of the impedance (Z^i^) is displayed on the *x*-axis, and the imaginary part (-Z^ii^) is shown on the *y*-axis. The plots exhibit a semicircular shape, where the diameter of the semicircle corresponds to charge transfer resistance and electrocatalytic activity. A smaller semicircle diameter indicates reduced charge transfer resistance and enhanced electrocatalytic performance. The Nyquist plots of catalyst (**4A**) at different potentials reveal that at 0.8 V, the catalyst demonstrates the lowest resistance and the highest electrocatalytic activity for hydrazine electrooxidation.

**Figure 5:**
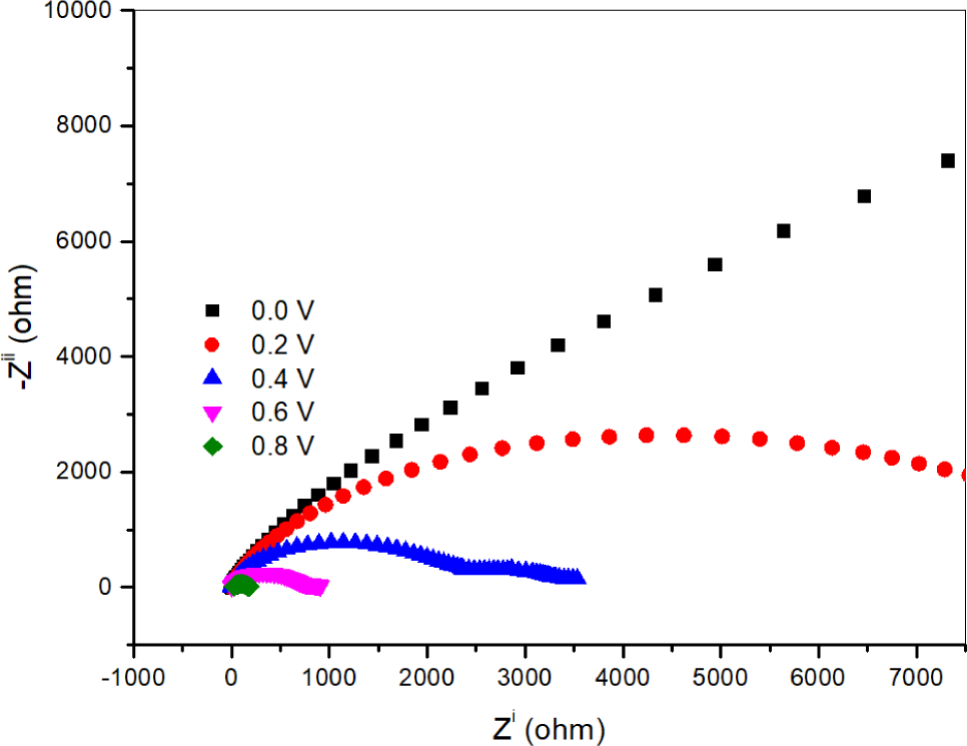
EIS measurements of the catalyst (**4A**) at various potentials.

### 3.3 Computational Studies

#### 3.3.1 DFT Calculations

Figure 6 illustrates the optimized structure, HOMO and LUMO of catalyst (**4A**). The HOMO representation depicts the electronic density distribution of the most energetic occupied molecular orbital, which is essential for understanding electron donation properties. The LUMO, representing the electronic density of the lowest unoccupied molecular orbital, essential for predicting electron acceptance properties.^62,63^ The Δ*E*_gap_ provides insights into catalyst (**4A**) reactivity, stability where a smaller gap suggests higher reactivity.^64,65^ The visualized orbitals, represented by red and green lobes, indicate phase differences and delocalization over specific molecular regions, which influence molecular interactions and chemical behavior.

**Figure 6:**
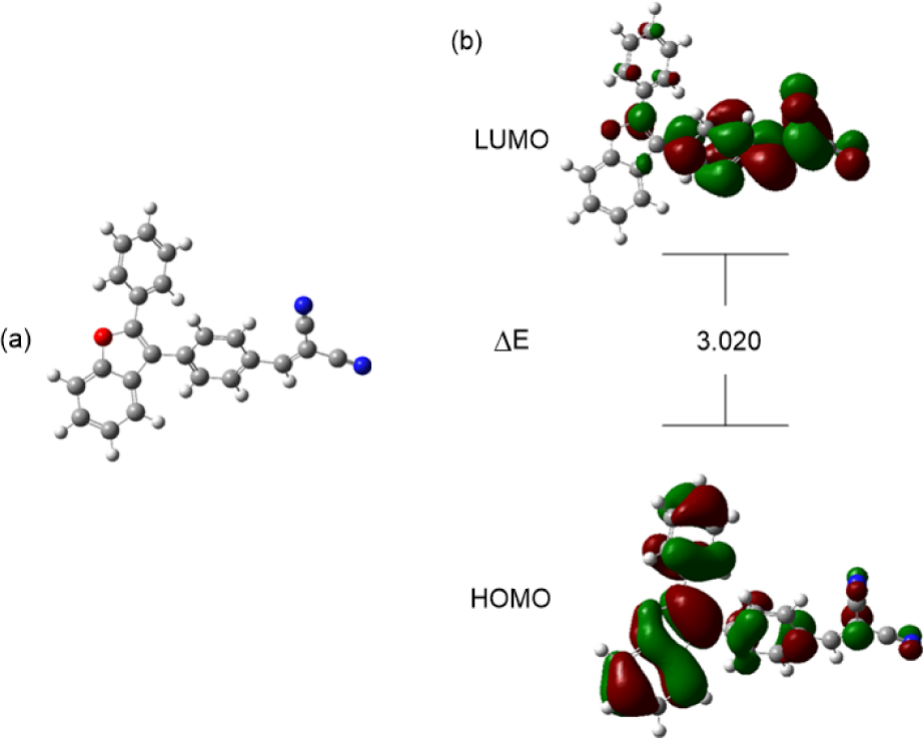
(a) Optimized molecular structure of the catalyst (**4A**). (b) HOMO–LUMO molecular orbitals of the catalyst (**4A**).

Table 2 provides electronic properties for catalyst (**4A**), which give insight into its reactivity and behavior in chemical processes. The catalyst has a HOMO energy level of −5.904 eV, indicating a relatively high electron density in its occupied orbitals, suggesting it may act as a good electron donor. Its LUMO energy level is −2.884 eV, meaning it can readily accept electrons and may function as an electron acceptor in reactions. The Δ*E*_gap_ between the HOMO and LUMO is 3.020 eV, indicating moderate stability and reactivity. The μ is 4.394 eV, suggesting it is polar and may interact strongly with other molecules. With a η of 1.510 eV, catalyst (**4A**) is relatively soft and reactive, while its ω of 6.393 indicates that catalyst (**4A**) is likely to act as an electrophile in electron-transfer reactions, making it a strong candidate for interactions involving electron acceptance.

**Table 2:** Electronic Properties of Catalyst (**4A**)

Catalyst	*E*_HOMO_ (eV)	*E*_LUMO_ (eV)	Δ*E* (eV)	μ (eV)	η (eV)	ω
4A	–5.904	–2.884	3.020	4.394	1.510	6.393

Figure 7 represents the MEP map of the catalyst (**4A**) which provides valuable insights into its electrostatic properties and potential interaction sites. The MEP map represents regions of varying electrostatic potential using a color gradient: red indicate electron-rich regions (negative potential) that are likely to attract electrophiles, while blue represents electron-deficient regions (positive potential) that can attract nucleophiles.^66,67^ The oxygen atom (red sphere) appears to be in a negatively charged region, signifying a high electron density, whereas the nitrogen-containing functional group is in a more positively charged zone, suggesting it may act as an electrophilic site. The MEP visualization aids in understanding molecular reactivity of catalyst (**4A**) and demonstrates its high potential as catalyst.

**Figure 7:**
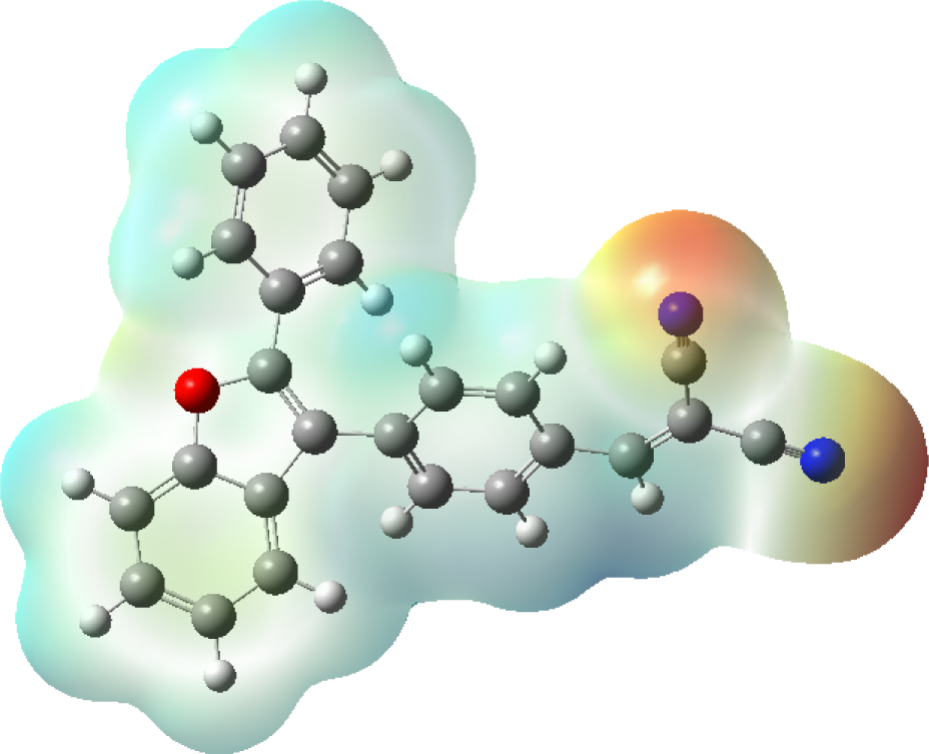
MEP map of catalyst (**4A**).

#### 3.3.2 Topology Analysis

Figure 8 illustrates the ELF and LOL analysis of catalyst (**4A**). The ELF map offers a 3D representation of the spatial distribution of electron pairs, highlighting regions of electron localization. The peaks in the 3D surface plot represent areas with high electron density, indicating where electrons are tightly localized. The color-coded 2D plot below is a more detailed examination, showing areas of high electron localization with a range of colors to represent different electron densities. Red represents higher values, which are areas of higher electron localization, and blue represents lower values, showing less electron localization.^68,69^ The LOL map is the same analysis but with consideration of the localization of individual molecular orbitals. Here the 3D plot again represents regions of maximum electron density values, while below the 2D map shows the electron density’s spatial distribution in the provided orbitals. The map shows regions where the molecular orbitals are most packed, providing information about chemical reactivity and bonding interactions. The color gradient on both maps aids in the visualization of the electron density in a more quantitative manner.^70,71^ These visualizations are crucial in deciding the electronic properties of catalyst (**4A**) and can prove useful in examining its reactivity, bonding character, and interaction with other molecules.

**Figure 8:**
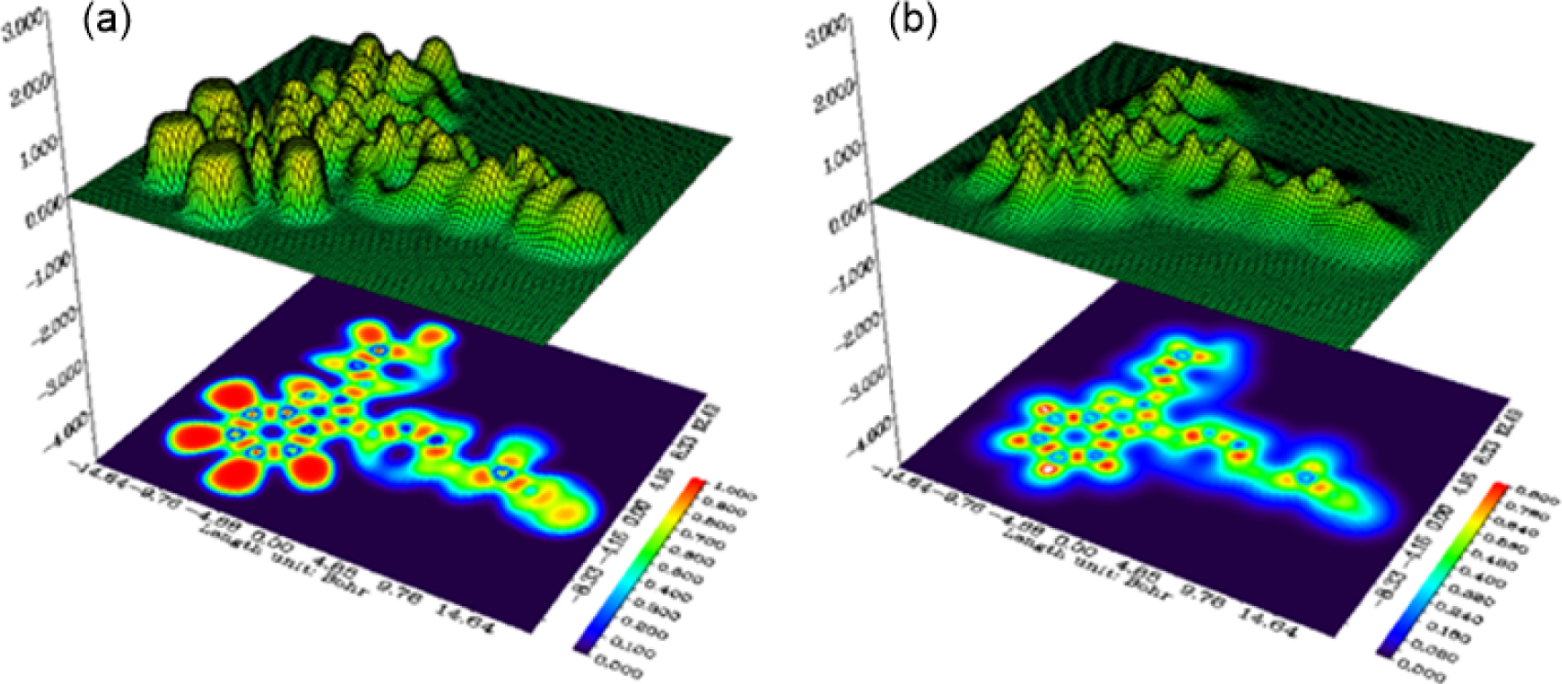
(a) ELF and (b) LOL representation of catalyst (**4A**).

Inspection of the ALIE and IRI maps of catalyst (**4A**) (Figure 9) gives some valuable information on its reactivity and potential intermolecular interactions. The ALIE map highlights regions of low ionization energy, where the electrons are easiest to remove and hence potential targets for electrophilic attack or oxidation.^71–75^ Most importantly, peaks near the cyano groups suggest that they are highly electron-rich and reactive, and peaks near the benzene rings, particularly those on the phenyl group attached to the central furan ring, suggest that these rings are electron-rich as well. The IRI map, which identifies regions favorable for intermolecular interactions,^75,76^ shows wells around the cyano groups, signifying a strong potential for dipole–dipole interactions and hydrogen bonding, and wells around the benzene rings, suggesting potential π-π stacking interactions; furthermore, the furan ring oxygen also represents a potential area for hydrogen bonding.

**Figure 9:**
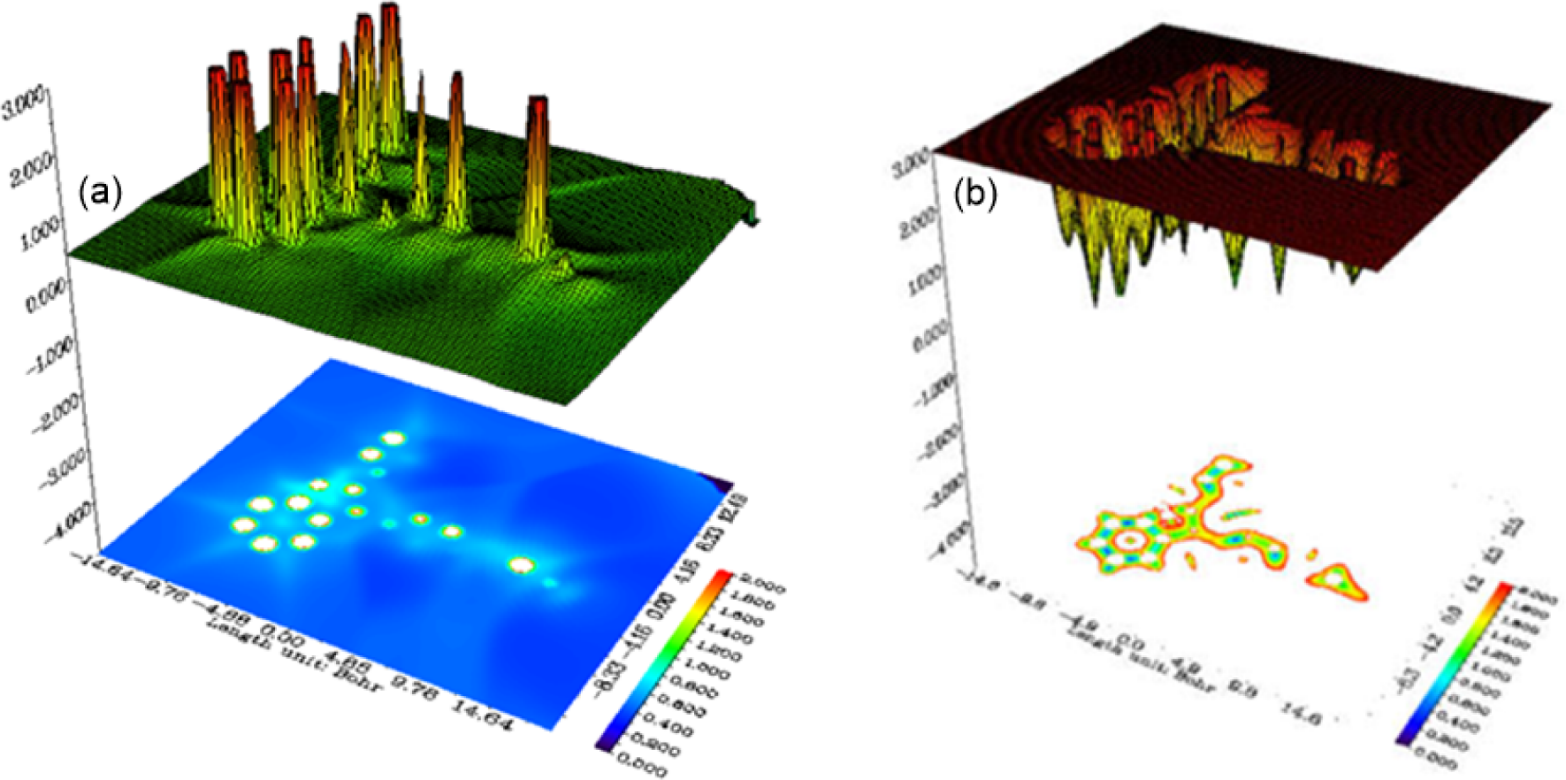
(a) ALIE and (b) IRI of catalyst (**4A**).

From these results, the ELF, LOL, ALIE, and IRI analyses offer significant insights into the electronic properties of the catalyst, particularly emphasizing the role of electron-rich regions, such as the cyano (−CN) groups, in facilitating hydrazine oxidation. The electron-donating nature of these cyano groups increases the electron density on the catalyst’s surface, enhancing its ability to interact with hydrazine molecules. This increased electron density not only aids in the adsorption and activation of hydrazine but also promotes efficient electron transfer during the oxidation process, thereby improving the overall reaction efficiency. The experimental results confirm this enhanced catalytic activity, as the catalyst (**4A**), with its simple and planar structure and the presence of cyano groups, demonstrated significantly higher electrocatalytic performance. This is reflected in the strong correlation between the topology analyses and electrochemical measurements, where the increase in current density supports the findings of the electronic structure analysis. This alignment highlights the crucial role of the cyano groups in improving the catalyst’s electrocatalytic performance, confirming their contribution to the enhanced oxidation process.

The electrooxidation of hydrazine using catalyst (**4A**) follows a multistep proposed mechanism involving adsorption, deprotonation, electron transfer, and the formation of reactive intermediates leading to the final oxidation to nitrogen gas as shown in Figure 10.^77^ Initially, catalyst (**4A**) adsorbs onto the GCE surface, providing active sites for hydrazine that facilitate the oxidation process. The cyano (−CN) groups and the fused bicyclic system of the planar and simple structure in catalyst (**4A**) enhance the adsorption by stabilizing charge transfer interactions. In an alkaline medium, hydrazine undergoes deprotonation, leading to the formation of the hydrazinium ion (N_2_H_5_^+^), which then undergoes an electron transfer process to generate a radical intermediate (N_2_H_4_˙). This radical intermediate, stabilized by the electron-withdrawing cyano groups, undergoes further oxidation, resulting in N–H bond scission and the evolution of nitrogen gas. The extended conjugation in catalyst (**4A**) plays a significant role in facilitating charge delocalization, thereby accelerating the oxidation process and allowing for rapid catalytic turnover.

**Figure 10:**
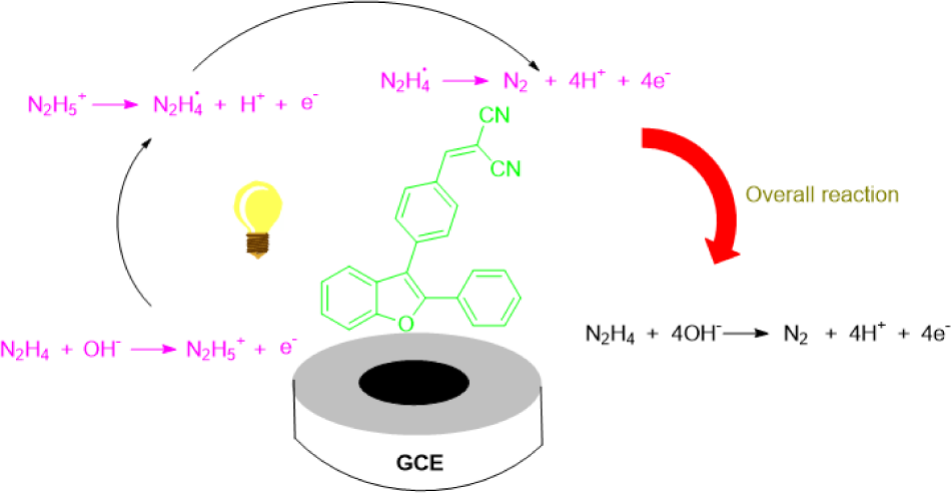
Proposed mechanism for hydrazine electrooxidation using catalyst (**4A**) as a catalyst.

Transition state analysis was conducted to gain a deeper understanding of the reaction mechanism and the energy gaps associated with each elementary step. Figure 11 illustrates the transition state analysis, where the energy gaps (Δ*E*_gap_) for each reaction step were estimated. The calculated energy gaps for the 4A-N_2_H_5_^+^, 4A-N_2_H_4_˙ intermediates, and the 4A-N_2_H_4_ product are 3.440, 3.374, and 2.993 eV, respectively. These values correspond to the energy required to transition from one intermediate to the next, with the decreasing energy gap suggesting a more stable reaction pathway leading to the final product. The values further validate the thermodynamic feasibility of the reaction and the proposed mechanism.

**Figure 11:**
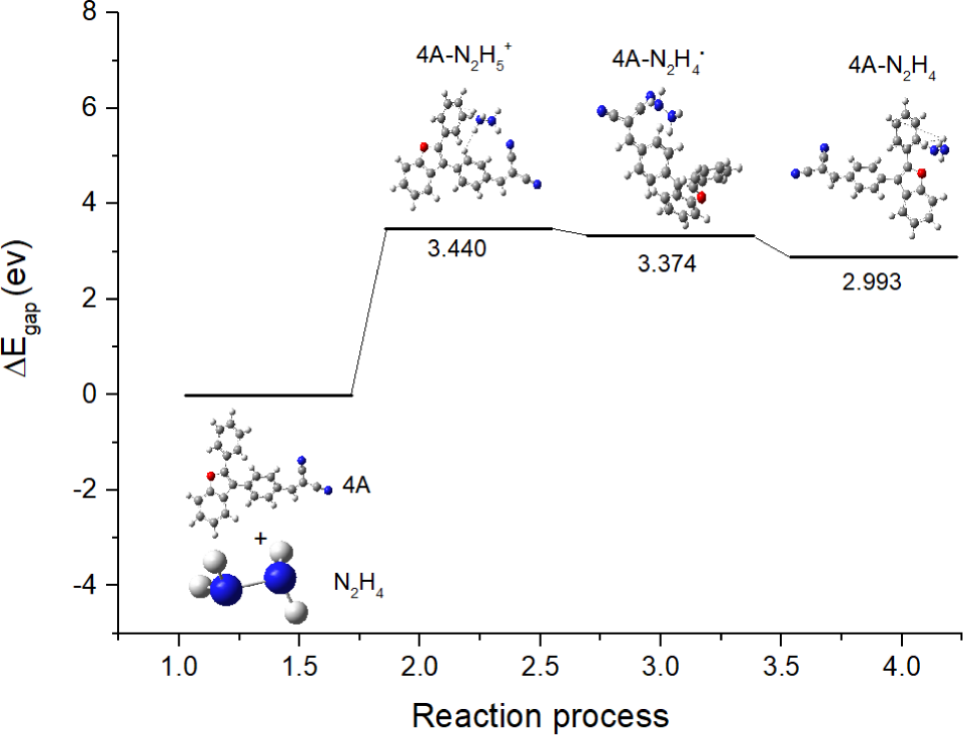
Transition state analysis for hydrazine electrooxidation by catalyst (**4A**).

The overall reaction proceeds as

$$N_{2}H_{4}\;+\;4{\mathrm{OH}}^{-}\;\to \;N_{2}\;+\;4H^{+}\;+\;4e^{-}$$

This mechanism emphasizes the crucial role of catalyst (**4A**) in stabilizing the intermediates and facilitating electron transfer, confirming that catalyst (**4A**) is highly efficient in promoting hydrazine electrooxidation.

Table 3 compares the electrocatalytic performance of catalyst (**4A**) with several other catalysts reported for hydrazine electrooxidation. Catalyst (**4A**), a benzofuran derivative, exhibits a significantly higher current density of 38.85 mA/cm^2^ compared to all other catalysts. This exceptional performance is attributed to its unique molecular structure, featuring an α,β-unsaturated dicyano group attached to a phenyl group, with the benzofuran ring also bonded to a phenyl group. This simple, planar structure promotes efficient electron transfer, enhancing the adsorption and activation of hydrazine molecules on the electrode surface. Compared to metal-based catalysts, catalyst (**4A**) demonstrates superior catalytic activity. For instance, Pd_80_Sn_20_/MWCNT, synthesized through NaBH_4_ reduction, achieves a current density of 13.70 mA/cm^2^, while Pd/MWCNT, also prepared by NaBH_4_ reduction, shows 6.81 mA/cm^2^. Other metal-based catalysts, such as AuPd NanoCs (5.28 mA/cm^2^), prepared by hydrothermal and coreduction methods, respectively. Meta based cataysts display even lower current densities when compared the organic cataysts. These values are considerably lower than the current density of catalyst (**4A**) , emphasizing its remarkable electrocatalytic performance and making it a strong candidate for hydrazine fuel cell applications. In addition, catalyst (**4A**) outperformed than organic-based catalysts such as iodobenzofuran (26.62 mA/cm^2^) and benzothiophene (4.95 mA/cm^2^), with other organic catalysts like artemisinin (3.55 mA/cm^2^) and thymol (3.66 mA/cm^2^) exhibiting even lower current densities. This contrast underscores the limited catalytic efficiency of organic catalysts and highlights catalyst (**4A**)'s exceptional performance. catalyst (**4A**) also presents advantages over metal-based catalysts like gold (Au) and palladium (Pd), particularly in terms of cost, stability, and toxicity. As a metal-free catalyst, catalyst (**4A**) has garnered significant interest for its environmental compatibility, cost-effectiveness, and high efficiency in industrial catalytic processes. Unlike expensive and scarce metals like Au and Pd, catalyst (**4A**) can be synthesized from abundant and accessible materials, making it a more affordable alternative. In terms of stability, while metal-based catalysts are typically stable, they are prone to surface poisoning and leaching, particularly under harsh conditions. In contrast, catalyst (**4A**) exhibits enhanced stability and is less susceptible to these issues, ensuring long-lasting performance in catalytic processes. Additionally, being a metal-free organic catalyst, catalyst (**4A**) is considered safer and less toxic than metal-based catalysts, which can pose environmental and health risks when leached into ecosystems or biological systems. From these results, catalyst (**4A**) offers a more sustainable, cost-effective, and environmentally friendly solution compared to traditional metal-based catalysts. Its high electrocatalytic activity is a result of its unique molecular design, which facilitates efficient electron transfer and enhances hydrazine oxidation. The structural and electronic properties of catalyst (**4A**) make it a promising candidate for future electrochemical applications, particularly in hydrazine fuel cells. The findings in this study underscore the superior electrocatalytic performance of catalyst (**4A**), demonstrating its potential in fuel cell technologies and other related applications.

**Table 3:** Current Density for Reported Catalysts and Our Catalyst (**4A**)

**Catalyst**	**Preparation**	**Current density (mA/cm**^**2**^)	**Reference**
**Pd**_**80**_ **Sn**_**20**_ **/MWCNT**	NaBH_4_ reduction	13.70	^78^
**Pd/MWCNT**	NaBH_4_ reduction	6.81	^79^
**AuPd NanoCs**	Simple coreduction	5.28	^80^
**Iodobenzofuran**	Organic synthesis	26.62	^54^
**Benzothiophene**	Organic synthesis	4.95	^81^
**Artemisinin**	Organic synthesis	3.55	^82^
**Thymol**	Organic synthesis	3.66	^83^
**Benzofuran (4A)**	**Organic synthesis**	**38.85**	**This work**

## 4 Conclusion

In this work, a new and efficient synthetic approach was developed for the preparation of benzofuran derivatives (**4A-4L**) incorporating an α,β-unsaturated dicyano moiety. The four-step sequence, which includes Sonogashira coupling, iodocyclization, Suzuki-Miyaura coupling, and Knoevenagel condensation, proved to be highly efficient, yielding structurally well-defined products. Comprehensive characterization through spectroscopic techniques (^1^H NMR, ^13^C NMR, FT-IR, and mass spectrometry) confirmed the successful synthesis of these compounds. The electrocatalytic performance of the synthesized derivatives was systematically evaluated for hydrazine electrooxidation using CV, CA, and EIS. Catalyst (**4A**) demonstrated the highest catalytic activity, achieving a remarkable current density of 38.70 mA/cm^2^, highlighting its potential as an efficient anode catalyst for hydrazine fuel cells. Furthermore, computational studies, including DFT calculations and topology analysis, offered valuable insights into the electronic properties and catalytic behavior of **4A**, reinforcing its suitability for fuel cell applications.

## Supplementary Material



## Notes

Experimental procedures and spectral data, including ^1^H NMR, ^13^C NMR, FTIR, and mass spectra of the compounds (PDF)
