# Supplementary material for: Enhanced Hydrazine Electrooxidation through Benzofuran Derivatives Containing α,β-Unsaturated Dicyano Groups: Synthesis, Electrocatalytic Performance, and Insights from DFT and Topological Analysis
Source: ACS Omega. 2025 Jun 20;10(25):27182–93. doi: 10.1021/acsomega.5c02472 (PMC12223894; doi:10.1021/acsomega.5c02472)
Supplement: Supplementary file 1 [file ao5c02472_si_001.pdf]

Supporting Information

Enhanced Hydrazine Electrooxidation through Benzofuran Derivatives Containing α,β-Unsaturated Dicyano Groups: Synthesis, Electrocatalytic Performance, and Insights from DFT and Topological Analysis

Bassam A. Najri[a], Katia Mohand Saidi[a], Sefika Kaya[b], Arif Kivrak[a]*, Hilal Kivrak[b]

[a]Department of Chemistry, Science Faculty, Eskisehir Osmangazi University, Eskisehir-26040, Turkey

[b]Department of Chemical Engineering, Faculty of Engineering and Architectural Sciences, Eskisehir Osmangazi University, Eskisehir-26040, Turkey

*Corresponding Author:

Arif Kivrak, Department of Chemistry, Science Faculty, Eskisehir Osmangazi University, Eskisehir-26040, Turkey; (E-mail: arif.kivrak@ogu.edu.tr)

Table S1. Synthesis of (**1a-1d**), (**2a-2d**), (**3a-3l**) and (**4a-4l**) compounds.

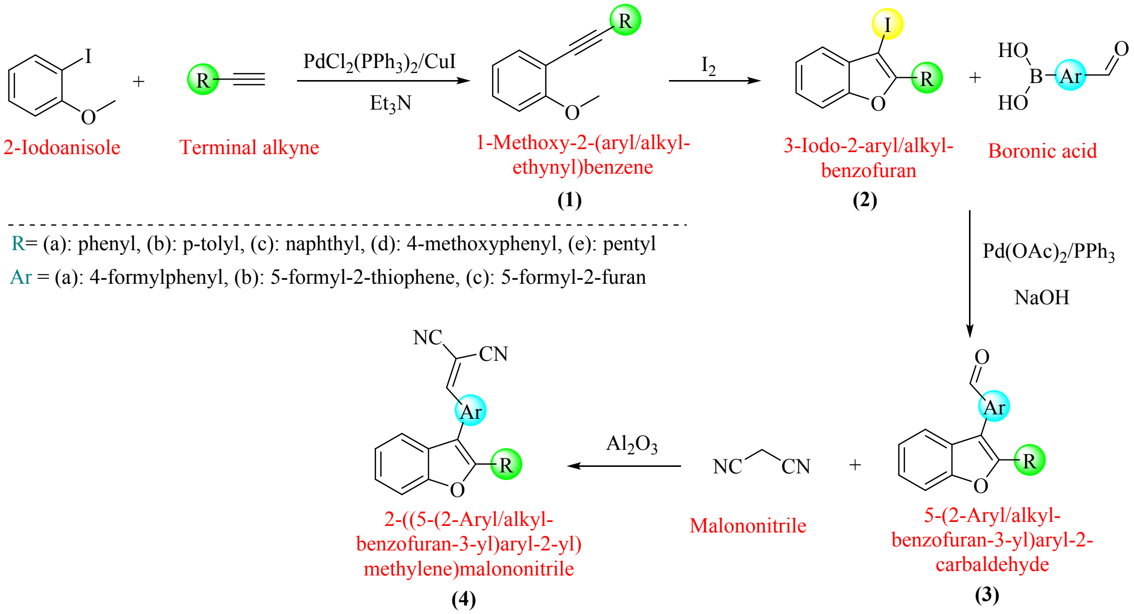

Compound (1)	Compound (2)	Compound (3)	Compound (4)
		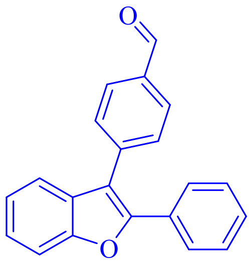	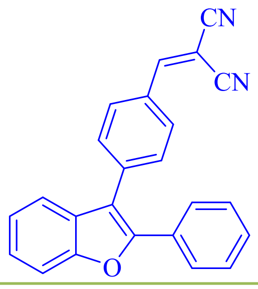
		**3a (96%)**	**4a (97%)**
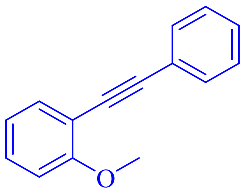	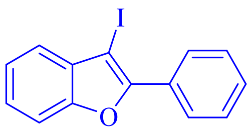	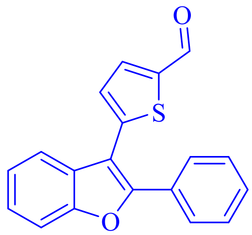	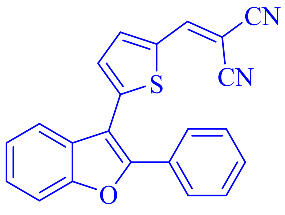
**1a (99%)**	**2a (98%)**	**3b (82%)**	**4b (98%)**
		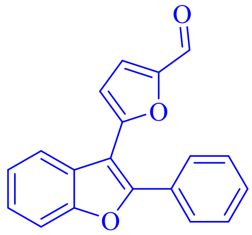	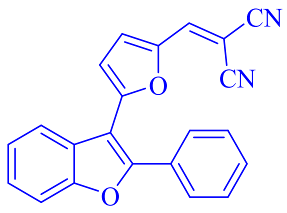
		**3c (80%)**	**4c (96%)**

		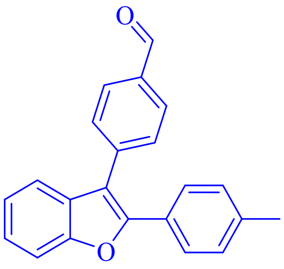	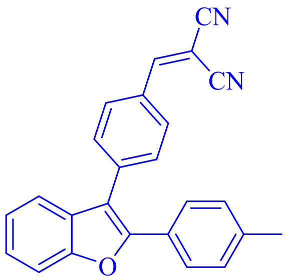
		**3d (99%)**	**4d (99%)**
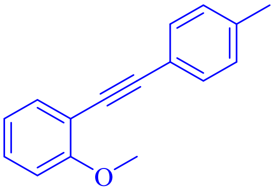	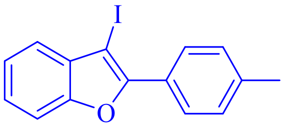	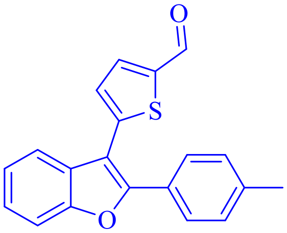	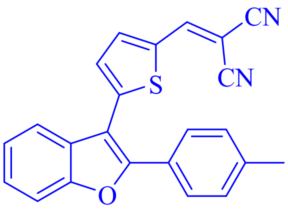
**1b (99%)**	**2b (97%)**	**3e (88%)**	**4e (93%)**
		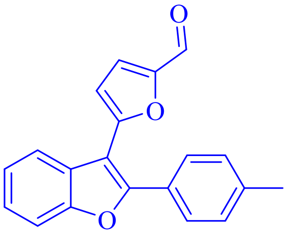	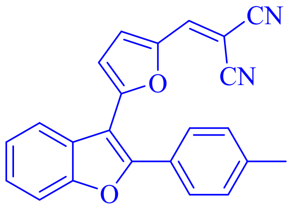
		**3f (81%)**	**4f (95%)**
		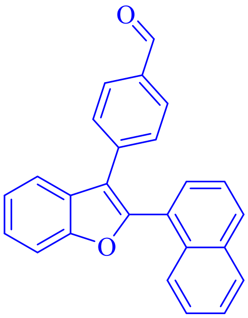	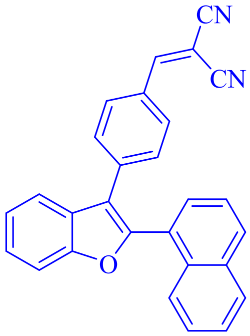
		**3g (99%)**	**4g (96%)**
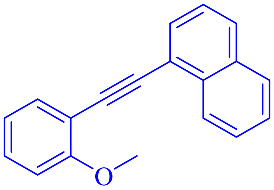	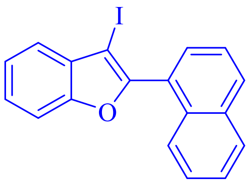	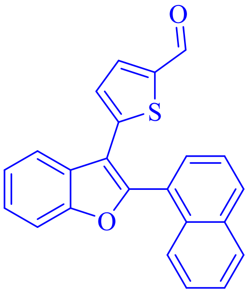	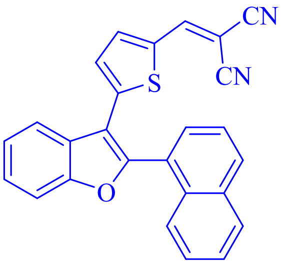
**1c (99%)**	**2c (95%)**	**3h (85%)**	**4h (93%)**

		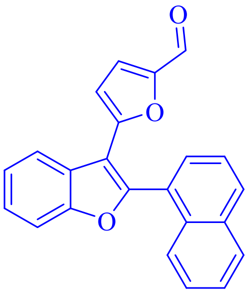	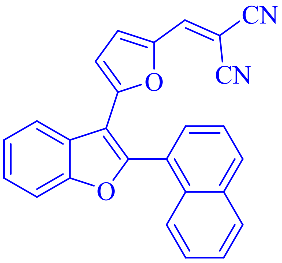
		**3i (83%)**	**4i (91%)**
		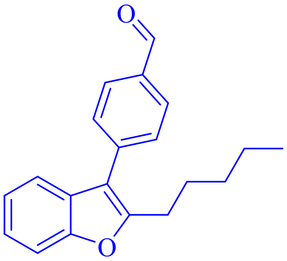	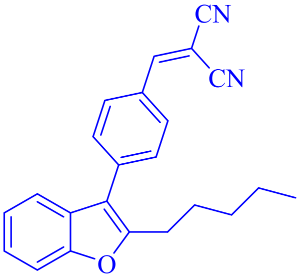
		**3j (95%)**	**4j (98%)**
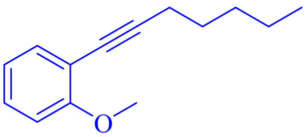	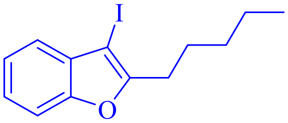	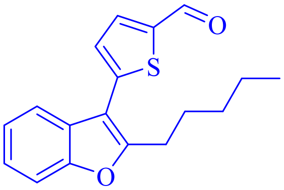	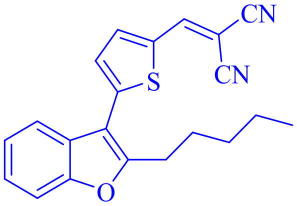
**1d (99%)**	**2d (98%)**	**3k (92%)**	**4k (93%)**
		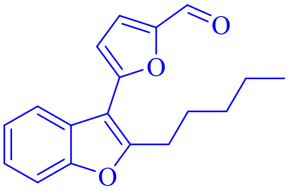	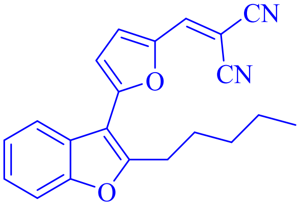
		**3l (90%)**	**4l (96%)**

General

The structure of the compounds (1a-1d), (2a-2d), (3a-3l) and (4a-4l) has been confirmed by ^{1}H and ^{13}C NMR and FT-IR and mass spectrometry. The ^{1}H (500 MHz) and ^{13}C NMR (125 MHz) spectra were recorded in deuterated chloroform ($CDCl_3$) as the solvent using tetramethylsilane (TMS) as an internal standard. Chemical shifts, δ, are reported in ppm relative to the TMS for both ^{1}H and ^{13}C NMR. All coupling constants, *J*, are reported in Hertz (Hz). Multiplicities were given as: s (singlet), d (doublet), t (triplet), q (quartet) and m (multiplet). The mass analysis was performed by using Thermo Q Exactive LC-MS/MS. Analytical thin layer chromatography (TLC) was performed using commercially prepared silica gel plates (Merck 60 F250), and visualization was effected with short wavelength UV light (254 nm).

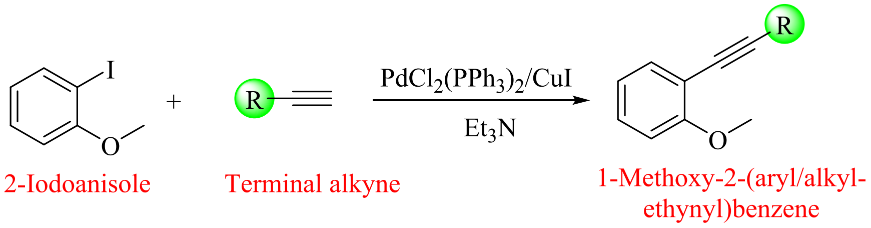

(1)

R= (a): phenyl, (b): p-tolyl, (c): naphthyl, (d): pentyl

General procedure for the synthesis of 1-methoxy-2-(aryl/alkyl-ethynyl)benzene (1a-1d) via Sonogashira coupling. To a solution of 2-iodoanisole (1 equiv.) in tetrahydrofuran (THF) (10 mL), alkyne (1.2 equiv.), triethylamine (Et_3N) (15 mL), palladium (II) bis(triphenylphosphine) dichloride ($PdCl_2(PPh_3)_2$) (2.5 mol%), and copper(I) iodide (CuI) (1 mol%) were added. The mixture was stirred well under an argon atmosphere at room temperature overnight. The reaction mixture was diluted with ethyl acetate (3 x 25 mL) and washed with brine solution. The organic extract was dried over anhydrous magnesium sulphate (Mg_2SO_4). The crude product obtained was purified by silica gel column chromatography using hexane as the eluent to afford the 1-methoxy-2-(aryl/alkyl-ethynyl)benzene (**1a-1d**) compounds.

*1-Methoxy-2-(phenylethynyl)benzene (**1a**).* 2-Iodoanisole (4.27 mmol, 1000 mg), phenylacetylene (5.13 mmol, 524 mg), $PdCl_2(PPh_3)_2$ (0.11 mmol, 75 mg), CuI (0.04, 8 mg), THF (10 mL) and Et_3N (15 mL) were employed to afford 889 mg of product **1a** (99% yield) as a pale yellow solid; Eluent: Hexane; ^{1}H NMR (500 MHz, $CDCl_3$) δ 7.55 (dd, J = 8.1, 1.9 Hz, 2H), 7.49 (dd, J = 7.5, 1.7 Hz, 1H), 7.36 – 7.27 (m, 4H), 6.95 – 6.88 (m, 2H), 3.91 (s, 3H); ^{13}C NMR (125 MHz, $CDCl_3$) δ 160.0, 133.7, 131.8, 129.9, 128.4, 128.2, 123.6, 120.6, 112.5, 110.8, 93.5, 85.8, 56.0.

*1-Methoxy-2-(p-tolylethynyl)benzene (**1b**).* 2-Iodoanisole (4.27 mmol, 1000 mg), p-tolylacetylene (5.13 mmol, 596 mg), $PdCl_2(PPh_3)_2$ (0.11 mmol, 75 mg), CuI (0.04, 8 mg), THF (10 mL) and Et_3N (15 mL) were employed to afford 944 mg of product **1b** (99% yield) as a pale yellow solid; Eluent: Hexane; ^{1}H NMR (500 MHz, $CDCl_3$) δ 7.50 (dd, J = 7.5, 1.8 Hz, 1H), 7.46 (d, J = 8.2 Hz, 2H), 7.32 – 7.26 (m, 1H), 7.14 (d, J = 8.5 Hz, 2H), 6.93 (td, J = 7.5, 1.0 Hz, 1H), 6.89 (d, J = 8.4 Hz, 1H), 3.90 (s, 3H), 2.36 (s, 3H); ^{13}C NMR (125 MHz, $CDCl_3$) δ 160.0, 138.3, 133.6, 131.7, 129.7, 129.1, 120.6, 120.5, 112.7, 110.7, 93.7, 85.1, 55.9, 21.6.

*1-((2-Methoxyphenyl)ethynyl)naphthalene (**1c**).* 2-Iodoanisole (4.27 mmol, 1000 mg), 1-ethynylnaphthalene (5.13 mmol, 780 mg), $PdCl_2(PPh_3)_2$ (0.11 mmol, 75 mg), CuI (0.04, 8 mg), THF (10 mL) and Et_3N (15 mL) were employed to afford 1097 mg of product **1c** (99% yield) as a viscous yellow

oil; Eluent: Hexane; ^{1}H NMR (500 MHz, $CDCl_3$) δ 8.58 (d, J = 8.4 Hz, 1H), 7.87 – 7.81 (m, 2H), 7.78 (dd, J = 7.2, 1.1 Hz, 1H), 7.60 (td, J = 8.0, 1.6 Hz, 2H), 7.60 (td, J = 6.9, 1.2 Hz, 1H), 7.45 (t, J = 8.3, 1H), 7.38 – 7.31 (m, 1H), 6.99 (td, J = 7.5, 1.0 Hz, 1H), 6.95 (d, J = 8.4 Hz, 1H), 3.98 (s, 3H); ^{13}C NMR (125 MHz, $CDCl_3$) δ 160.2, 133.5, 133.4, 133.3, 130.2, 130.0, 128.7, 128.3, 126.8, 126.7, 126.5, 125.4, 121.4, 120.6, 112.7, 110.8, 91.9, 90.9, 56.0.

*1-(Hept-1-yn-1-yl)-2-methoxybenzene (**1d**).* 2-Iodoanisole (4.27 mmol, 1000 mg), 1-heptyne (5.13 mmol, 493 mg), $PdCl_2(PPh_3)_2$ (0.11 mmol, 75 mg), CuI (0.04, 8 mg), THF (10 mL) and Et_3N (15 mL) were employed to afford 1030 mg of product **1d** (99% yield) as a light orange oil; Eluent: Hexane; ^{1}H NMR (500 MHz, $CDCl_3$) δ 7.36 (dd, J = 7.5, 1.8 Hz, 1H), 7.25 – 7.20 (m, 1H), 6.86 (td, J = 7.4, 1.0 Hz, 1H), 6.83 (d, J = 8.4 Hz, 1H), 3.86 (s, 3H), 2.45 (t, J = 7.2 Hz, 2H), 1.66 – 1.58 (m, 2H), 1.48 – 1.40 (m, 2H), 1.38 – 1.30 (m, 2H), 0.91 (t, J = 7.2 Hz, 3H); ^{13}C NMR (125 MHz, $CDCl_3$) δ 159.9, 133.7, 129.0, 120.5, 113.2, 110.5, 94.9, 76.7, 55.9, 31.2, 28.7, 22.4, 19.9, 14.2.

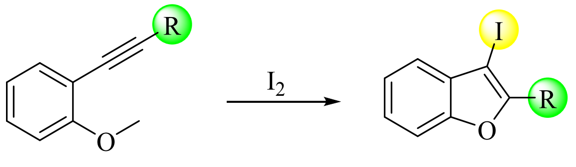

1-Methoxy-2-(aryl/alkyl-ethynyl)benzene **(1)** → 3-Iodo-2-aryl/alkyl-benzofuran **(2)**

R= (a): phenyl, (b): p-tolyl, (c): naphthyl, (d): pentyl

General procedure for the synthesis of 3-iodo-2-aryl-benzofuran (2a-2c) via iodocyclization. To a solution of the compounds (1a-1c) (1 equiv.) in dichloromethane (DCM) (25 mL), iodine (I_2) (1 equiv.) was added, and the mixture was stirred at room temperature for 4 hours under an argon atmosphere. The reaction mixture was quenched with saturated sodium thiosulfate ($Na_2S_2O_3$) solution, and extracted with chloroform ($CHCl_3$) (3 x 25 mL). The combined organic extracts were dried over anhydrous Mg_2SO_4. The crude product was chromatographed on silica gel using hexane/ethylacetate as the eluent to afford the 3-iodo-2-aryl-benzofuran (**2a-2c**).

*3-Iodo-2-phenylbenzofuran (**2a**).* 1-Methoxy-2-(phenylethynyl)benzene (1a) (3.84 mmol, 800 mg), I_2 (7.68 mmol, 1950 mg) and DCM (25 mL) were employed to afford 1210 mg of product **2a** (98% yield) as a pale yellow solid; Eluent: Hexane; ^{1}H NMR (500 MHz, $CDCl_3$) δ 8.21 – 8.15 (m, 2H), 7.52 – 7.40 (m, 5H), 7.39 – 7.29 (m, 2H); ^{13}C NMR (125 MHz, $CDCl_3$) δ 154.0, 153.1, 132.6, 130.1, 129.4, 128.6, 127.6, 125.8, 123.6, 122.0, 111.3, 61.3.

*3-Iodo-2-(p-tolyl)benzofuran (**2b**).* 1-Methoxy-2-(p-tolylethynyl)benzene (1b) (3.88 mmol, 800 mg), I_2 (7.76 mmol, 1969 mg) and DCM (25 mL) were employed to afford 1260 mg of product **2b** (97% yield) as a yellow solid; Eluent: Hexane; ^{1}H NMR (500 MHz, $CDCl_3$) δ 8.08 – 8.05 (m, 2H), 7.48 – 7.42 (m, 2H), 7.37 – 7.28 (m, 4H), 2.42 (s, 3H); ^{13}C NMR (125 MHz, $CDCl_3$) δ 153.9, 153.4, 139.5, 132.6, 129.3, 127.4, 127.3, 125.6, 123.5, 121.8, 111.2, 60.5, 21.6.

*3-Iodo-2-(naphthalen-1-yl)benzofuran (**2c**).* 1-((2-Methoxyphenyl)ethynyl)naphthalene (1c) (3.10 mmol, 800 mg), I_2 (6.19 mmol, 1572 mg) and DCM (25 mL) were employed to afford 1090 mg of product **2c** (95% yield) as a white solid; Eluent: Hexane; ^{1}H NMR (500 MHz, $CDCl_3$) δ 8.00 (d, J = 8.3 Hz, 1H), 7.97 – 7.90 (m, 2H), 7.82 (dd, J = 7.1, 1.1 Hz, 1H), 7.62 – 7.49 (m, 5H), 7.45 – 7.36 (m, 2H); ^{13}C NMR (125 MHz, $CDCl_3$) δ 155.5, 154.8, 133.8, 131.7 (2C),130.7, 130.1, 128.6, 127.4, 127.0, 126.5, 126.2, 125.8, 125.1, 123.7, 121.9, 111.6, 66.3.

General procedure for the synthesis of 3-iodo-2-alkyl-benzofuran (2d) via iodocyclization. To a solution of the compound (1d**)** (1 equiv.) in 1,2-dichloroethane (DCE) (25 mL) sodium bicarbonate ($NaHCO_3$) (3 equiv.) and I_2 (3 equiv.) were added. The reaction mixture was refluxed at 70°C overnight under an argon atmosphere. The reaction mixture was quenched with saturated $Na_2S_2O_3$ solution, and extracted with $CHCl_3$ (3 x 25 mL). The combined organic extracts were dried over anhydrous Mg_2SO_4. The crude product was chromatographed on silica gel using hexane/ethylacetate as the eluent to afford the 3-iodo-2-akyl-benzofuran (**2d**).

*3-Iodo-2-pentylbenzofuran (**2d**).* 1-(Hept-1-yn-1-yl)-2-methoxybenzene (1e) (3.95 mmol, 800 mg), NaHCO3 (11.86 mmol, 997 mg), I_2 (11.86 mmol, 3011 mg) and DCE (25 mL) were employed to afford 1213 mg of product **2d** (98% yield) as a White oil; Eluent: Hexane; ^{1}H NMR (500 MHz, $CDCl_3$) δ 7.40 – 7.36 (m, 1H), 7.33 – 7.30 (m, 1H), 7.28 – 7.24 (m, 2H), 2.85 (t, J = 7.49 Hz, 2H), 1.77 – 1.70 (m, 2H), 1.37 – 1.33 (m, 4H), 0.90 (t, J = 7.15 Hz, 3H); ^{13}C NMR (125 MHz, $CDCl_3$) δ 159.3, 154.3, 131.2, 124.6, 123.2, 120.9, 111.0, 62.6, 31.3, 28.0, 27.6, 22.5, 14.1.

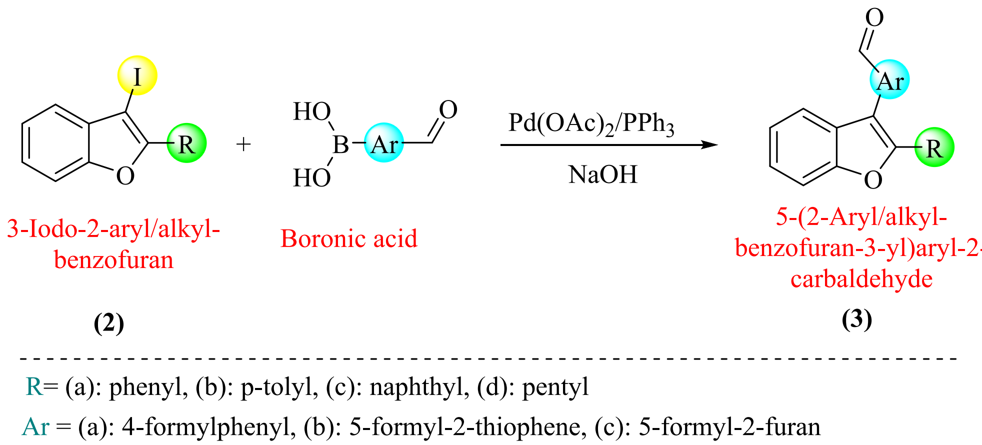

General procedure for the synthesis of 5-(2-aryl/alkyl-benzofuran-3-yl)aryl-2-carbaldehyde (3a-3l) via Suzuki-Miyaura coupling reaction. Into a microwave vial, to a solution of the compounds (2a-2d), in THF (5 mL), palladium (II) acetate ($Pd(OAc)_4$) (10 mol%), triphenylphosphine (PPh_3) (20 mol%), boronic acid (1.5 equiv.) and sodium hydroxide (NaOH) (3 equiv.) were added at room temperature under an argon atmosphere, followed by an addition of water (1 mL). The vial was submerged in the microwave cavity for 45 minutes at 70°C. The post-reaction mixture was cooled to room temperature. The reaction mixture was diluted with ethyl acetate (3 x 25 mL) and washed with brine solution. The organic phases were combined and dried with Mg_2SO_4. The drying agent was filtered off and the crude product purified by silica gel column chromatography using hexane/ethylacetate as the eluent to afford 5-(2-aryl-benzofuran-3-yl)aryl-2-carbaldehyde (3a-3l).

*4-(2-Phenylbenzofuran-3-yl)benzaldehyde (**3a**).* 3-Iodo-2-phenylbenzofuran (2a) (0.62 mmol, 200 mg), $Pd(OAc)_4$ (0.06 mmol, 14 mg), PPh_3 (0.12 mmol, 33 mg), 4-formylphenylboronic acid (0.94 mmol, 141 mg), NaOH (1.87 mmol, 75 mg), THF (5 mL) and water (1 mL) were employed to afford 184 mg of product **3a** (99% yield) as a pale yellow solid; Eluent: hexane/ethylacetate (3/1); ^1H NMR (500 MHz, $CDCl_3$) δ 10.07 (s, 1H), 7.97 (d, J = 8.5 Hz, 2H), 7.69 (d, J = 8.3 Hz, 2H), 7.64 – 7.60 (m, 2H), 7.58 (d, J = 8.3 Hz, 1H), 7.52 (d, J = 7.8 Hz, 1H), 7.38 – 7.31 (m, 4H), 7.30 – 7.25 (m, 1H); ^{13}C NMR (125 MHz, $CDCl_3$) δ 192.0, 154.3, 151.7, 139.8, 135.5, 130.5, 130.4, 130.2, 129.4, 129.1, 128.8, 127.5, 125.2, 123.4, 119.8, 116.4, 111.5; FT-IR: 3050, 3030, 2828, 2736, 1705, 1603, 1453, 1440, 1298, 1257, 1210, 1166, 1069, 964, 840, 813, 776, 752, 698, 609 cm^{-1}.

*5-(2-Phenylbenzofuran-3-yl)thiophene-2-carbaldehyde (**3b**).* 3-Iodo-2-phenylbenzofuran (2a) (0.62 mmol, 200 mg), $Pd(OAc)_4$ (0.06 mmol, 14 mg), PPh_3 (0.12 mmol, 33 mg), 5-formylthiophene-2-boronic acid (0.94 mmol, 146 mg), NaOH (1.87 mmol, 75 mg), THF (5 mL) and water (1 mL) were employed to afford 155 mg of product **3b** (82% yield) as a yellow solid; Eluent: hexane/ethylacetate (3/1); ^1H NMR (500 MHz, $CDCl_3$) δ 9.93 (s, 1H), 7.78 (d, J = 3.8 Hz, 1H), 7.73 – 7.67 (m, 3H), 7.56 (d, J

= 9.1 Hz, 1H), 7.41 – 7.35 (m, 4H), 7.31 (td, *J* = 7.5, 1.0 Hz, 1H), 7.27 (d, *J* = 3.8 Hz, 1H); ^{13}C NMR (125 MHz, $CDCl_3$) δ 182.9, 154.1, 153.1, 144.5, 143.6, 137.2, 129.7 (2C), 129.6, 128.9, 128.3, 127.9, 125.5, 123.7, 120.1, 111.5, 110.1; FT-IR: 3103, 3080, 3064, 2974, 2798, 1669, 1453, 1227, 1207, 1048, 751 cm^{-1}.

*5-(2-Phenylbenzofuran-3-yl)furan-2-carbaldehyde (**3c**).* 3-Iodo-2-phenylbenzofuran (2a) (0.62 mmol, 200 mg), $Pd(OAc)_4$ (0.06 mmol, 14 mg), PPh_3 (0.12 mmol, 33 mg), 5-formylfuran-2-boronic acid (0.94 mmol, 131 mg), NaOH (1.87 mmol, 75 mg), THF (5 mL) and water (1 mL) were employed to afford 144 mg of product **3c** (80% yield) as a red solid; Eluent: hexane/ethylacetate (3/1); ^{1}H NMR (500 MHz, $CDCl_3$) δ 9.66 (s, 1H), 8.05 – 7.97 (m, 1H), 7.82 – 7.80 (m, 2H), 7.54 (dd, *J* = 7.0, 1.2 Hz, 1H), 7.49 – 7.43 (m, 3H), 7.40 – 7.33 (m, 2H), 7.32 (d, *J* = 3.7 Hz, 1H), 6.72 (d, *J* = 3.7 Hz, 1H); ^{13}C NMR (125 MHz, $CDCl_3$) δ 177.2, 154.4, 154.2, 154.0, 152.0, 130.1, 130.0, 128.9, 128.3, 127.1, 125.6, 125.4, 124.0, 121.6, 111.4, 110.5, 107.3; FT-IR: 3064, 2926, 2852, 1674, 1596, 1517, 1454, 1365, 1256, 1197, 1071, 972, 898, 750, 695, 619, 496 cm^{-1}.

*4-(2-(p-Tolyl)benzofuran-3-yl)benzaldehyde (**3d**).* 3-Iodo-2-(p-tolyl)benzofuran (2b) (0.60 mmol, 200 mg), $Pd(OAc)_4$ (0.06 mmol, 13 mg), PPh_3 (0.12 mmol, 31 mg), 4-formylphenylboronic acid (0.90 mmol, 135 mg), NaOH (1.80 mmol, 72 mg), THF (5 mL) and water (1 mL) were employed to afford 183 mg of product **3d** (98% yield) as a pale yellow solid; Eluent: hexane/ethylacetate (3/1); ^{1}H NMR (500 MHz, $CDCl_3$) δ 10.07 (s, 1H), 7.96 (d, *J* = 8.5 Hz, 2H), 7.68 (d, *J* = 8.3 Hz, 2H), 7.56 (d, *J* = 8.3 Hz, 1H), 7.51 (dd, *J* = 8.1, 1.9 Hz, 3H), 7.37 – 7.32 (m, 1H), 7.28 – 7.24 (m, 1H), 7.14 (d, *J* = 7.9 Hz, 2H), 2.36 (s, 3H); ^{13}C NMR (125 MHz, $CDCl_3$) δ 192.0, 154.2, 152.0, 140.0, 139.2, 135.4, 130.5, 130.4, 129.5, 129.4, 127.4, 127.3, 124.9, 123.3, 119.6, 115.7, 111.4, 21.5; FT-IR: 3088, 3060, 3034, 2960, 2916, 2834, 2737, 1700, 1605, 1454, 1211, 1069, 964, 843, 821, 750 cm^{-1}.

*5-(2-(p-Tolyl)benzofuran-3-yl)thiophene-2-carbaldehyde (**3e**).* 3-iodo-2-(p-tolyl)benzofuran (2b) (0.60 mmol, 200 mg), $Pd(OAc)_4$ (0.06 mmol, 13 mg), PPh_3 (0.12 mmol, 31 mg), 5-formylthiophene-2-boronic acid (0.90 mmol, 140 mg), NaOH (1.80 mmol, 72 mg), THF (5 mL) and water (1 mL) were employed to afford 168 mg of product **3e** (88% yield) as a yellow solid; Eluent: hexane/ethylacetate (3/1); ^{1}H NMR (500 MHz, $CDCl_3$) δ 9.92 (s, 1H), 7.77 (d, *J* = 3.8 Hz, 1H), 7.69 – 7.66 (m, 1H), 7.61 – 7.58 (m, 2H), 7.54 (dt, *J* = 8.2, 1.0 Hz, 1H), 7.37 – 7.33 (m, 1H), 7.30 (td, *J* = 7.4, 1.0 Hz, 1H), 7.27 (d, *J* = 3.8 Hz, 1H), 7.20 (d, *J* = 7.8 Hz, 2H), 2.38 (s, 3H).; ^{13}C NMR (125 MHz, $CDCl_3$) δ 182.9, 154.1, 153.5, 144.8, 143.5, 139.9, 137.2, 129.6, 128.9, 128.2, 127.9, 126.8, 125.3, 123.7, 119.9, 111.5, 109.5, 21.6; FT-IR: 3082, 3057, 3030, 2972, 2924, 2817, 1658, 1600, 1505, 1475, 1450, 1419, 1209, 1050, 756 cm^{-1}.

*5-(2-(p-Tolyl)benzofuran-3-yl)furan-2-carbaldehyde (**3f**).* 3-Iodo-2-(p-tolyl)benzofuran (2b) (0.60 mmol, 200 mg), $Pd(OAc)_4$ (0.06 mmol, 13 mg), PPh_3 (0.12 mmol, 31 mg), 5-formylfuran-2-boronic acid (0.90 mmol, 126 mg), NaOH (1.80 mmol, 72 mg), THF (5 mL) and water (1 mL) were employed to

afford 147 mg of product **3f** (81% yield) as a red solid; Eluent: hexane/ethylacetate (3/1); ^{1}H NMR (500 MHz, $CDCl_3$) 9.66 (s, 1H), 8.04 – 7.96 (m, 1H), 7.70 (d, *J* = 8.3 Hz, 2H), 7.54 – 7.51 (m, 1H), 7.38 – 7.32 (m, 2H), 7.32 – 7.26 (m, 3H), 6.72 (d, *J* = 3.7 Hz, 1H), 2.42 (s, 3H); ^{13}C NMR (125 MHz, $CDCl_3$) δ 177.1, 154.8, 154.3, 154.1, 151.9, 140.8, 129.6, 128.2, 127.2, 127.1, 125.4 (2C), 123.9, 121.5, 111.4, 110.3, 106.8, 21.7; FT-IR: 2924, 2853, 2804, 1664, 1595, 1518, 1449, 1385, 1281, 1196, 1080, 970, 743, 499 cm^{-1}.

4-(2-(Naphthalen-1-yl)benzofuran-3-yl)benzaldehyde (3g). 3-Iodo-2-(naphthalen-1-yl)-benzofuran (2c) (0.54 mmol, 200 mg), $Pd(OAc)_4$ (0.05 mmol, 12 mg), PPh_3 (0.11 mmol, 28 mg), 4-formylphenylboronic acid (0.81 mmol, 121 mg), NaOH (1.62 mmol, 65 mg), THF (5 mL) and water (1 mL) were employed to afford 186 mg of product **3g** (99% yield) as a viscous yellow oil; Eluent: hexane/ethylacetate (3/1); ^{1}H NMR (500 MHz, $CDCl_3$) δ 9.94 (s, 1H), 7.96 – 7.93 (m, 2H), 7.91 (dd, *J* = 8.2, 1.3 Hz, 1H), 7.85 – 7.80 (m, 1H), 7.78 – 7.75 (m, 2H), 7.68 – 7.60 (m, 1H), 7.54 – 7.49 (m, 4H), 7.46 – 7.37 (m, 4H); ^{13}C NMR (125 MHz, $CDCl_3$) δ 192.0, 155.0, 152.7, 139.3, 134.9, 133.9, 131.8, 130.4, 130.2, 129.8, 129.6, 128.6, 128.0, 127.7, 127.0, 126.5, 125.9, 125.3, 125.2, 123.6, 120.1, 118.8, 111.9; FT-IR: 3046, 2838, 2743, 1692, 1606, 1453, 1205, 1023, 778 cm^{-1}.

5-(2-(Naphthalen-1-yl)benzofuran-3-yl)thiophene-2-carbaldehyde (3h). 3-Iodo-2 (naphthalen-1-yl)benzofuran (2c) (0.54 mmol, 200 mg), $Pd(OAc)_4$ (0.05 mmol, 12 mg), PPh_3 (0.11 mmol, 28 mg), 5-formylthiophene-2-boronic acid (0.81 mmol, 126 mg), NaOH (1.62 mmol, 65 mg), THF (5 mL) and water (1 mL) were employed to afford 162 mg of product **3h** (85% yield) as a brown solid; Eluent: hexane/ethylacetate (3/1); ^{1}H NMR (500 MHz, $CDCl_3$) δ 9.78 (s, 1H), 8.03 – 7.99 (m, 2H), 7.93 (d, *J* = 8.2 Hz, 1H), 7.76 (d, *J* = 8.5 Hz, 1H), 7.69 (dd, *J* = 7.1, 1.1 Hz, 1H), 7.63 – 7.60 (m, 1H), 7.57 – 7.50 (m, 3H), 7.47 – 7.41 (m, 3H), 7.00 (d, *J* = 3.9 Hz, 1H); ^{13}C NMR (125 MHz, $CDCl_3$) δ 182.8, 154.9, 153.4, 144.5, 142.3, 136.9, 133.9, 131.8, 131.1, 129.8, 128.7, 127.4, 127.3, 127.1, 126.8, 126.6, 125.6, 125.4, 125.3, 123.9, 120.6, 113.3, 111.9; FT-IR: 3047, 2924, 2852, 2808, 1657, 1451, 1228, 761 cm^{-1}.

5-(2-(Naphthalen-1-yl)benzofuran-3-yl)furan-2-carbaldehyde (3i). 3-Iodo-2-(naphthalen-1-yl)-benzofuran (2c) (0.54 mmol, 200 mg), $Pd(OAc)_4$ (0.05 mmol, 12 mg), PPh_3 (0.11 mmol, 28 mg), 5-formylfuran-2-boronic acid (0.81 mmol, 113 mg), NaOH (1.62 mmol, 65 mg), THF (5 mL) and water (1 mL) were employed to afford 152 mg of product **3i** (83% yield) as a red solid; Eluent: hexane/ethylacetate (3/1); ^{1}H NMR (500 MHz, $CDCl_3$) δ 9.57 (s, 1H), 8.35 – 8.31 (m, 1H), 8.05 (d, *J* = 8.3 Hz, 1H), 7.96 (d, *J* = 8.2 Hz, 1H), 7.78 – 7.74 (m, 2H), 7.62 – 7.58 (m, 2H), 7.56 – 7.52 (m, 1H), 7.47 – 7.43 (m, 3H), 7.07 (d, *J* = 3.8 Hz, 1H), 5.95 (d, *J* = 3.8 Hz, 1H); ^{13}C NMR (125 MHz, $CDCl_3$) δ 177.0, 154.8, 154.4, 153.8, 151.6, 133.8, 131.5, 131.1, 129.4, 128.7, 127.6, 127.4, 126.7, 125.9, 125.7, 125.5, 125.4, 124.2, 122.4, 112.0, 111.6, 110.4, 109.7; FT-IR: 3053, 2824, 1673, 1615, 1521, 1451, 1394, 1263, 1187, 1103, 1029, 965, 898, 770 cm^{-1}.

*4-(2-Pentylbenzofuran-3-yl)benzaldehyde (**3j**).* 3-Iodo-2-pentylbenzofuran (2d) (0.68 mmol, 200 mg), $Pd(OAc)_4$ (0.07 mmol, 15 mg), PPh_3 (0.14 mmol, 36 mg), 4-formylphenylboronic acid (1.03 mmol, 154 mg), NaOH (2.05 mmol, 82 mg), THF (5 mL) and water (1 mL) were employed to afford 190 mg of product **3j** (95% yield) as a white oil; Eluent: hexane/ethylacetate (3/1); ^{1}H NMR (500 MHz, $CDCl_3$) δ 10.06 (s, 1H), 7.99 (d, *J* = 8.2 Hz, 2H), 7.66 (d, *J* = 8.2 Hz, 2H), 7.55 (d, *J* = 8.5 Hz, 1H), 7.48 (d, *J* = 7.2 Hz, 1H), 7.31 – 7.23 (m, 2H), 2.87 (t, *J* = 7.68 Hz, 2H), 1.83 – 1.75 (m, 2H), 1.36 – 1.29 (m, 4H), 0.87 (t, *J* = 7.2 Hz, 3H); ^{13}C NMR (125 MHz, $CDCl_3$) δ 191.9, 156.4, 154.2, 139.8, 135.0, 130.3, 129.5, 128.2, 124.1, 123.0, 119.3, 116.1, 111.2, 31.6, 29.8, 27.0, 22.5, 14.1; FT-IR: 3058, 2928, 2858, 2733, 1700, 1607, 1563, 1455, 1379, 1304, 1281, 1251, 1213, 1168, 1106, 1013, 966, 833, 747 cm^{-1}.

*5-(2-Pentylbenzofuran-3-yl)thiophene-2-carbaldehyde (**3k**).* 3-Iodo-2-pentylbenzofuran (2d) (0.68 mmol, 200 mg), $Pd(OAc)_4$ (0.07 mmol, 15 mg), PPh_3 (0.14 mmol, 36 mg), 5-formylthiophene-2-boronic acid (1.03 mmol, 160 mg), NaOH (2.05 mmol, 82 mg), THF (5 mL) and water (1 mL) were employed to afford 188 mg of product **3k** (92% yield) as a light orange oil; Eluent: hexane/ethylacetate (3/1); ^{1}H NMR (500 MHz, $CDCl_3$) δ 9.92 (s, 1H), 7.81 (d, *J* = 3.9 Hz, 1H), 7.76 – 7.72 (m, 1H), 7.49 – 7.45 (m, 1H), 7.33 (d, *J* = 3.9 Hz, 1H), 7.32 – 7.26 (m, 2H), 2.99 (t, *J* = 7.63 Hz, 2H), 1.84 – 1.77 (m, 2H), 1.40 – 1.32 (m, 4H), 0.89 (t, *J* = 7.2 Hz, 3H); ^{13}C NMR (125 MHz, $CDCl_3$) δ 182.9, 158.1, 154.0, 145.0, 142.2, 137.3, 127.3, 126.3, 124.6, 123.4, 119.7, 111.3, 110.3, 31.6, 27.9, 27.6, 22.5, 14.1; FT-IR: 3071, 2930, 2860, 2736, 1668, 1592, 1455, 1381, 1213, 1107, 1053, 925, 810, 747, 671, 507 cm^{-1}.

*5-(2-Pentylbenzofuran-3-yl)furan-2-carbaldehyde (**3l**).* 3-Iodo-2-pentylbenzofuran (2d) (0.68 mmol, 200 mg), $Pd(OAc)_4$ (0.07 mmol, 15 mg), PPh_3 (0.14 mmol, 36 mg), 5-formylfuran-2-boronic acid (1.03 mmol, 144 mg), NaOH (2.05 mmol, 82 mg), THF (5 mL) and water (1 mL) were employed to afford 174 mg of product **3l** (90% yield) as a light orange oil; Eluent: hexane/ethylacetate (3/1); ^{1}H NMR (500 MHz, $CDCl_3$) δ 9.66 (s, 1H), 7.90 – 7.87 (m, 1H), 7.48 – 7.44 (m, 1H), 7.37 (d, *J* = 3.7 Hz, 1H), 7.33 – 7.29 (m, 2H), 6.74 (d, *J* = 3.7 Hz, 1H), 3.09 (t, *J* = 7.58 Hz, 2H), 1.85 – 1.79 (m, 2H), 1.42 – 1.33 (m, 4H), 0.89 (t, *J* = 7.2 Hz, 3H); ^{13}C NMR (125 MHz, $CDCl_3$) δ 176.9, 159.5, 154.6, 154.0, 151.5, 126.0, 124.6, 123.6, 120.5, 111.2 (2C), 108.8, 107.1, 31.6, 28.3, 27.7, 22.5, 14.1; FT-IR: 3073, 3055, 2969, 2928, 2872, 1665, 1600, 1517, 1454, 1435, 1387, 1093, 1049, 1032 cm^{-1}.

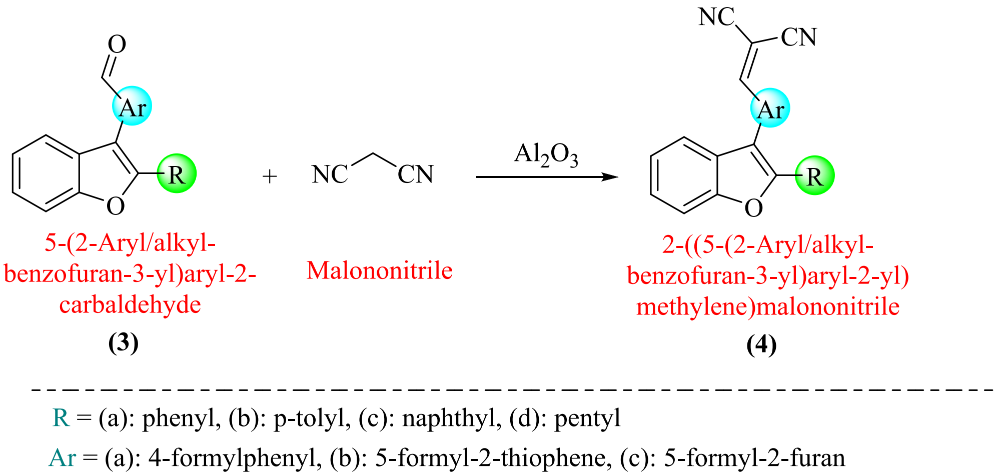

General procedure for the synthesis of 2-((5-(2-aryl/alkyl-benzofuran-3-yl)aryl-2-yl)methylene)malononitrile (4a-4l) via Knoevenagel condensation reaction. A mixture of (3a-3L) (1 equiv.), malononitrile (4 equiv.), aluminium oxide (Al_2O_3) (1 equiv.) in toluene (5 mL) was stirred at room temperature overnight under an argon atmosphere. After completion, the mixture was filtered with $CHCl_3$. The filtrate was purified by silica gel column chromatography using hexane/ethylacetate as the eluent to obtain pure 2-((5-(2-aryl/alkyl-benzofuran-3-yl)aryl-2 yl)methylene)malononitrile (**4a-4l**).

2-(4-(2-Phenylbenzofuran-3-yl)benzylidene)malononitrile ***(4a)***. 4-(2-Phenylbenzofuran-3-yl)benzaldehyde (3a) (0.17 mmol, 50 mg), malononitrile (0.67 mmol, 44 mg), Al_2O_3 (0.17 mmol, 17 mg) and toluene (5 mL) were employed to afford 56 mg of product **4a** (97% yield) as a bright yellow solid; Eluent: hexane/ethylacetate (19/1); ^{1}H NMR (500 MHz, $CDCl_3$) δ 7.99 (d, J = 8.5 Hz, 2H), 7.79 (s, 1H), 7.69 (d, J = 8.6 Hz, 2H), 7.63 – 7.56 (m, 3H), 7.54 (dt, J = 7.7, 1.0 Hz, 1H), 7.40 – 7.33 (m, 4H), 7.31 – 7.27 (m, 1H); ^{13}C NMR (125 MHz, $CDCl_3$) δ 159.2, 154.3, 152.3, 140.3, 131.6, 130.8, 130.0, 129.9, 129.4, 129.0, 128.9, 127.7, 125.4, 123.6, 119.7, 115.9, 114.0, 112.9, 111.6, 82.3; FT-IR: 3036, 2880, 2226, 1591, 1452, 1208, 1066, 966, 841, 744, 509 cm^{-1}; LC-MS/MS calculated $C_{24}H_{14}N_2O$: 347.11789 $[M+H]^+$, found: 347.11859 $[M+H]^+$.

2-((5-(2-Phenylbenzofuran-3-yl)thiophen-2-yl)methylene)malononitrile ***(4b)***. 5-(2-Phenylbenzofuran-3-yl)thiophene-2-carbaldehyde (3b) (0.16 mmol, 50 mg), malononitrile (0.66 mmol, 43 mg), Al_2O_3 (0.16 mmol, 17 mg) and toluene (5 mL) were employed to afford 57 mg of product **4b** (98% yield) as an orange solid; Eluent: hexane/ethylacetate (19/1); ^{1}H NMR (500 MHz, $CDCl_3$) δ 7.80 (s, 1H), 7.77 (d, J = 4.0 Hz, 1H), 7.72 – 7.68 (m, 3H), 7.57 (dt, J = 8.2, 0.9 Hz, 1H), 7.45 – 7.37 (m, 4H), 7.34 (td, J = 7.5, 1.1 Hz, 1H), 7.29 (d, J = 4.0 Hz, 1H); ^{13}C NMR (125 MHz, $CDCl_3$) δ 154.2, 153.9, 150.5, 147.0, 139.1, 135.1, 130.1, 129.5, 129.0, 128.7, 128.2, 128.1, 125.8, 124.0, 120.0, 114.2, 113.4, 111.7, 109.6,

77.4; FT-IR: 3122, 3020, 2921, 2851, 2217, 1555, 1438, 1360, 1310, 1165, 816, 735, 691, 611 cm^{-1}; LC-MS/MS calculated $C_{22}H_{12}N_2OS$: 353.07431 $[M+H]^+$, found: 353.07483 $[M+H]^+$.

2-((5-(2-Phenylbenzofuran-3-yl)furan-2-yl)methylene)malononitrile (4c). 5-(2-Phenylbenzofuran-3-yl)furan-2-carbaldehyde (3c) (0.17 mmol, 50 mg), malononitrile (0.69 mmol, 46 mg), Al_2O_3 (0.17 mmol, 18 mg) and toluene (5 mL) were employed to afford 56 mg of product **4c** (96% yield) as a yellow solid; Eluent: hexane/ethylacetate (19/1); ^1H NMR (500 MHz, $CDCl_3$) δ 7.98 – 7.87 (m, 2H), 7.82 – 7.77 (m, 2H), 7.67 – 7.60 (m, 2H), 7.54 – 7.49 (m, 3H), 7.48 – 7.37 (m, 3H); ^{13}C NMR (125 MHz, $CDCl_3$) δ 159.2, 155.1, 153.3, 139.9, 134.0, 131.3, 130.7, 129.9, 128.7, 127.1, 126.6, 125.7, 125.4, 125.3, 123.8, 120.0, 114.0, 112.9, 112.0, 81.9; FT-IR: 2921, 2852, 2230, 1576, 1463, 1372, 1179, 1105, 1064, 960, 856, 743, 615, 543 cm^{-1}; LC-MS/MS calculated $C_{22}H_{12}N_2O_2$: 337.09715 $[M+Na]^+$, found: 337.09439 $[M+Na]^+$.

2-(4-(2-(p-Tolyl)benzofuran-3-yl)benzylidene)malononitrile (4d). 4-(2-(p-Tolyl)benzofuran-3-yl)benzaldehyde (3d) (0.16 mmol, 50 mg), malononitrile (0.64 mmol, 42 mg), Al_2O_3 (0.16 mmol, 16 mg) and toluene (5 mL) were employed to afford 57 mg of product **4d** (99% yield) as a bright yellow solid; Eluent: hexane/ethylacetate (19/1); ^1H NMR (500 MHz, $CDCl_3$) δ 7.98 (d, J = 8.0 Hz, 2H), 77.78 (s, 1H), 7.69 (d, J = 7.8 Hz, 2H), 7.58 – 7.48 (m, 4H), 7.38 – 7.33 (m, 1H), 7.31 – 7.25 (m, 1H), 7.16 (d, J = 8.0 Hz, 2H), 2.37 (s, 3H); ^{13}C NMR (125 MHz, $CDCl_3$) δ 159.3, 154.3, 152.7, 140.5, 139.6, 131.6, 130.8, 129.9, 129.6, 129.0, 127.6, 127.1, 125.2, 123.5, 119.5, 115.3, 114.0, 112.9, 111.6, 82.2, 21.6; FT-IR: 3060, 3033, 2957, 2924, 2854, 2228, 1591, 1452, 747.1 cm^{-1}; LC-MS/MS calculated $C_{25}H_{16}N_2O$: 361.13354 $[M+H]^+$, found: 361.13257 $[M+H]^+$.

2-((5-(2-(p-Tolyl)benzofuran-3-yl)thiophen-2-yl)methylene)malononitrile (4e). 5-(2-(p-Tolyl)benzofuran-3-yl)thiophene-2-carbaldehyde (3e) (0.16 mmol, 50 mg), malononitrile (0.63 mmol, 41 mg), Al_2O_3 (0.16 mmol, 16 mg) and toluene (5 mL) were employed to afford 54 mg of product **4e** (93% yield) as an orange solid; Eluent: hexane/ethylacetate (19/1); ^1H NMR (500 MHz, $CDCl_3$) δ 7.83 – 7.76 (m, 2H), 7.71 (d, J = 7.7 Hz, 1H), 7.61 – 7.53 (m, 3H), 7.40 – 7.28 (m, 3H), 7.23 (d, J = 7.9 Hz, 2H), 2.40 (s, 3H); ^{13}C NMR (125 MHz, $CDCl_3$) δ 154.3, 154.2, 150.5, 147.4, 140.4, 139.0, 135.0, 129.7, 128.7, 128.3, 128.1, 126.6, 125.6, 124.0, 119.9, 114.3, 113.4, 111.6, 109.1, 77.0, 21.7; FT-IR: 2956, 2922, 2853, 2223, 1454, 810, 735, 663, 607, 436, 404 cm^{-1}; LC-MS/MS calculated $C_{23}H_{14}N_2OS$: 367.08996 $[M+H]^+$, found: 367.08951 $[M+H]^+$.

2-((5-(2-(p-Tolyl)benzofuran-3-yl)furan-2-yl)methylene)malononitrile (4f). 5-(2-(p-Tolyl)benzofuran-3-yl)furan-2-carbaldehyde (3f) (0.17 mmol, 50 mg), malononitrile (0.66 mmol, 44 mg), Al_2O_3 (0.17 mmol, 17 mg) and toluene (5 mL) were employed to afford 55 mg of product **4f** (95% yield) as an orange solid; Eluent: hexane/ethylacetate (19/1); ^1H NMR (500 MHz, $CDCl_3$) δ 8.14 – 8.08 (m, 1H), 7.67 (d, J = 8.2 Hz, 2H), 7.56 – 7.50 (m, 1H), 7.47 – 7.36 (m, 4H), 7.30 (d, J = 7.7 Hz, 2H), 6.80 (d, J = 3.8 Hz, 1H), 2.44 (s, 3H); ^{13}C NMR (125 MHz, $CDCl_3$) δ 156.9, 156.1, 154.3, 147.3, 141.5, 140.9, 129.7,

128.4, 126.9, 126.5, 126.1, 125.8, 124.2, 121.9, 114.7, 113.5, 112.4, 111.5, 106.6, 74.6, 21.7; FT-IR: 3024, 2923, 2220, 1578, 1509, 1327, 1198, 1083, 796, 744, 598, 499 cm^{-1}; LC-MS/MS calculated $C_{23}H_{14}N_2O_2$: 351.11280 $[M+H]^+$, found: 351.11279 $[M+H]^+$.

2-(4-(2-(Naphthalen-1-yl)benzofuran-3-yl)benzylidene)malononitrile ***(4g)***. 4-(2-(Naphthalen-1-yl)benzofuran-3-yl)benzaldehyde (3g) (0.14 mmol, 50 mg), malononitrile (0.57 mmol, 38 mg), Al_2O_3 (0.14 mmol, 15 mg) and toluene (5 mL) were employed to afford 55 mg of product **4g** (96% yield) as bright yellow solid; Eluent: hexane/ethylacetate (19/1); ^{1}H NMR (500 MHz, $CDCl_3$) δ 7.98 – 7.87 (m, 3H), 7.83 – 7.78 (m, 3H), 7.67 – 7.62 (m, 2H), 7.55 – 7.49 (m, 4H), 7.48 – 7.37 (m, 4H); ^{13}C NMR (125 MHz, $CDCl_3$) δ 159.2, 155.0, 153.3, 139.9, 134.0, 131.7, 131.3, 130.7, 129.9, 129.7, 129.5, 128.7, 127.6, 127.5, 127.1, 126.6, 125.7, 125.4, 125.3, 123.8, 120.0, 118.4, 114.0, 112.9, 112.0, 81.8; FT-IR: 2959, 2924, 2853, 2227, 1582, 1544, 1452, 1262, 1104, 1024, 802, 777, 748 cm^{-1}; LC-MS/MS calculated $C_{28}H_{16}N_2O$: 397.13354 $[M+H]^+$, found: 397.13330 $[M+H]^+$.

2-((5-(2-(Naphthalen-1-yl)benzofuran-3-yl)thiophen-2-yl)methylene)malononitrile ***(4h)***. 5-(2-(Naphthalen-1-yl)benzofuran-3-yl)thiophene-2-carbaldehyde (3h) (0.14 mmol, 50 mg), malononitrile (0.56 mmol, 37 mg), Al_2O_3 (0.14 mmol, 14 mg) and toluene (5 mL) were employed to afford 53 mg of product **4h** (93% yield) as an orange solid; Eluent: hexane/ethylacetate (19/1); ^{1}H NMR (500 MHz, $CDCl_3$) δ 8.08 – 7.93 (m, 3H), 7.74 – 7.69 (m, 2H), 7.65 – 7.57 (m, 3H), 7.56 – 7.51 (m, 2H), 7.49 – 7.41 (m, 3H), 6.96 (d, J = 4.2 Hz, 1H); ^{13}C NMR (125 MHz, $CDCl_3$) δ 154.9, 154.2, 150.2, 146.9, 138.6, 134.6, 133.9, 131.6, 131.3, 129.8, 128.8, 127.4, 127.2, 127.1, 126.8, 126.5, 125.9, 125.4, 125.2, 124.3, 120.5, 114.3, 113.7, 113.0, 112.0, 76.4; FT-IR: 2925, 2222, 1558, 1443, 1261, 1104, 910, 745, 608, 502 cm^{-1}; LC-MS/MS calculated $C_{26}H_{14}N_2OS$: 403.08996 $[M+H]^+$, found: 403.08643 $[M+H]^+$.

2-((5-(2-(Naphthalen-1-yl)benzofuran-3-yl)furan-2-yl)methylene)malononitrile ***(4i)***. 5-(2-(Naphthalen-1-yl)benzofuran-3-yl)furan-2-carbaldehyde (3i) (0.15 mmol, 50 mg), malononitrile (0.59 mmol, 39 mg), Al_2O_3 (0.15 mmol, 15 mg) and toluene (5 mL) were employed to afford 52 mg of product **4i** (91% yield) as an orange solid; Eluent: hexane/ethylacetate (19/1); ^{1}H NMR (500 MHz, $CDCl_3$) δ 8.44 (s, 1H), 8.07 (d, J = 8.2 Hz, 1H), 7.97 (d, J = 8.2 Hz, 1H), 7.76 – 7.70 (m, 2H), 7.65 – 7.54 (m, 3H), 7.51 – 7.44 (m, 3H), 7.33 (s, 1H), 7.13 (s, 1H), 6.02 (s, 1H); ^{13}C NMR (125 MHz, $CDCl_3$) δ 156.7, 155.2, 154.8, 147.2, 141.3, 133.9, 131.4 (2C), 129.4, 128.8, 127.6, 127.4, 126.9, 126.1, 125.4, 125.3 (2C), 124.5, 122.7 (2C), 114.7, 113.7, 112.0, 111.6, 110.1, 74.3; FT-IR: 3050, 2227, 1572, 1383, 1265, 1143, 1036, 765 cm^{-1}; LC-MS/MS calculated $C_{26}H_{14}N_2O_2$: 387.11280 $[M+H]^+$, found: 387.10947 $[M+H]^+$.

2-(4-(2-Pentylbenzofuran-3-yl)benzylidene)malononitrile ***(4j)***. 4-(2-Pentylbenzofuran-3-yl)benzaldehyde (3j) (0.17 mmol, 50 mg), malononitrile (0.68 mmol, 45 mg), Al_2O_3 (0.17 mmol, 17 mg) and toluene (5 mL) were employed to afford 57 mg of product **4j** (98% yield) as a bright yellow solid; Eluent: hexane/ethylacetate (19/1); ^{1}H NMR (500 MHz, $CDCl_3$) δ 8.03 (d, J = 8.5 Hz, 2H), 7.79 (s, 1H),

7.67 (d, *J* = 8.4 Hz, 2H), 7.58 – 7.54 (m, 1H), 7.50 – 7.47 (m, 1H), 7.33 – 7.25 (m, 2H), 2.88 (t, *J* = 7.6 Hz, 2H), 1.84 – 1.76 (m, 2H), 1.37 – 1.30 (m, 4H), 0.88 (t, *J* = 6.9 Hz, 3H); ^{13}C NMR (125 MHz, $CDCl_3$) δ 159.3, 157.2, 154.2, 140.3, 131.5, 129.9, 129.5, 127.8, 124.3, 123.2, 119.2, 115.8, 114.0, 113.0, 111.3, 82.0, 31.6, 28.1, 27.2, 22.5, 14.0; FT-IR: 3032, 2950, 2926, 2858, 2228, 1605, 1576, 1547, 1453, 1172, 842, 745, 613 cm^{-1}; LC-MS/MS calculated $C_{23}H_{20}N_2O$: 341.16484 [M+H]$^+$, found: 341.16351 [M+H]$^+$.

2-((5-(2-Pentylbenzofuran-3-yl)thiophen-2-yl)methylene)malononitrile (4k). 5-(2-Pentylbenzofuran-3-yl)thiophene-2-carbaldehyde (3k) (0.17 mmol, 50 mg), malononitrile (0.67 mmol, 44 mg), Al_2O_3 (0.17 mmol, 17 mg) and toluene (5 mL) were employed to afford 54 mg of product **4k** (93% yield) as an orange solid; Eluent: hexane/ethylacetate (19/1); ^{1}H NMR (500 MHz, $CDCl_3$) δ 7.83 – 7.78 (m, 2H), 7.76 – 7.72 (m, 1H), 7.50 – 7.47 (m, 1H), 7.38 (d, *J* = 4.1 Hz, 1H), 7.35 – 7.29 (m, 2H), 3.01 (t, *J* = 7.7 Hz, 2H), 1.87 – 1.78 (m, 2H), 1.42 – 1.32 (m, 4H), 0.89 (t, *J* = 7.1 Hz, 3H); ^{13}C NMR (125 MHz, $CDCl_3$) δ 159.1, 154.1, 150.5, 147.5, 139.5, 133.9, 126.8, 126.6, 124.9, 123.8, 119.6, 114.4, 113.5, 111.4, 110.1, 76.4, 31.6, 27.9, 27.8, 22.5, 14.1; FT-IR: 2958, 2873, 2218, 1555, 1447, 1187, 1066, 942, 741, 606 cm^{-1}; LC-MS/MS calculated $C_{21}H_{18}N_2OS$: 347.12126 [M+H]$^+$, found: 347.12137 [M+H]$^+$.

2-((5-(2-Pentylbenzofuran-3-yl)furan-2-yl)methylene)malononitrile (4l). 5-(2-Pentylbenzofuran-3-yl)furan-2-carbaldehyde (3l) (0.18 mmol, 50 mg), malononitrile (0.71 mmol, 47 mg), Al_2O_3 (0.18 mmol, 18 mg) and toluene (5 mL) were employed to afford 56 mg of product **4l** (96% yield) as an orange solid; Eluent: hexane/ethylacetate (19/1); ^{1}H NMR (500 MHz, $CDCl_3$) δ 7.92 – 7.87 (m, 1H), 7.54 – 7.40 (m, 3H), 7.35 – 7.31 (m, 2H), 6.89 (d, *J* = 3.9 Hz, 1H), 3.15 (t, *J* = 7.6 Hz, 2H), 1.84 – 1.77 (m, 2H), 1.38 – 1.32 (m, 4H), 0.88 (t, *J* = 7.2 Hz, 3H); ^{13}C NMR (125 MHz, $CDCl_3$) δ 161.1, 154.1, 146.9, 141.2, 131.0, 128.9, 125.5, 124.9, 123.9, 120.5, 114.8, 113.5, 111.4, 111.2, 106.7, 74.3, 31.4, 28.5, 27.6, 22.5, 14.1; FT-IR: 2927, 2222, 1569, 1337, 1202, 1049, 925, 743, 600 cm^{-1}; LC-MS/MS calculated $C_{21}H_{18}N_2O_2$: 331.14410 [M+H]$^+$, found: 331.14401 [M+H]$^+$.

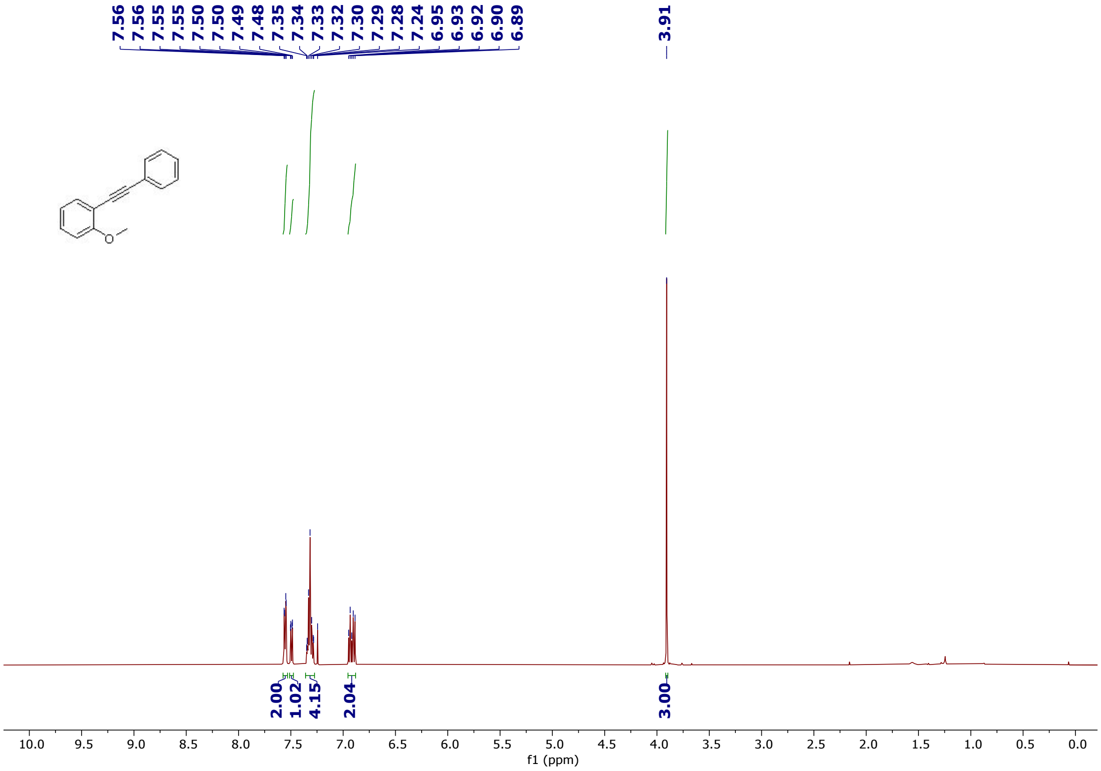

Figure S1: ^{1}H NMR spectrum of 1a

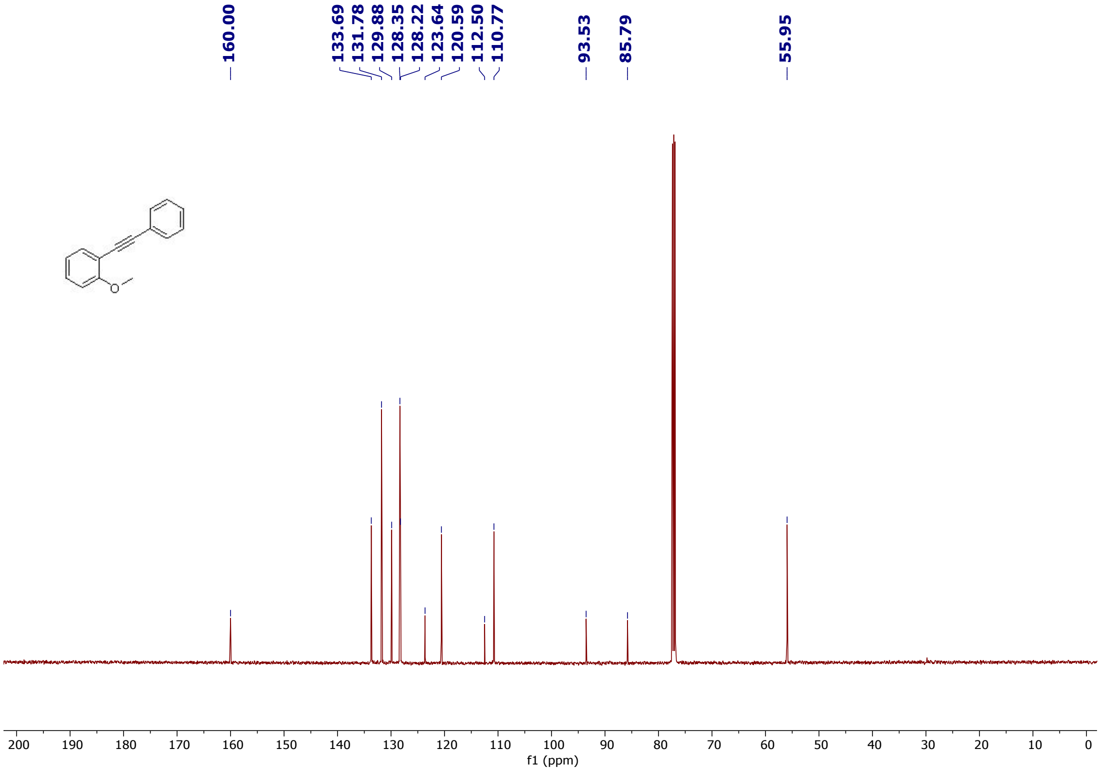

Figure S2: ^{13}C NMR spectrum of 1a

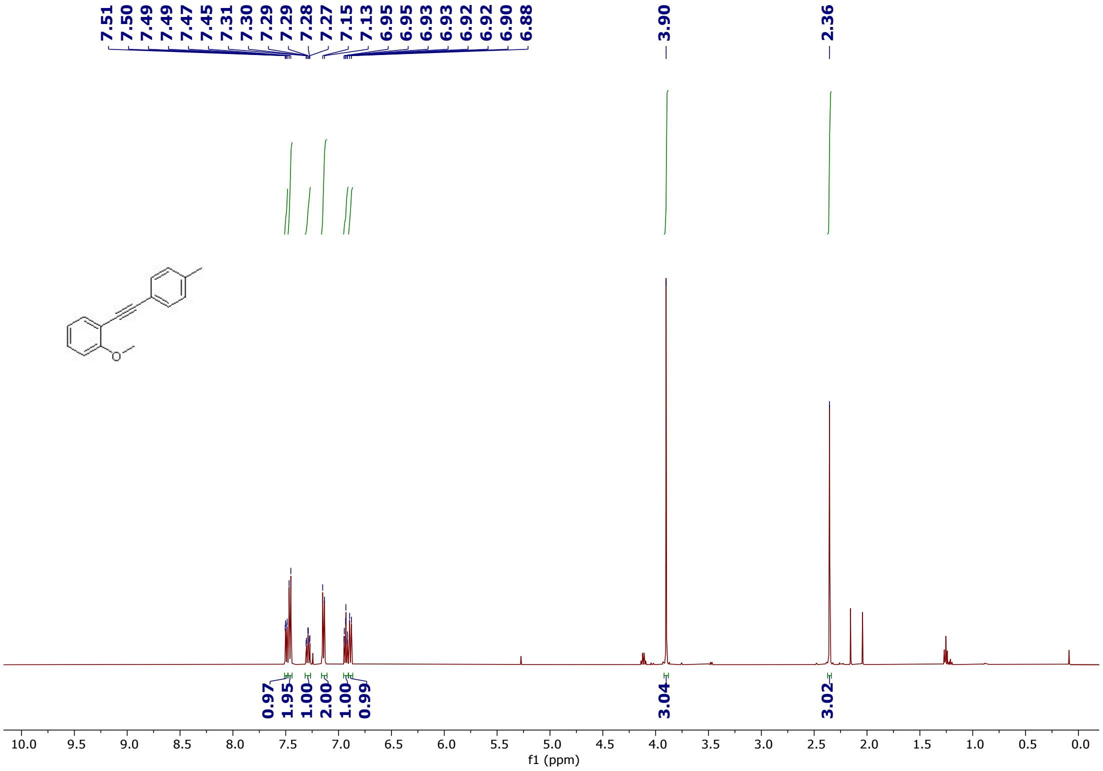

Figure S3: ^{1}H NMR spectrum of 1b

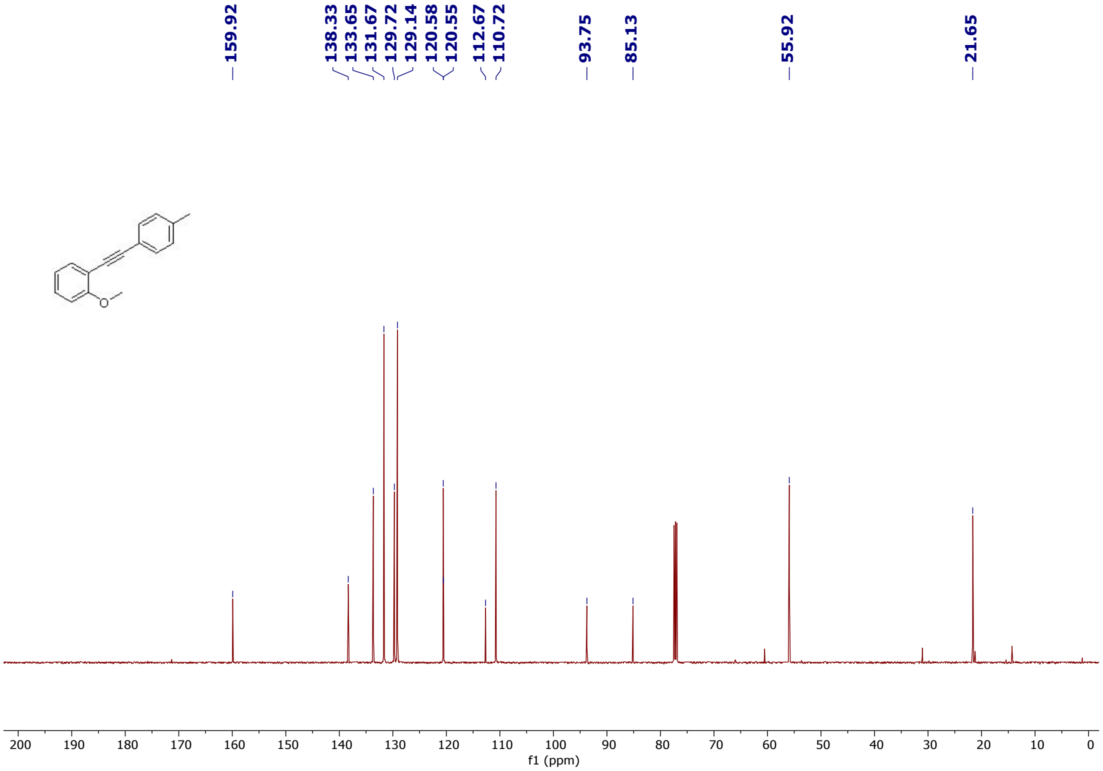

Figure S4: ^{13}C NMR spectrum of 1b

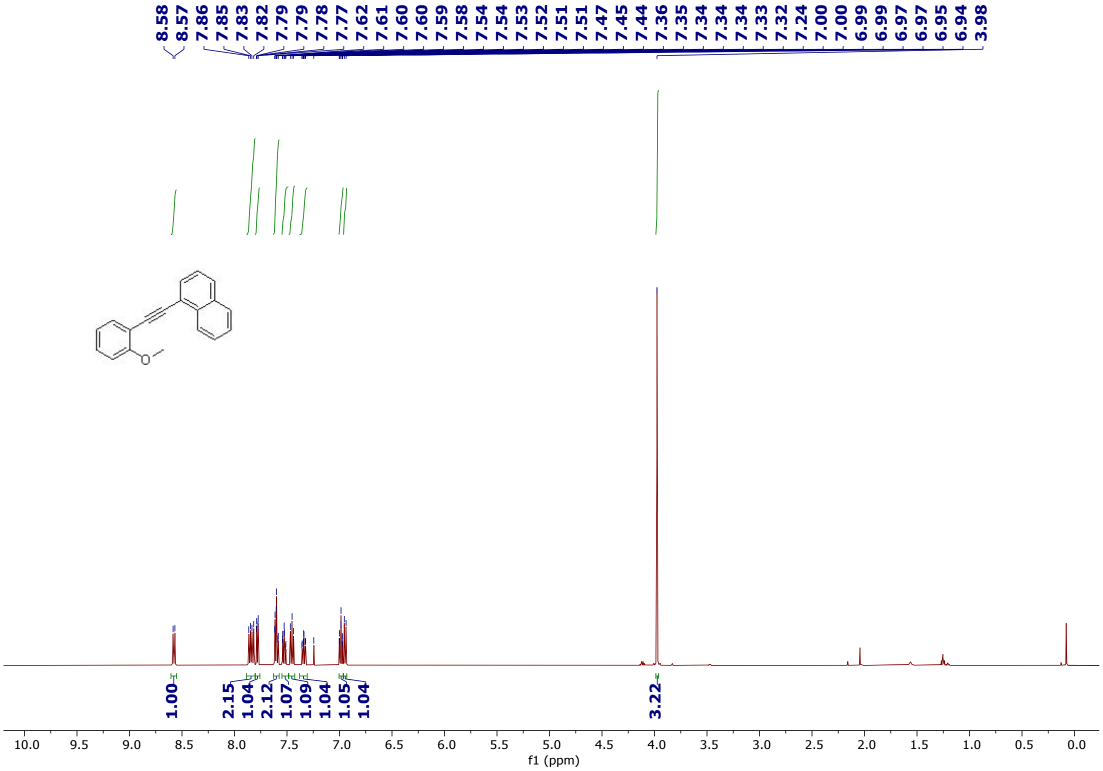

Figure S5: ^{1}H NMR spectrum of 1c

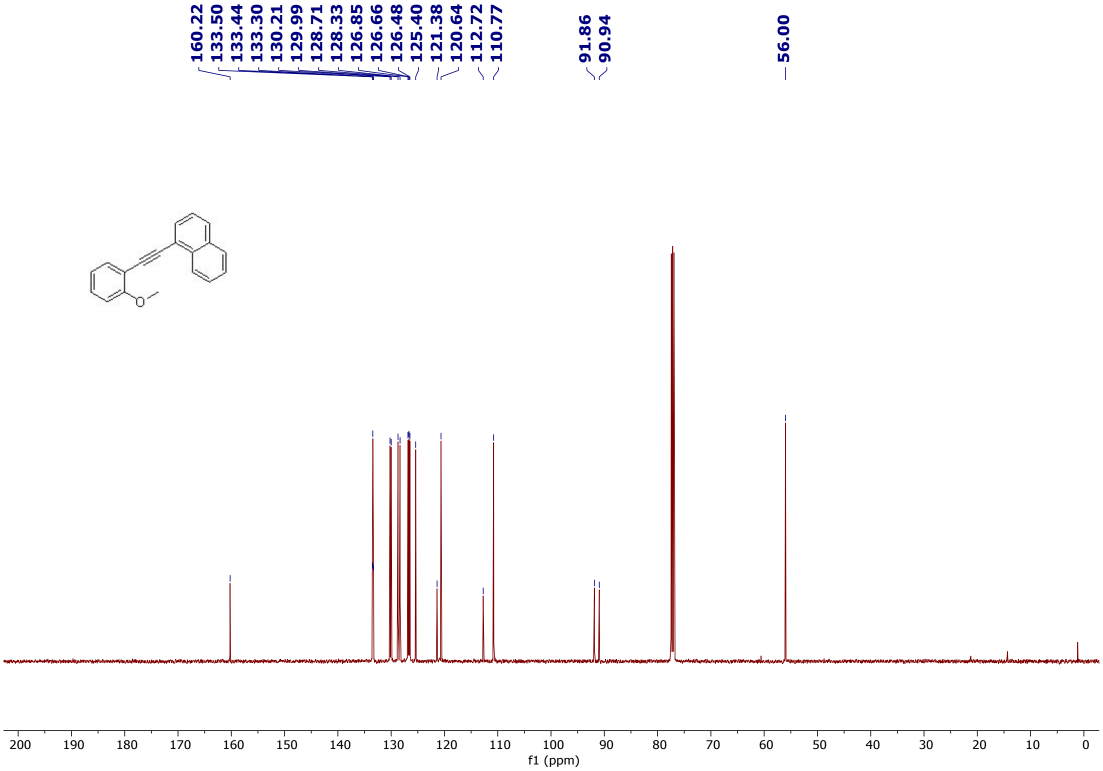

Figure S6: ^{13}C NMR spectrum of 1c

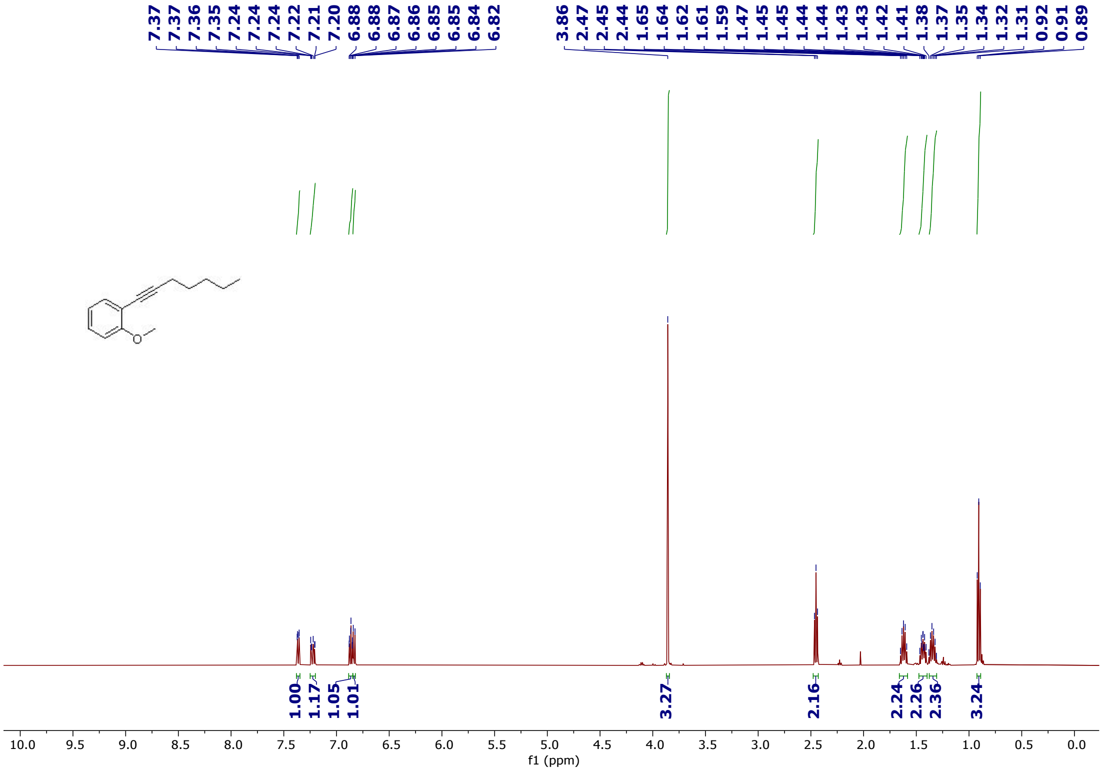

Figure S7: ^{1}H NMR spectrum of 1d

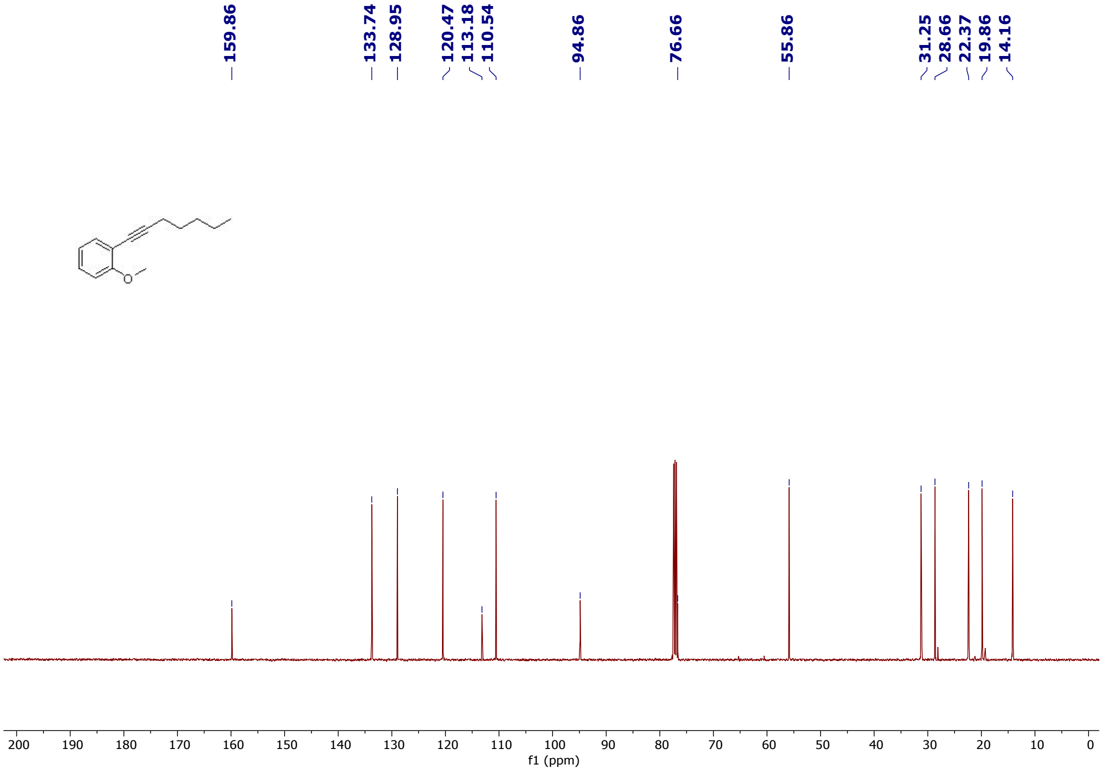

Figure S8: ^{13}C NMR spectrum of 1d

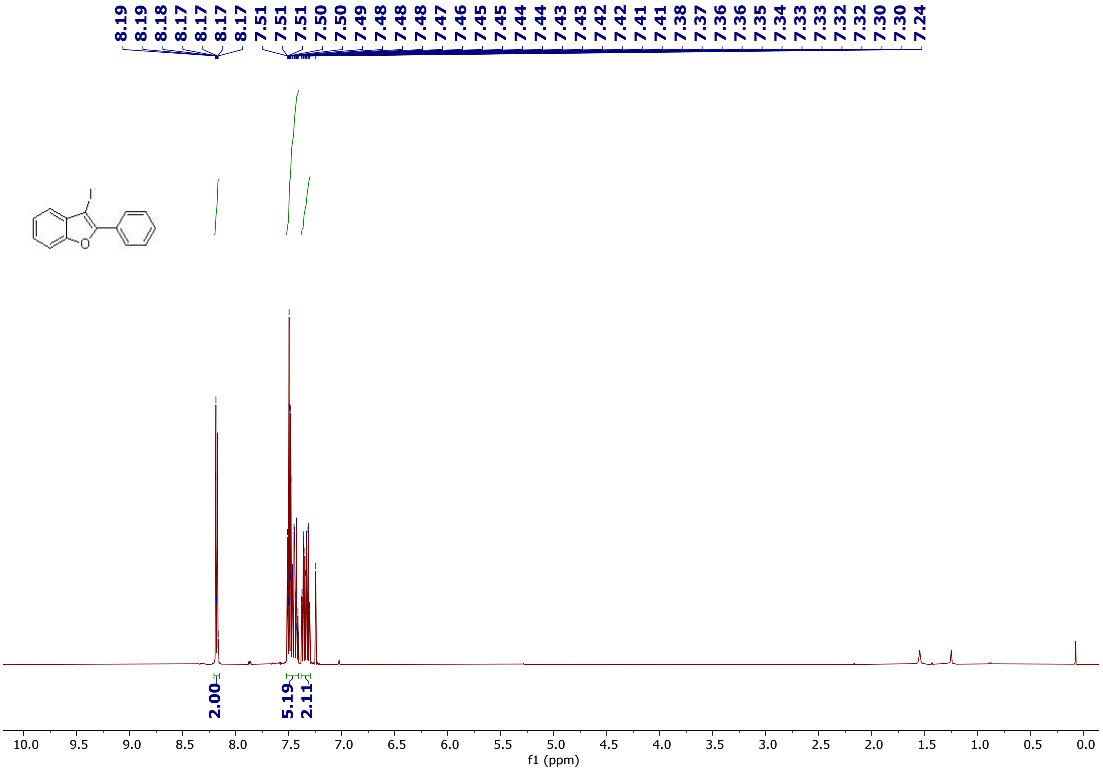

Figure S9: ^{1}H NMR spectrum of 2a

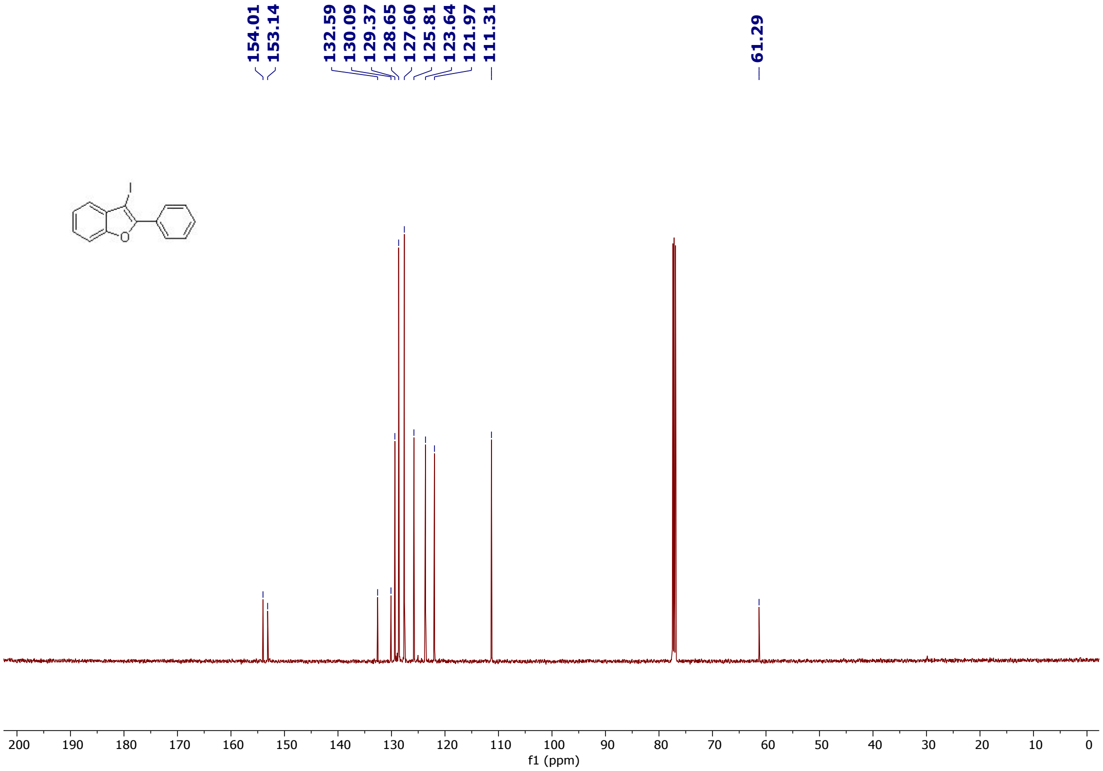

Figure S10: ^{13}C NMR spectrum of 2a

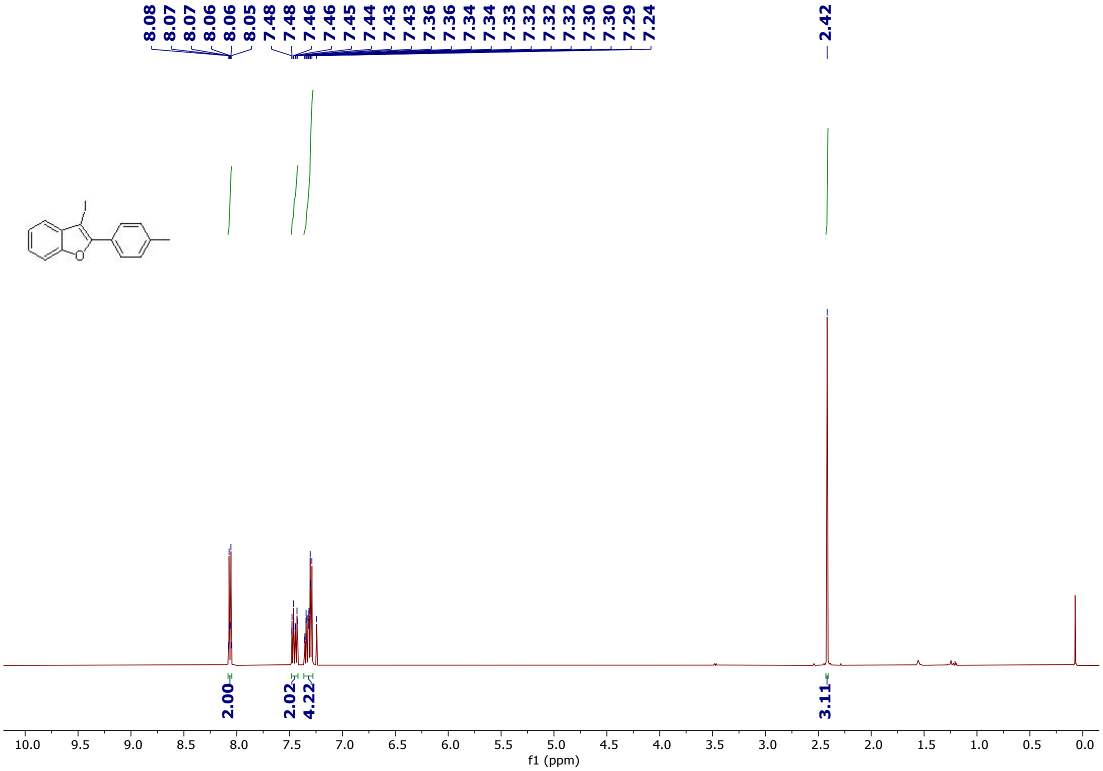

Figure S11: ^{1}H NMR spectrum of 2b

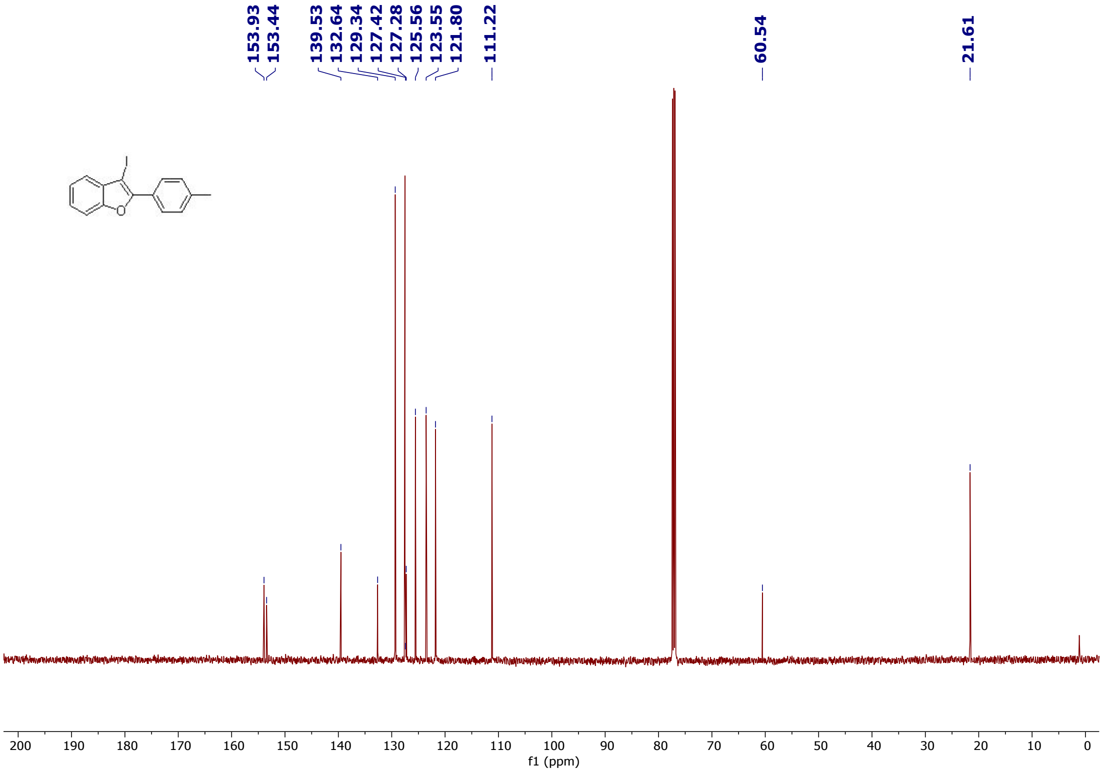

Figure S12: ^{13}C NMR spectrum of 2b

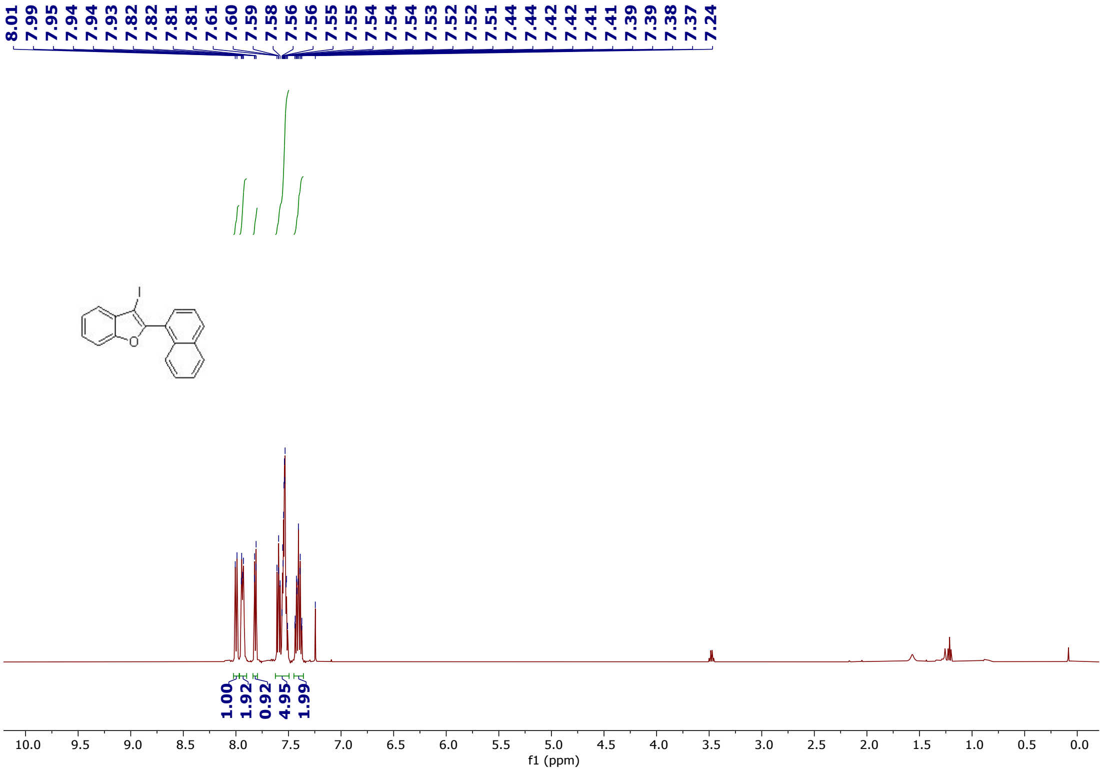

Figure S13: ^{1}H NMR spectrum of 2c

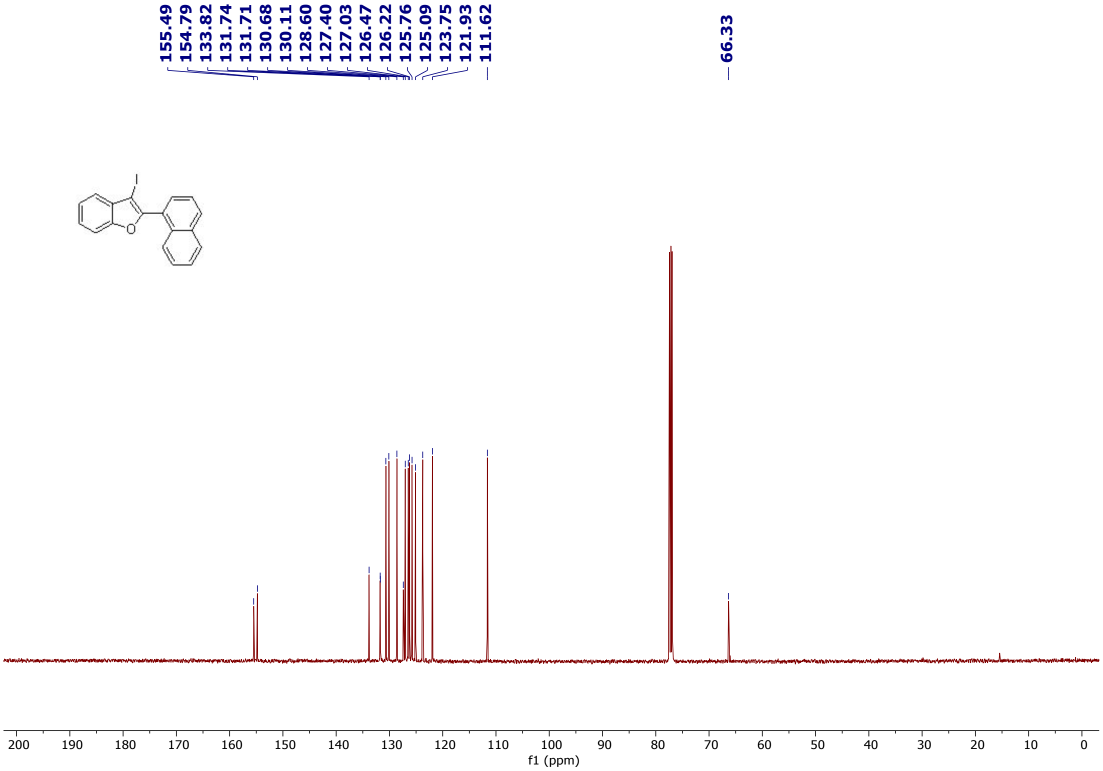

Figure S14: ^{13}C NMR spectrum of 2c

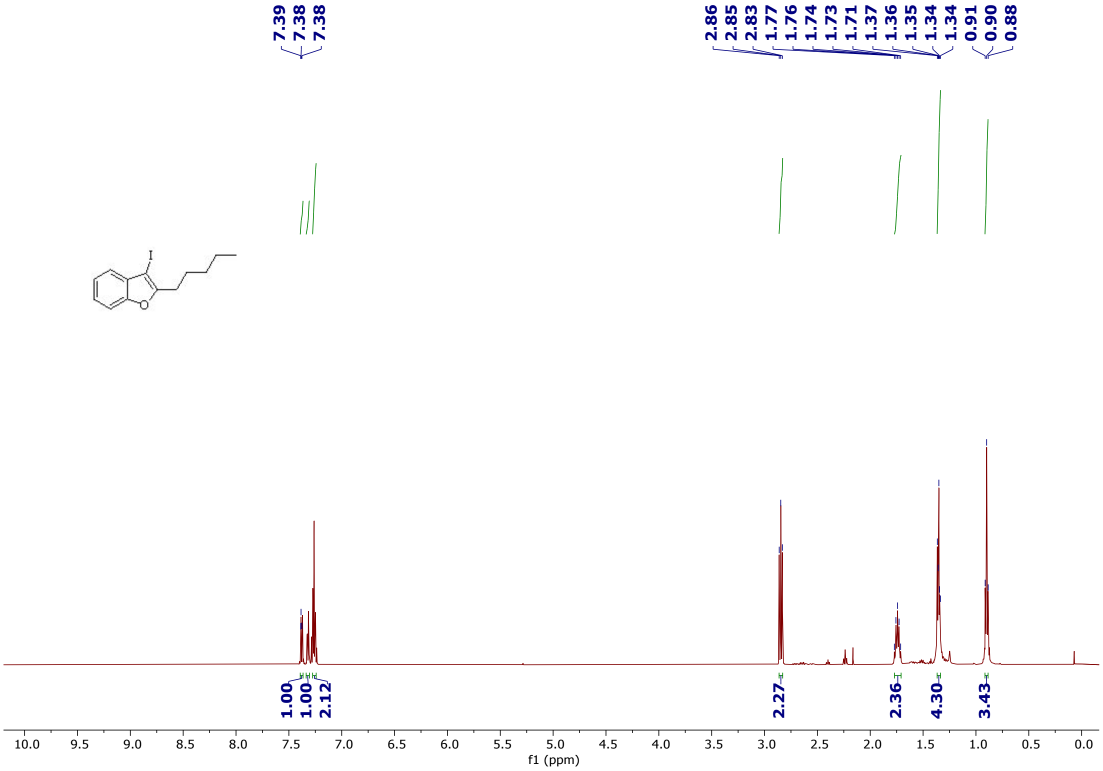

Figure S15: ^{1}H NMR spectrum of 2d

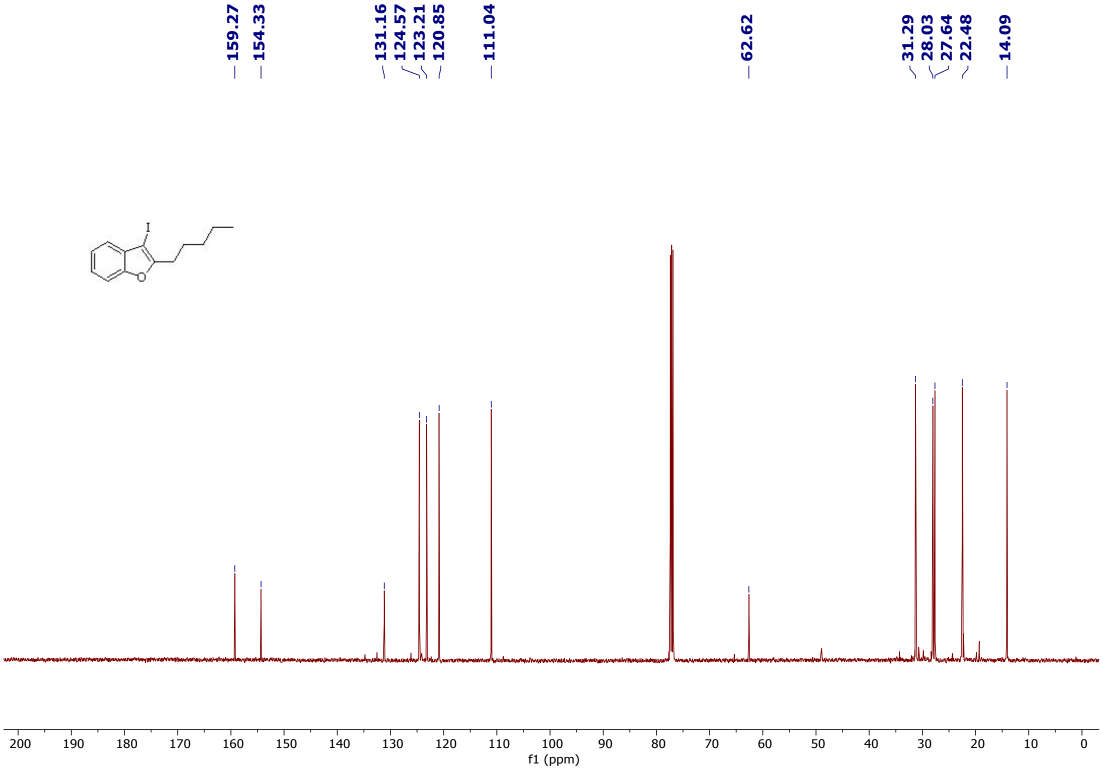

Figure S16: ^{13}C NMR spectrum of 2d

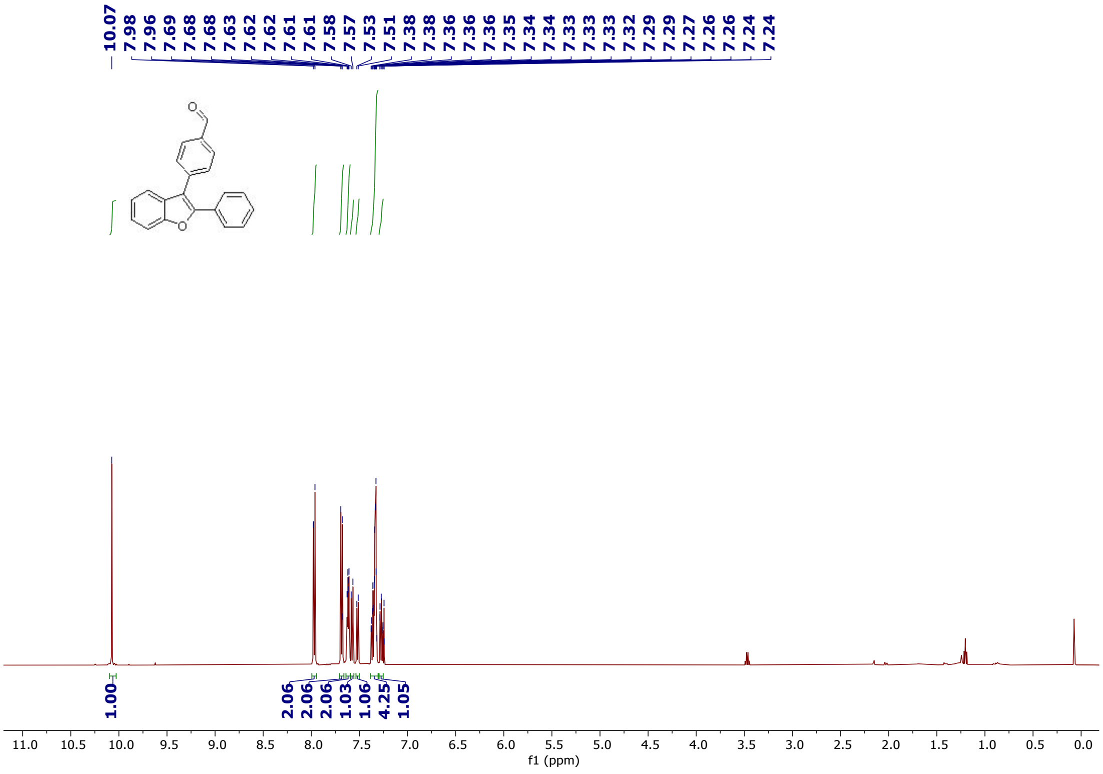

Figure S17: ^{1}H NMR spectrum of 3a

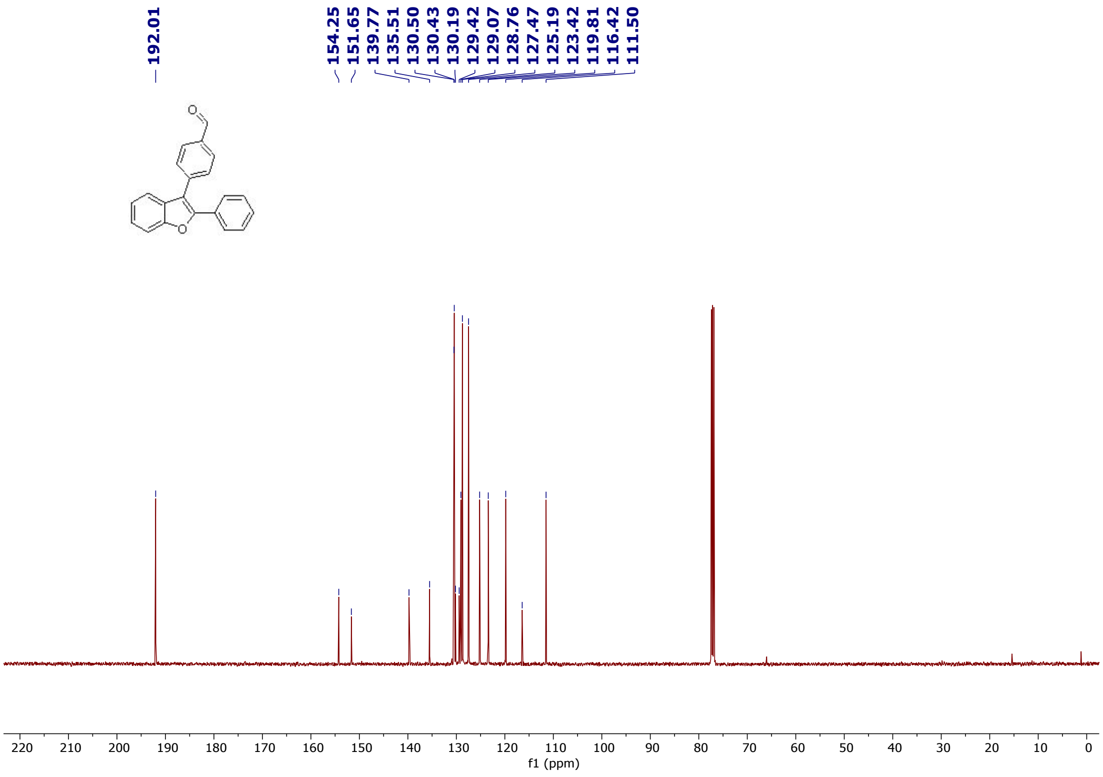

Figure S18: ^{13}C NMR spectrum of 3a

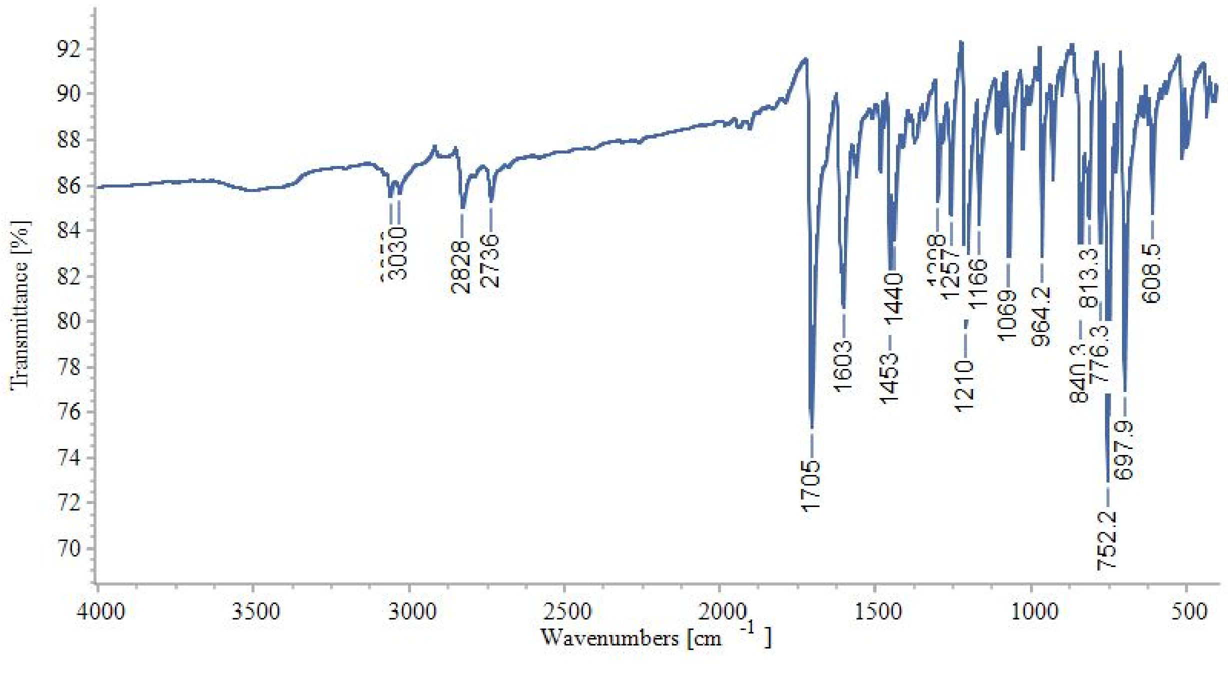

Figure S19: FT-IR spectrum of 3a

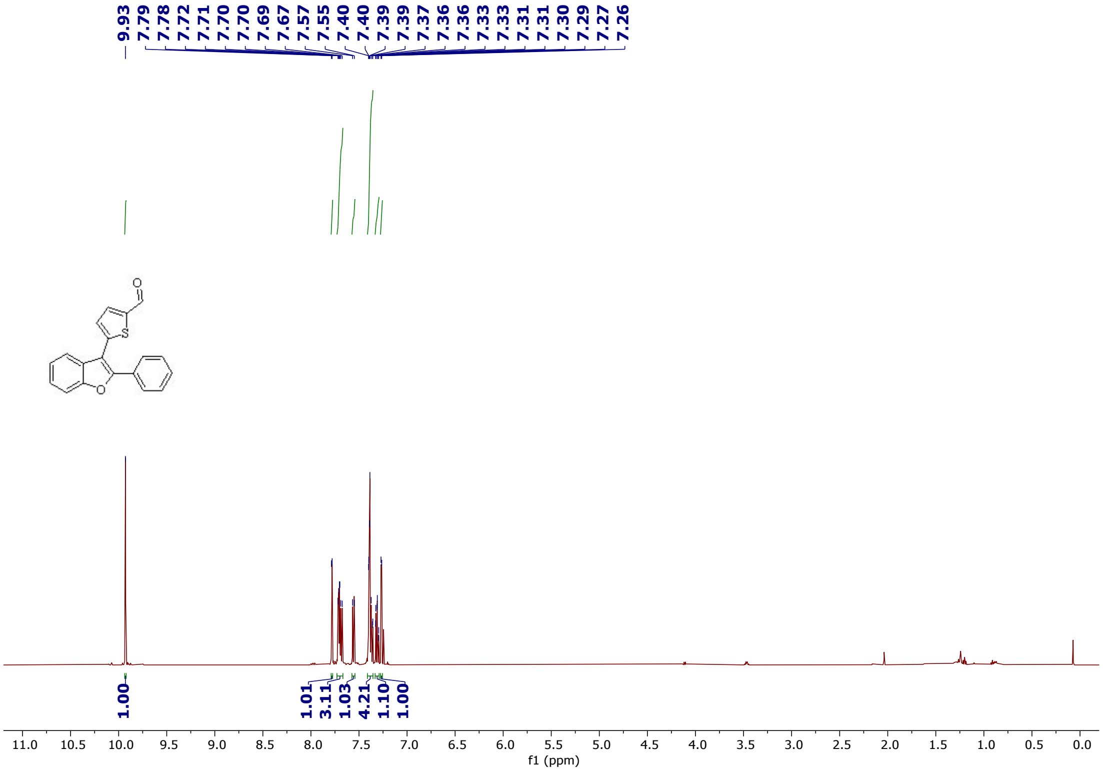

Figure S20: ^{1}H NMR spectrum of 3b

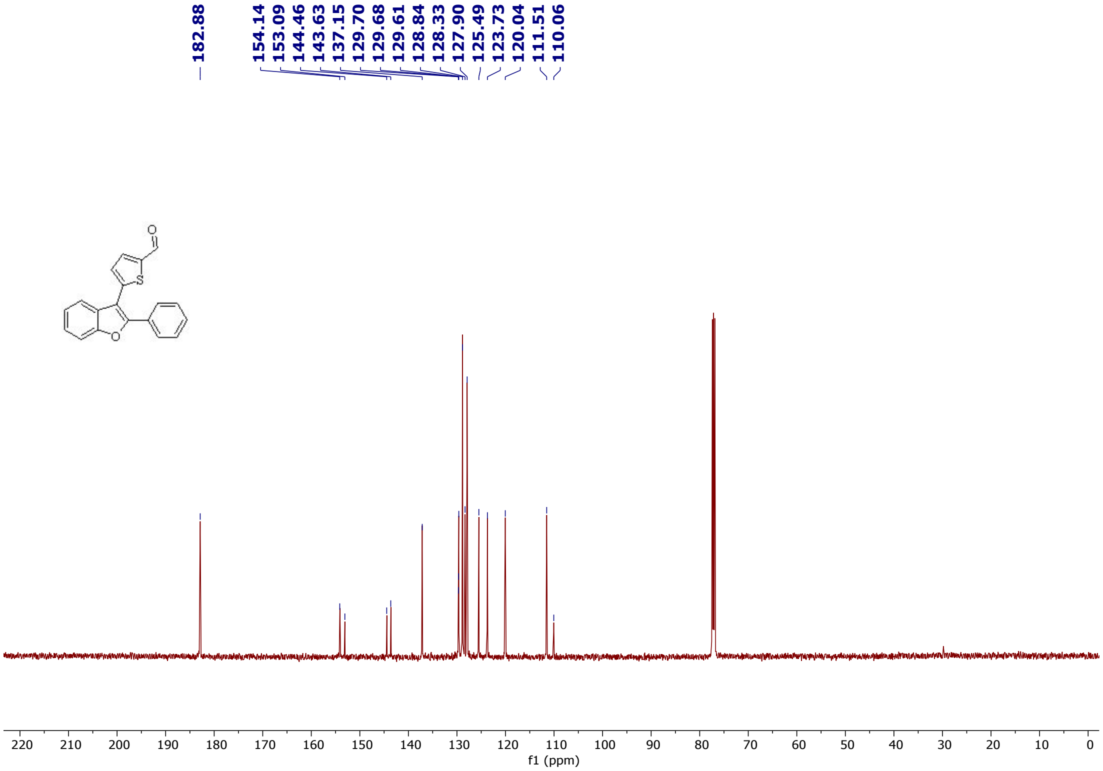

Figure S21: ^{13}C NMR spectrum of 3b

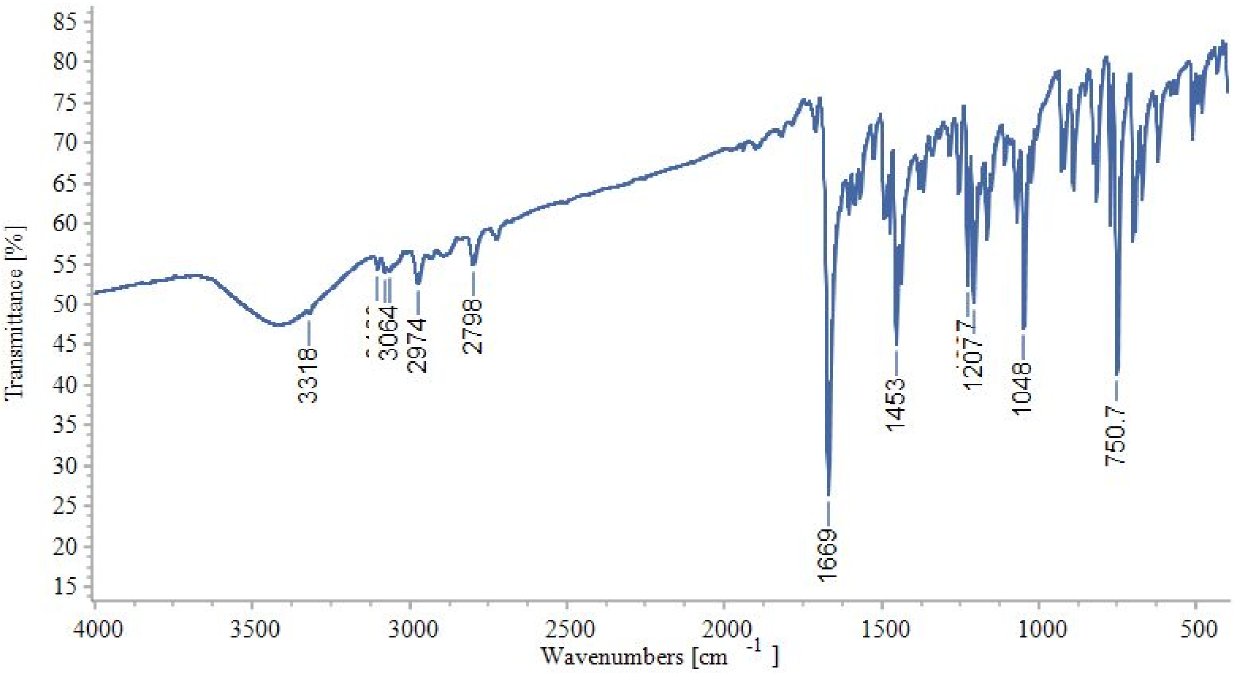

Figure S22: FT-IR spectrum of 3b

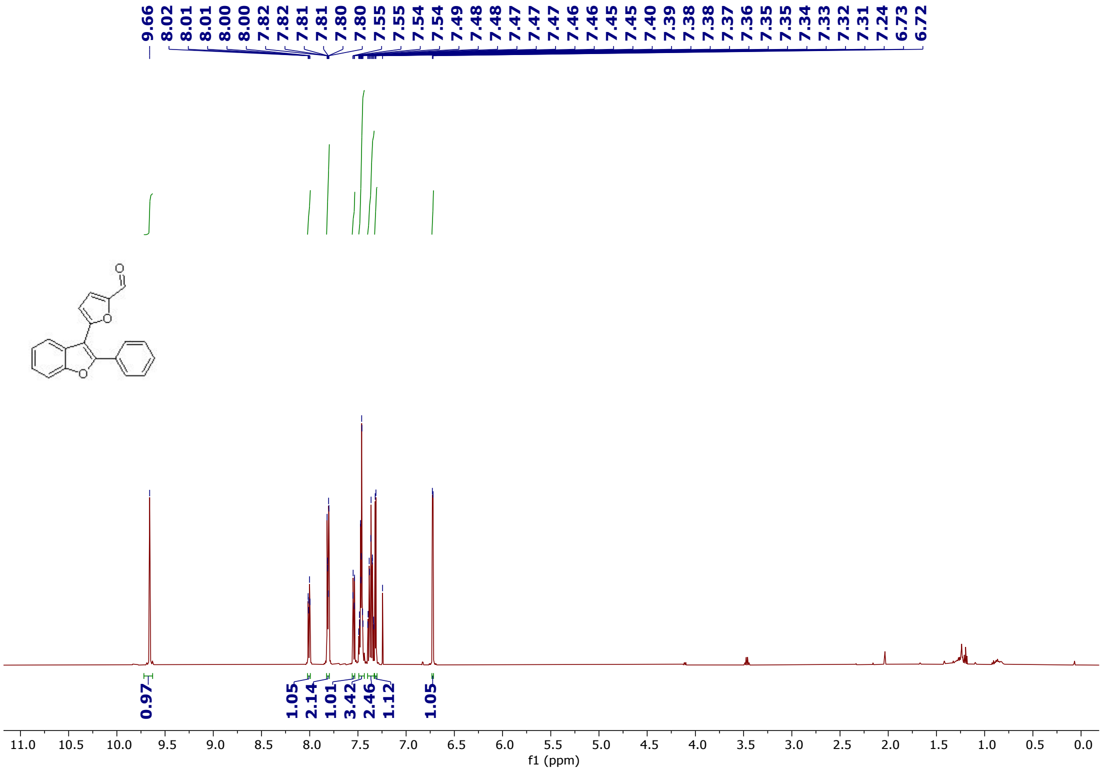

Figure S23: ^{1}H NMR spectrum of 3c

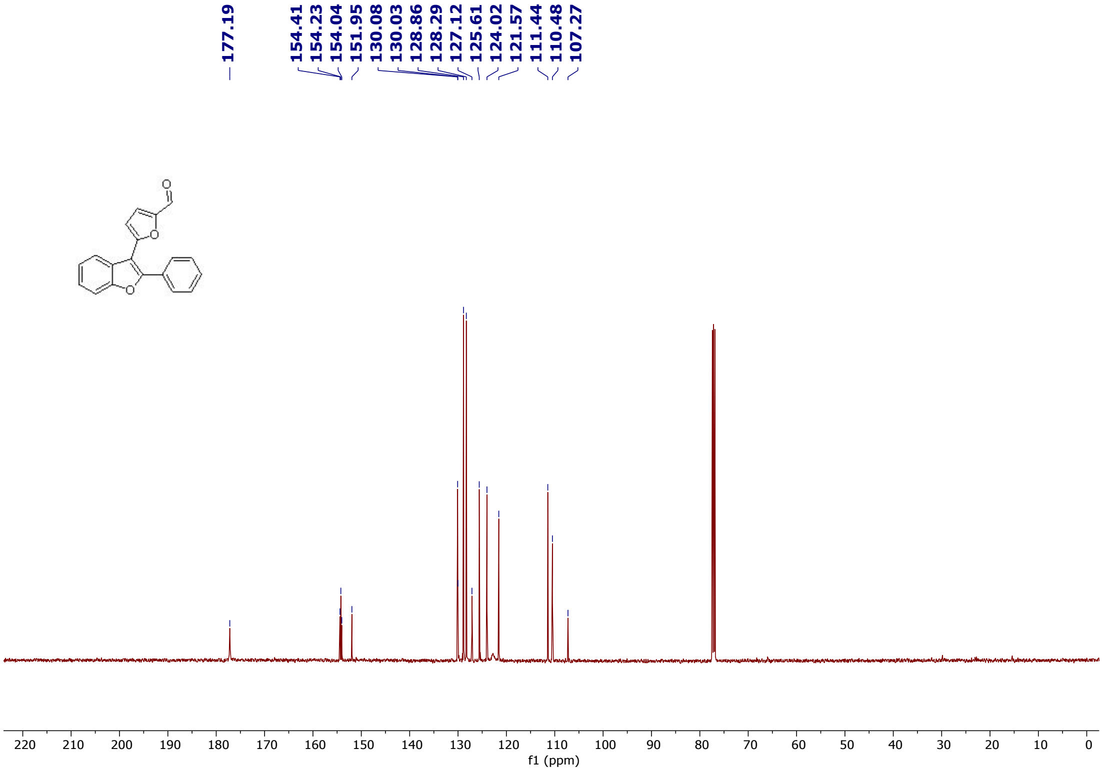

Figure S24: ^{13}C NMR spectrum of 3c

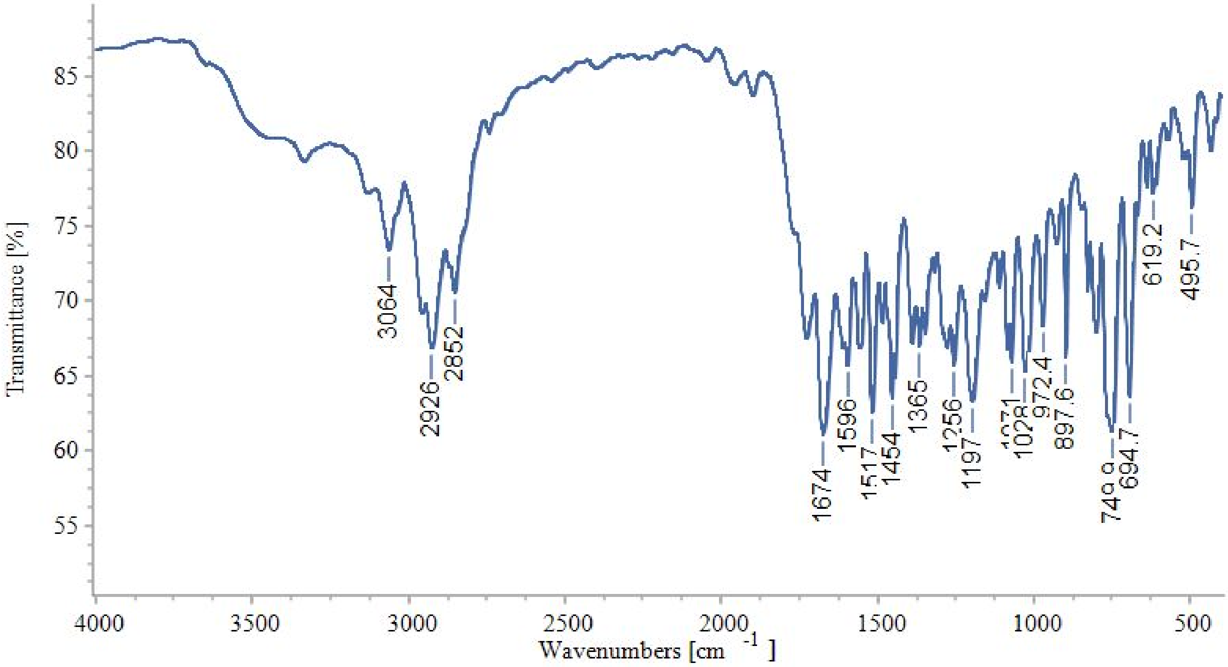

Figure S25: FT-IR spectrum of 3c

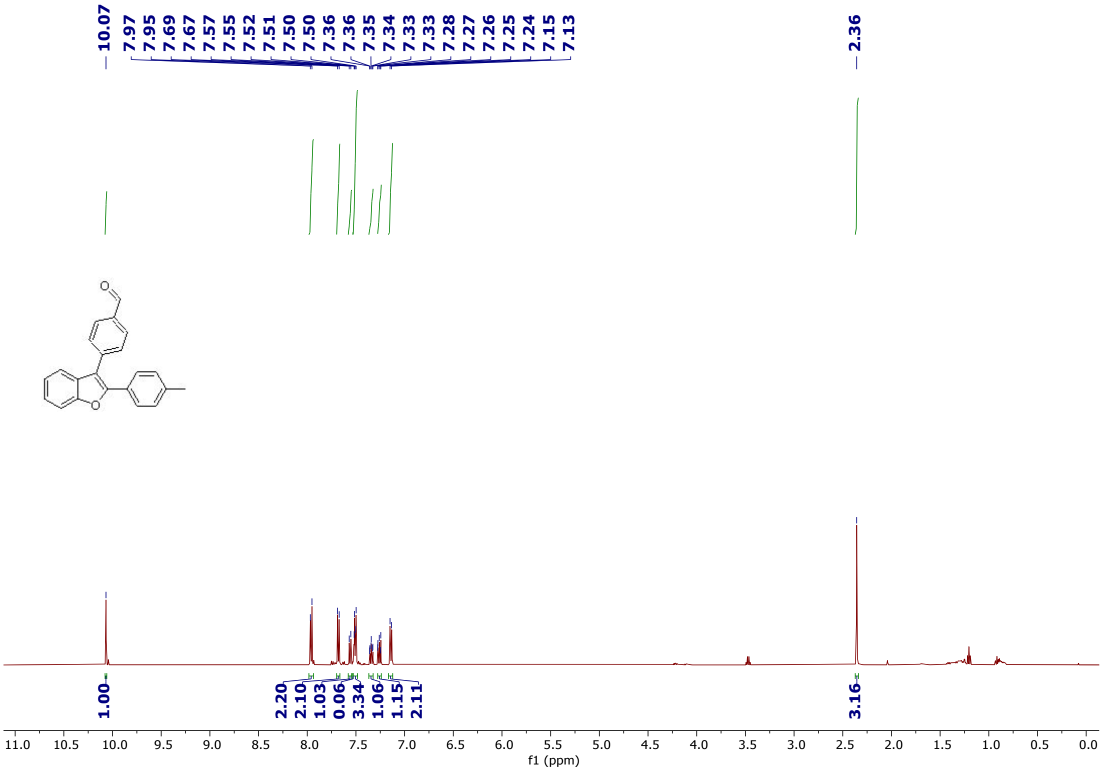

Figure S26: ^{1}H NMR spectrum of 3d

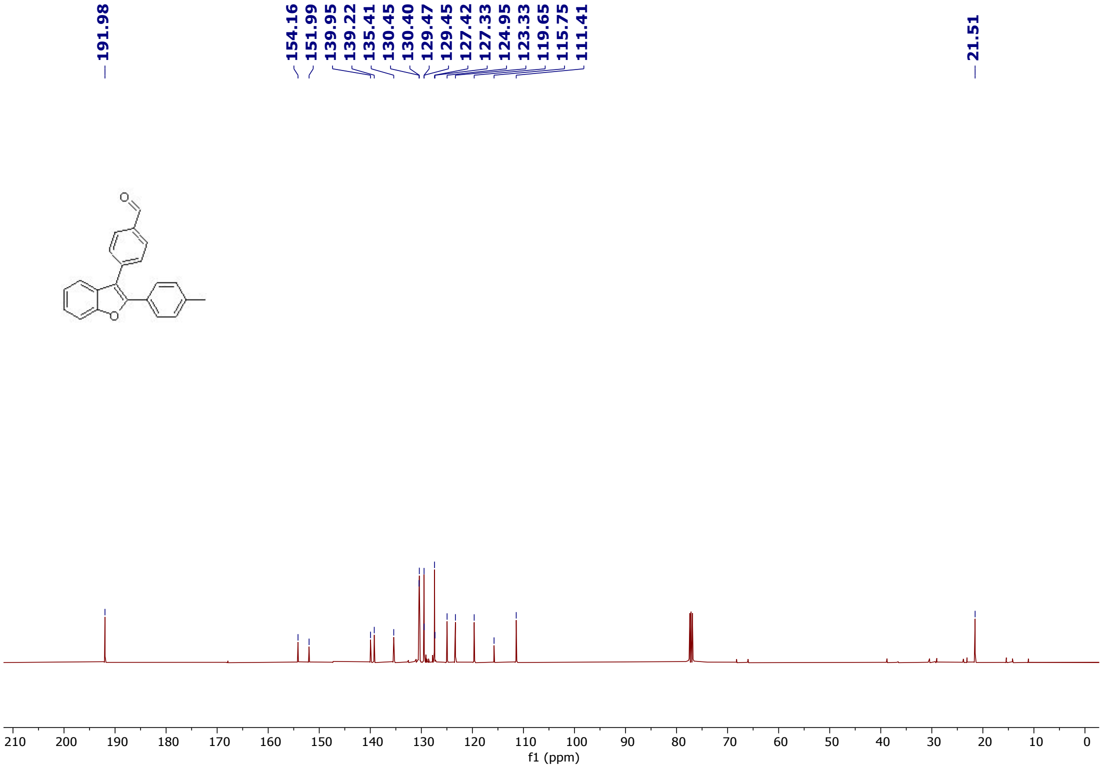

Figure S27: ^{13}C NMR spectrum of 3d

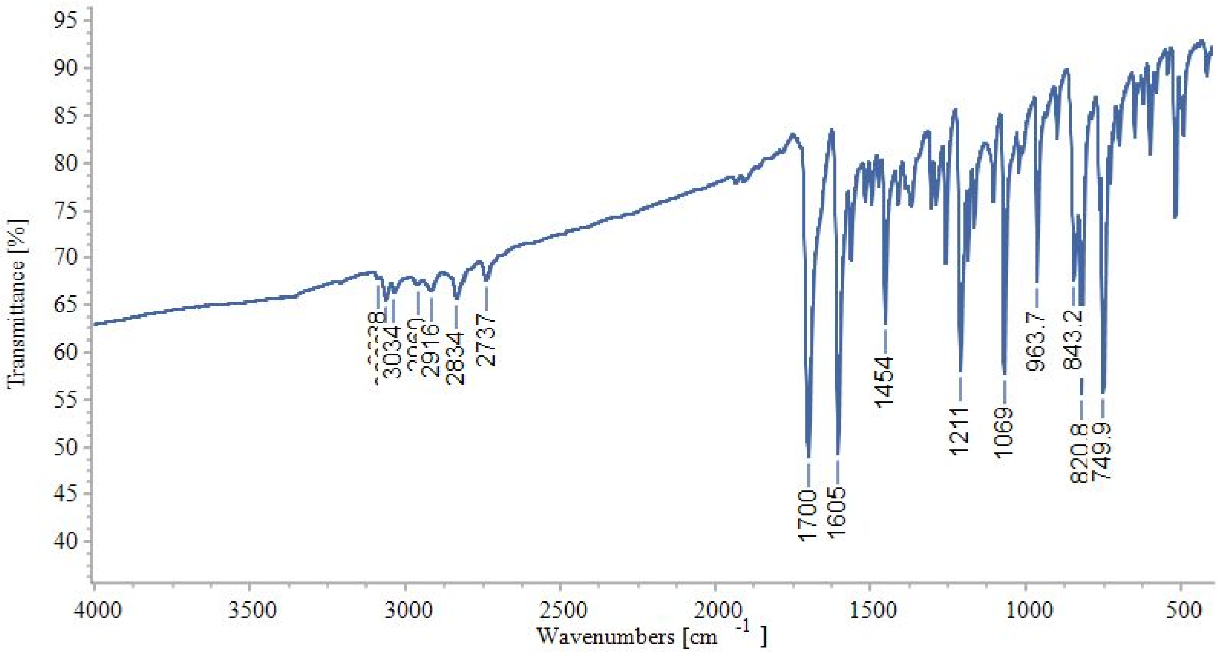

Figure S28: FT-IR spectrum of 3d

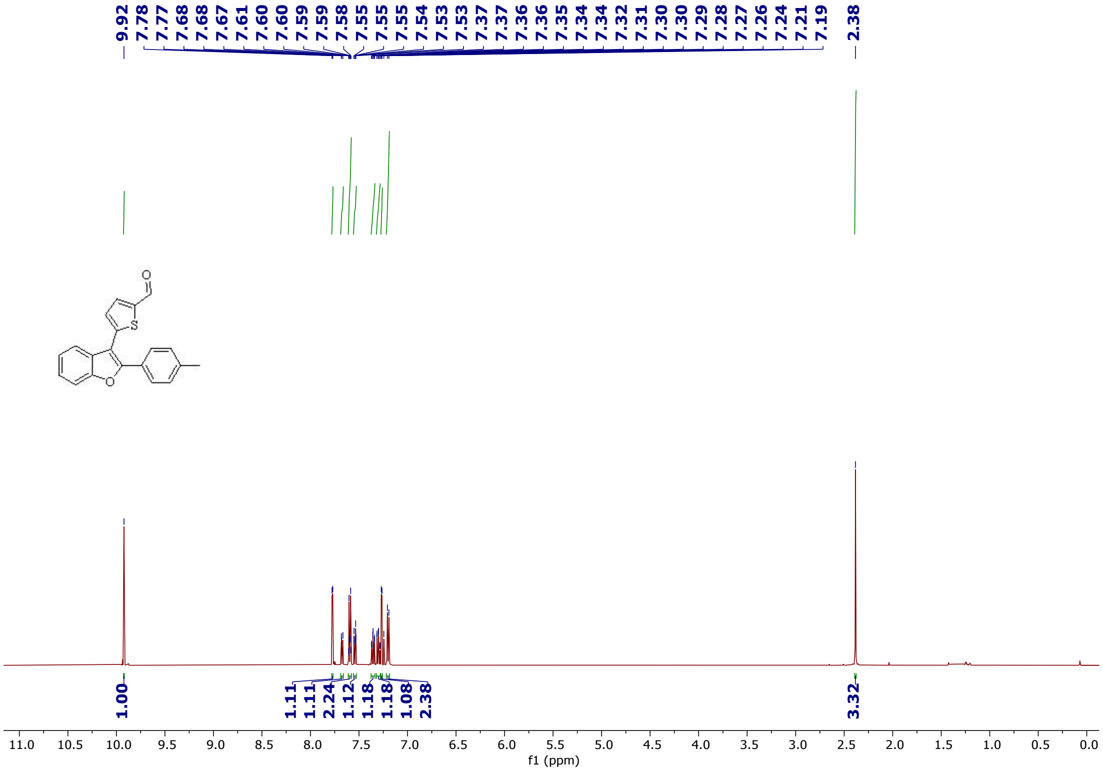

Figure S29: ^{1}H NMR spectrum of 3e

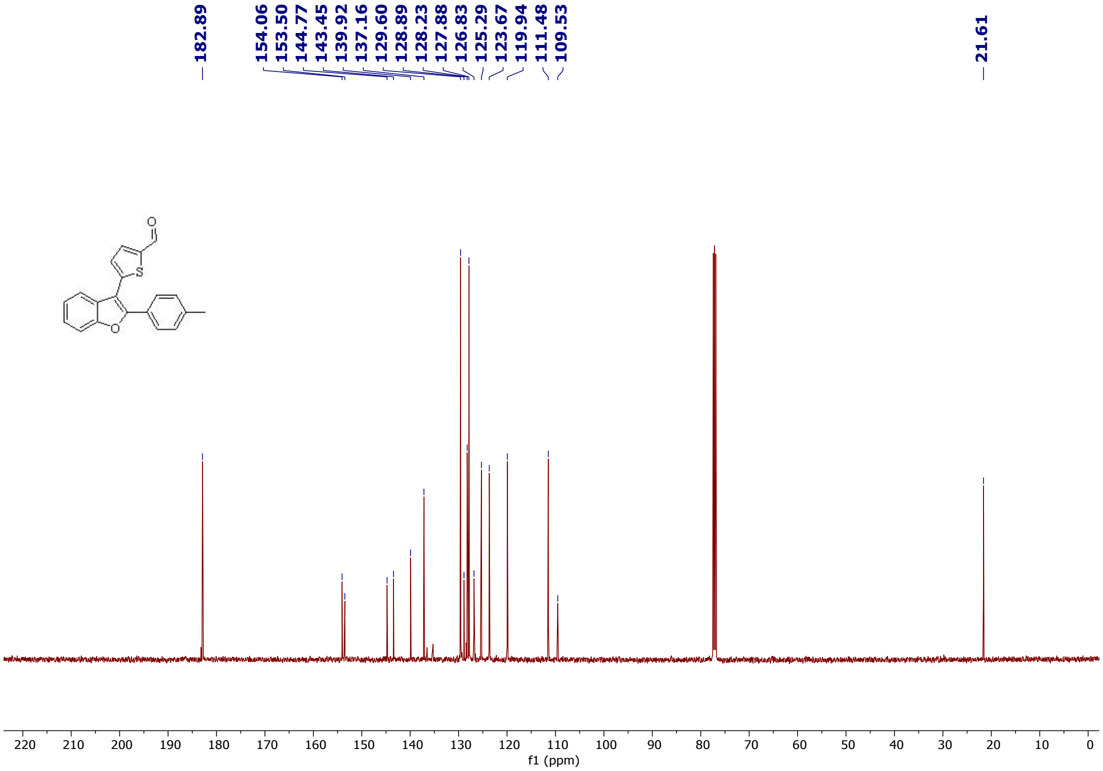

Figure S30: ^{13}C NMR spectrum of 2e

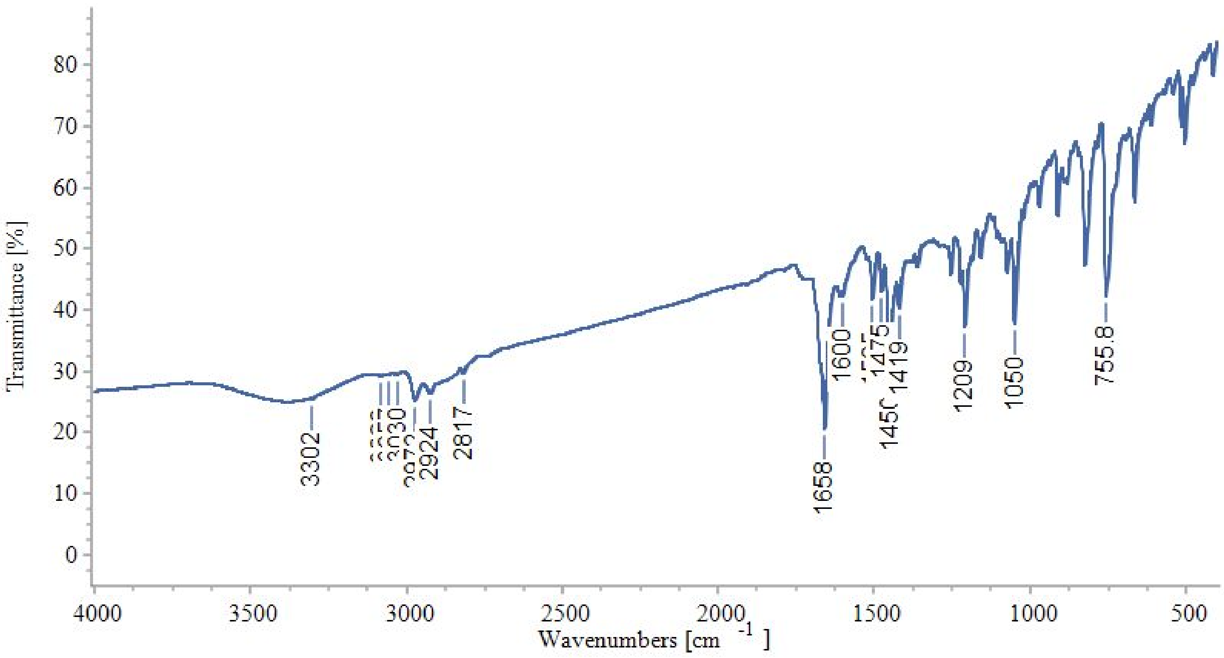

Figure S31: FT-IR spectrum of 3e

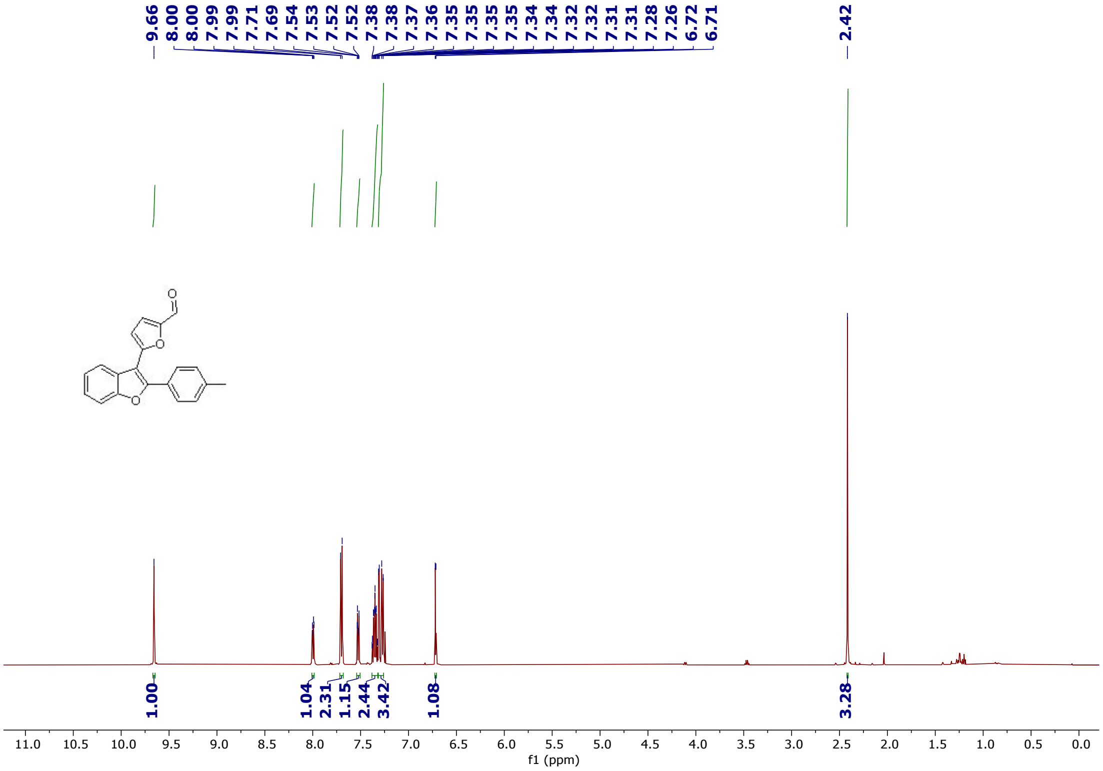

Figure S32: ^{1}H NMR spectrum of 3f

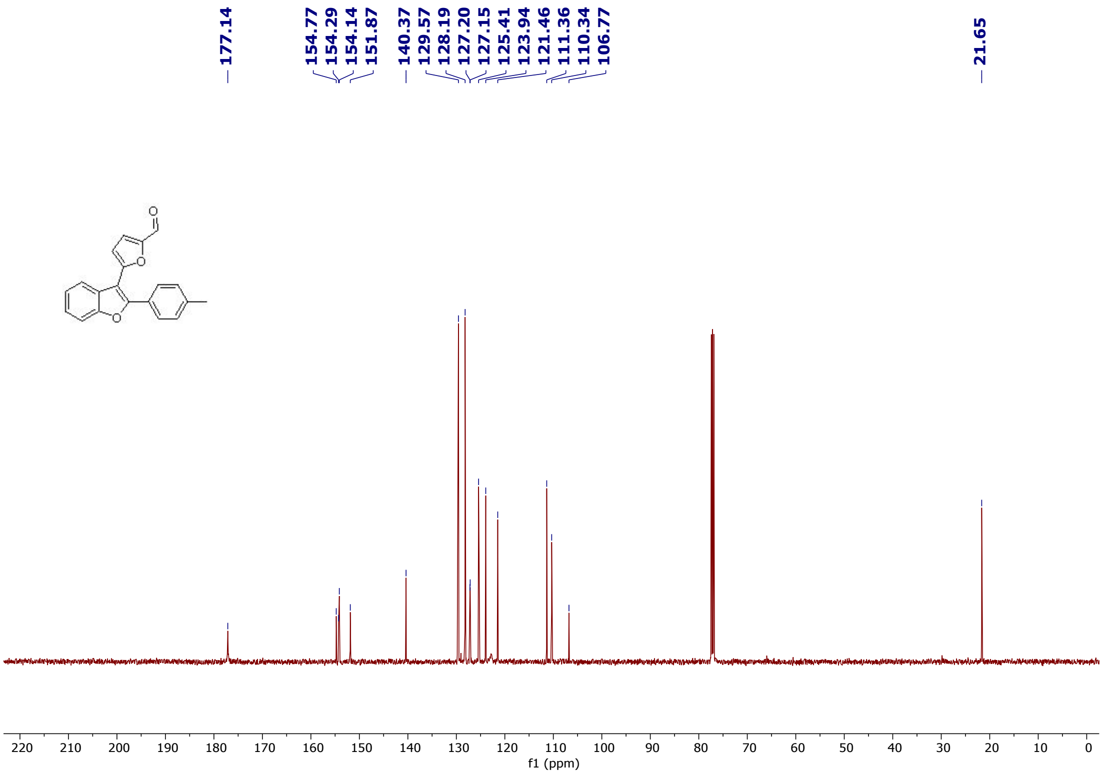

Figure S33: ^{13}C NMR spectrum of 3f

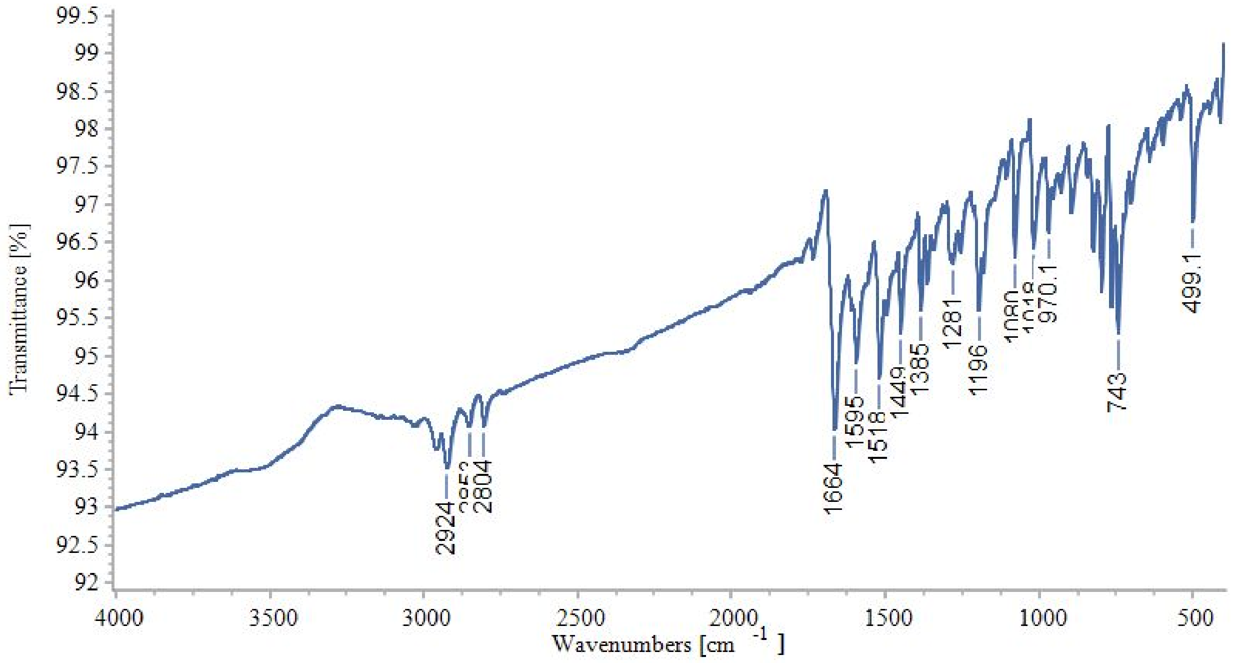

Figure S34: FT-IR spectrum of 3f

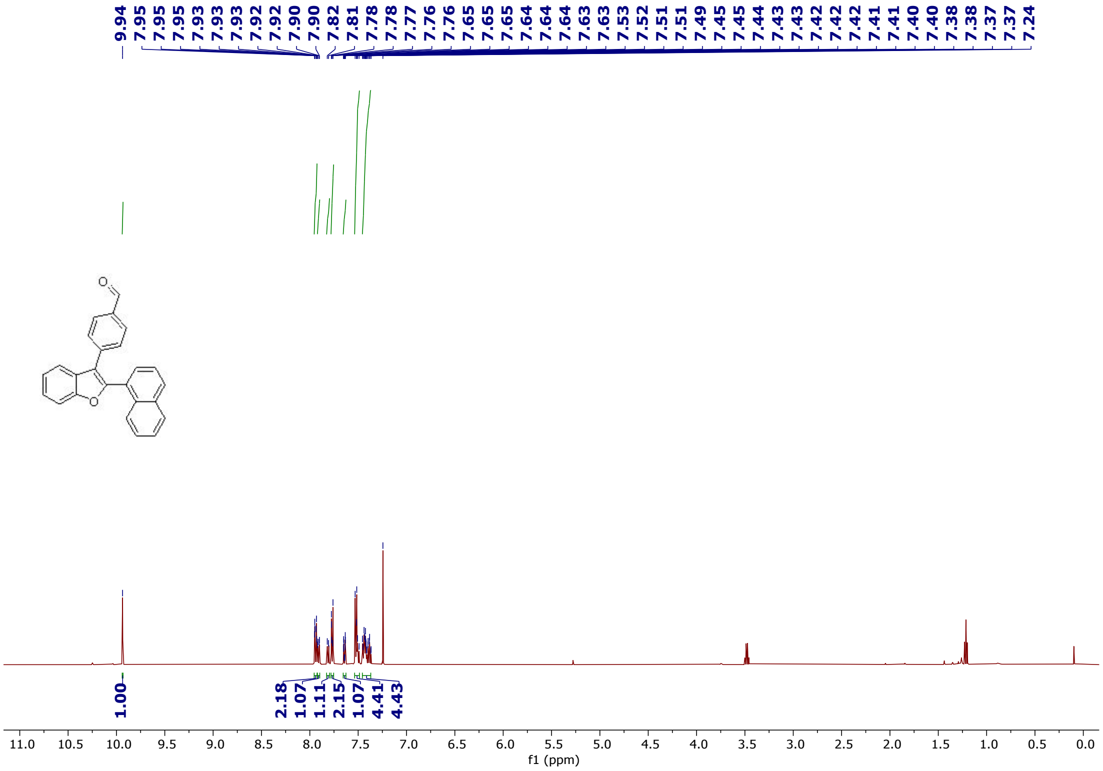

Figure S35: ^{1}H NMR spectrum of 3g

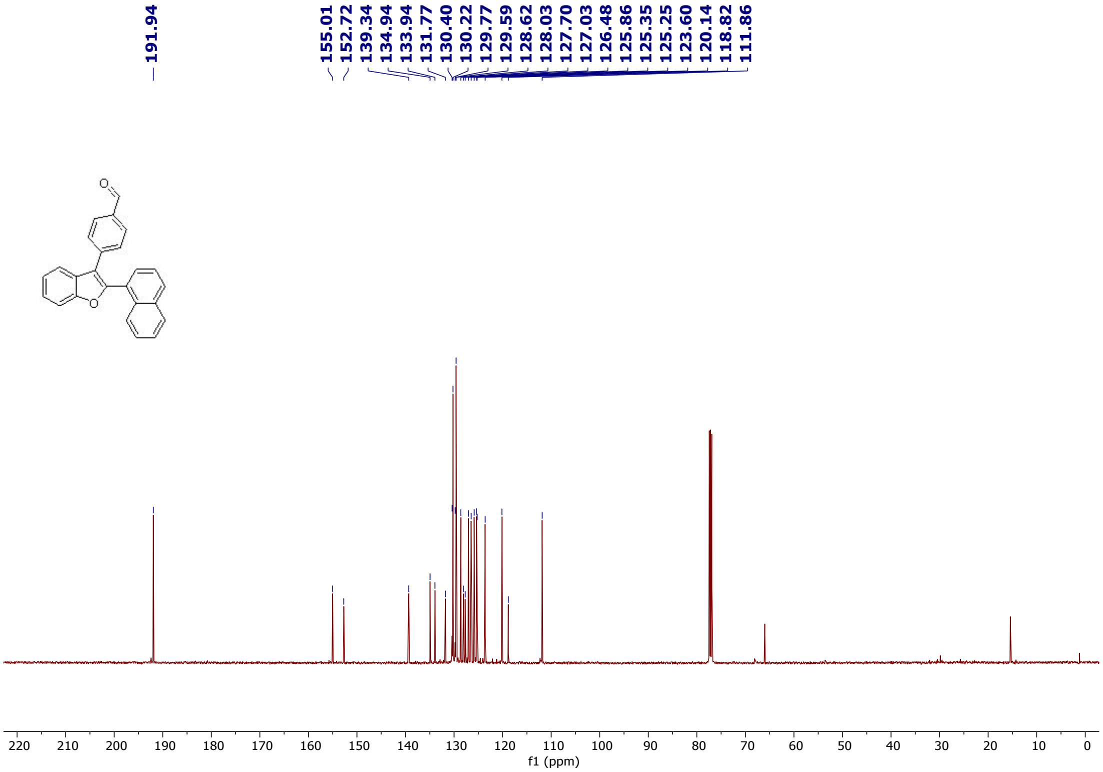

Figure S36: ^{13}C NMR spectrum of 3g

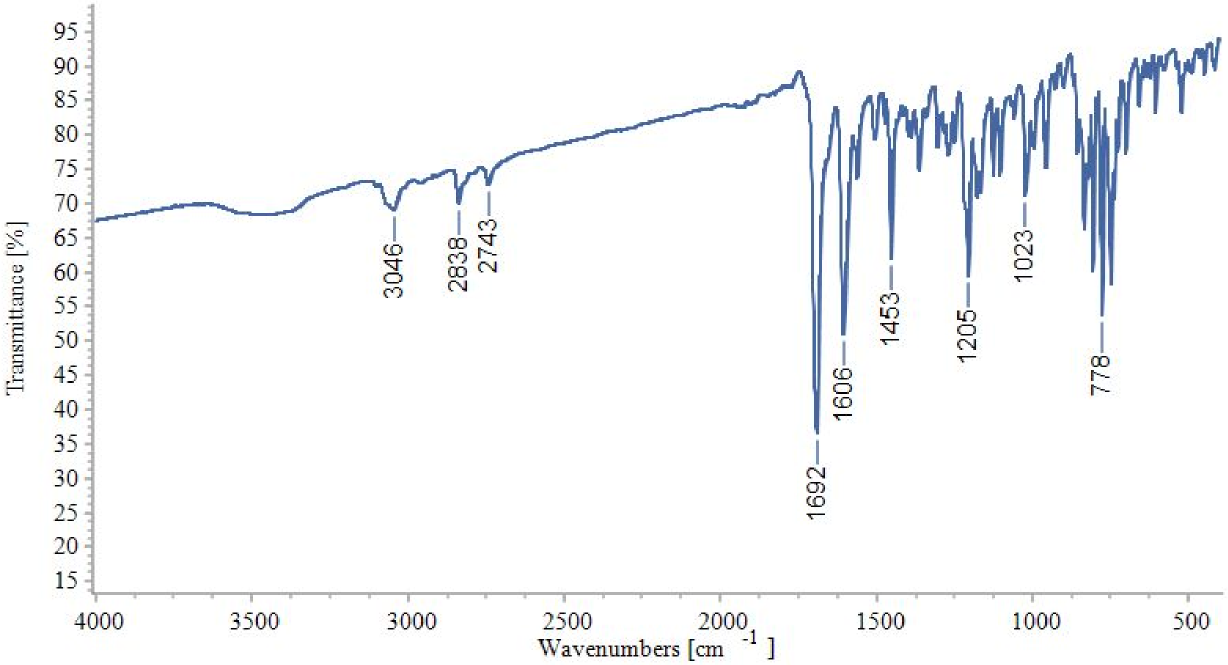

Figure S37: FT-IR spectrum of 3g

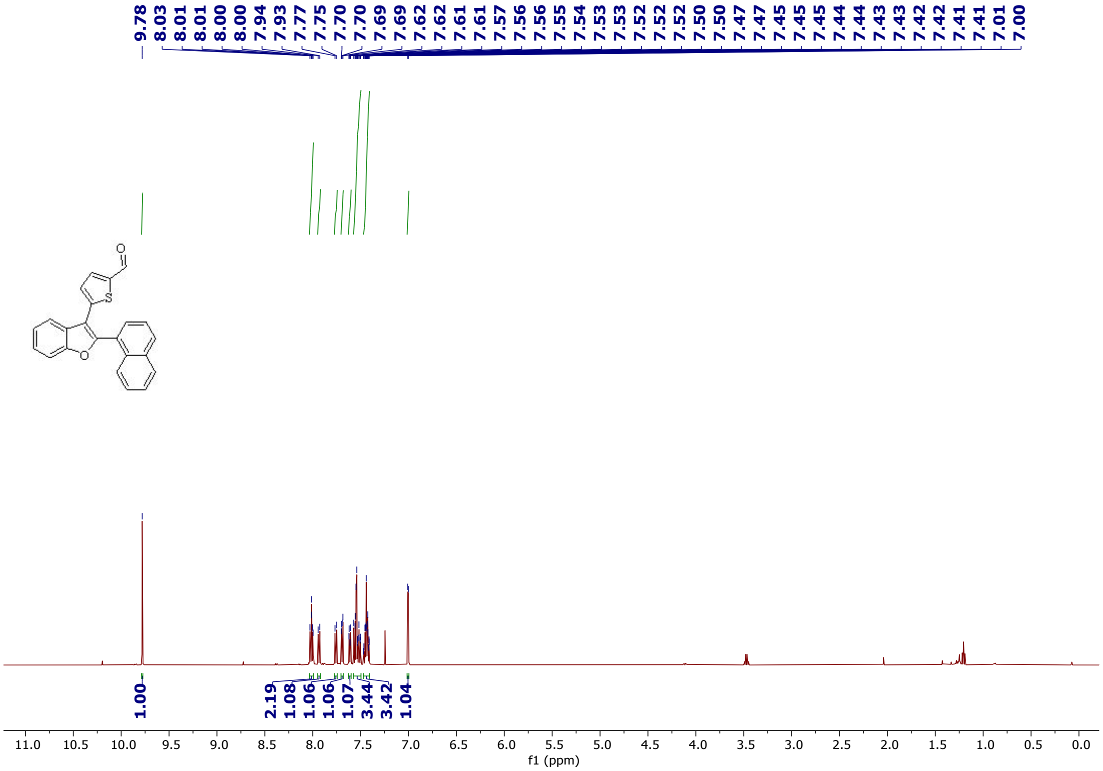

Figure S38: ^{1}H NMR spectrum of 3h

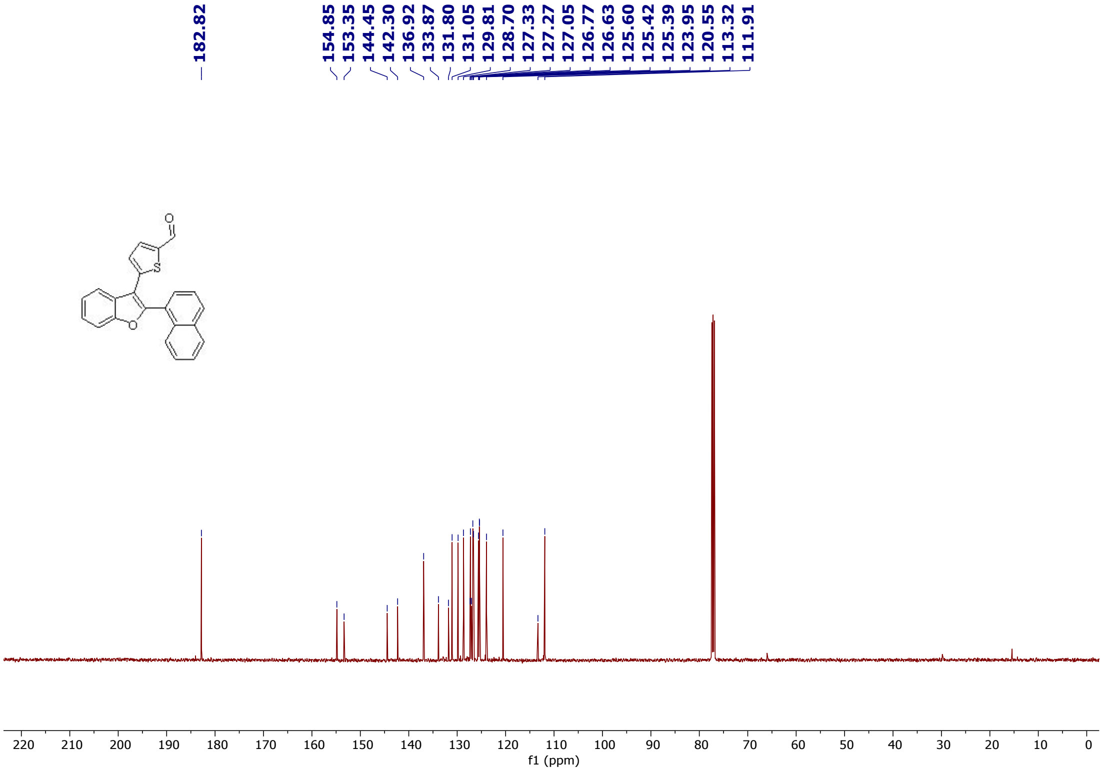

Figure S39: ^{13}C NMR spectrum of 3h

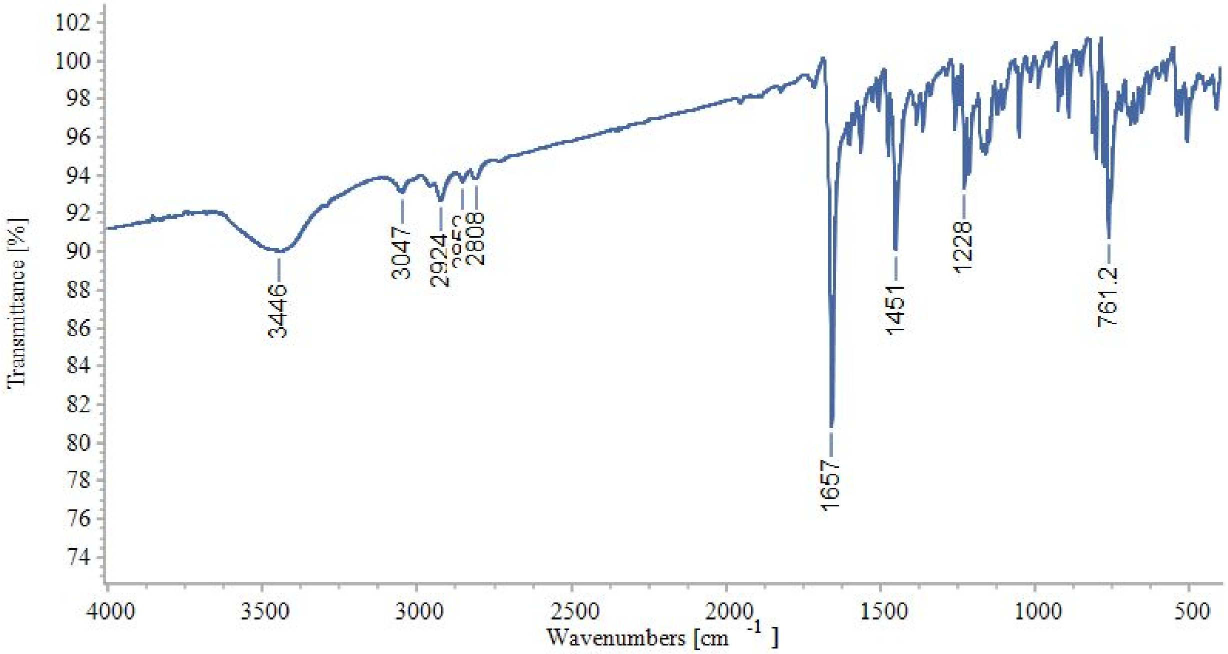

Figure S40: FT-IR spectrum of 3h

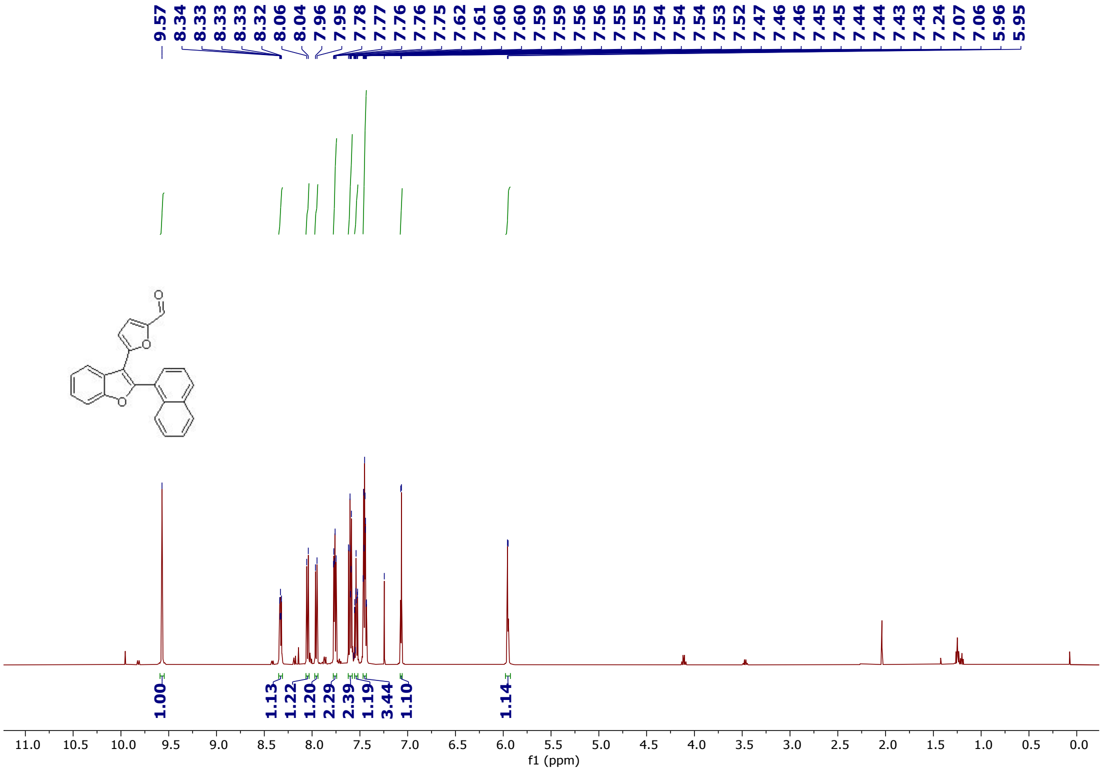

Figure S41: ^{1}H NMR spectrum of 3i

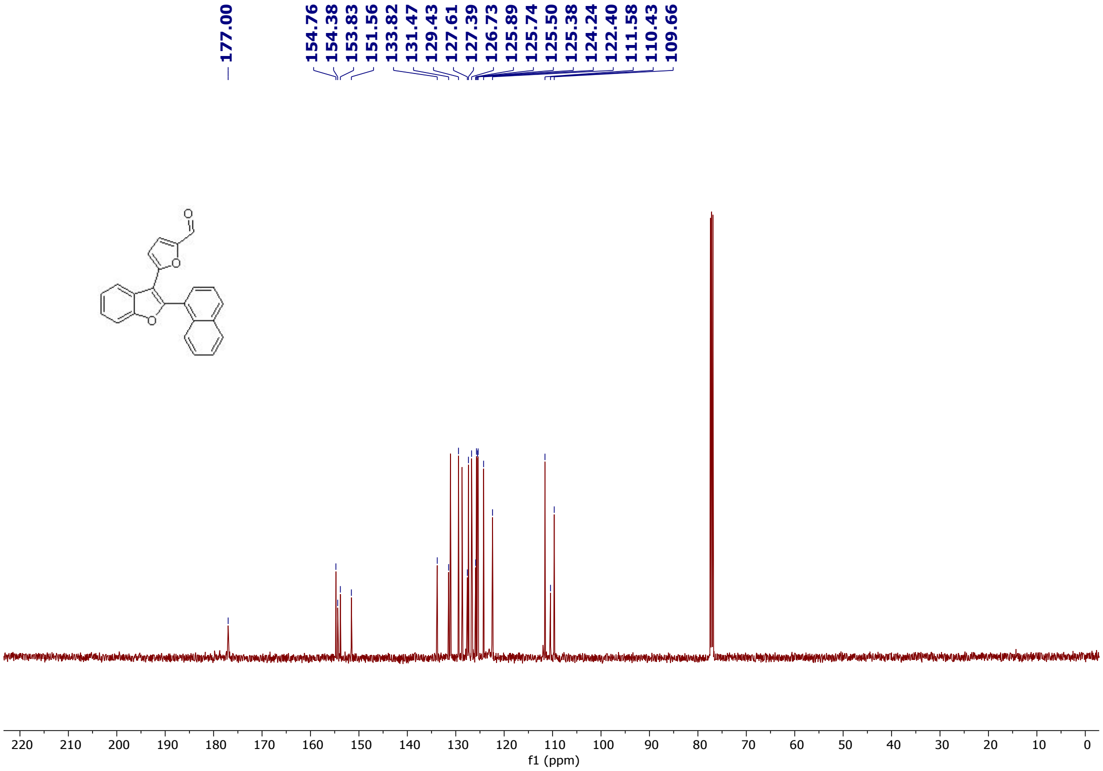

Figure S42: ^{13}C NMR spectrum of 3i

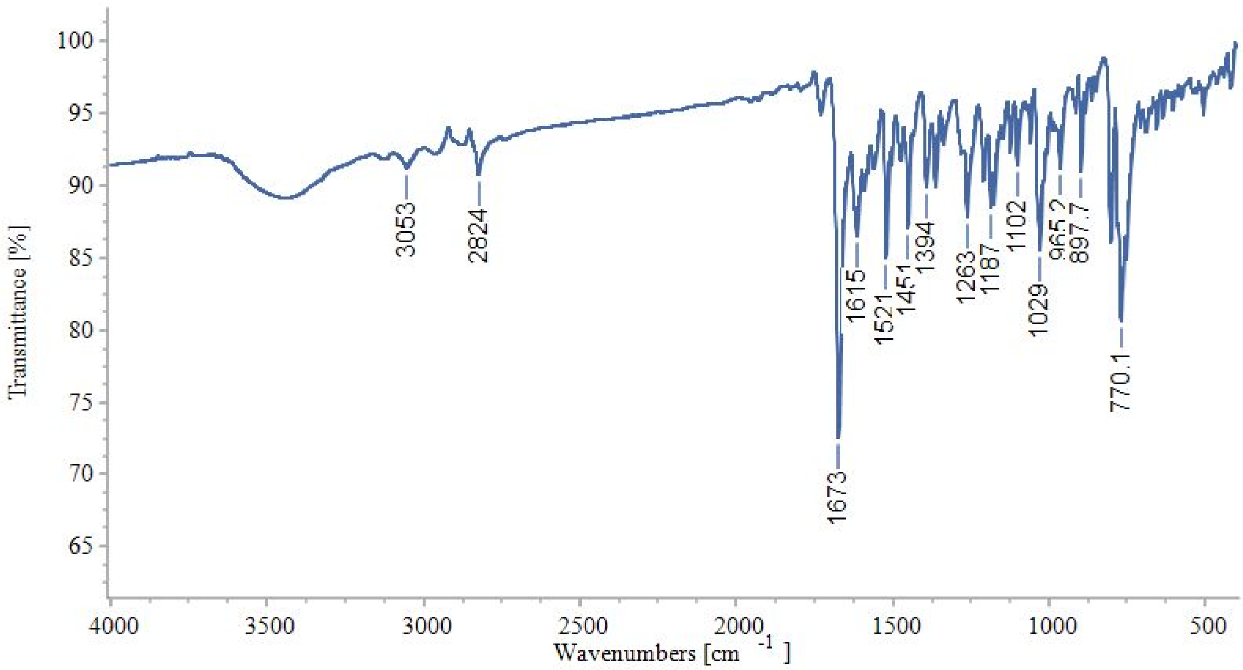

Figure S43: FT-IR spectrum of 3i

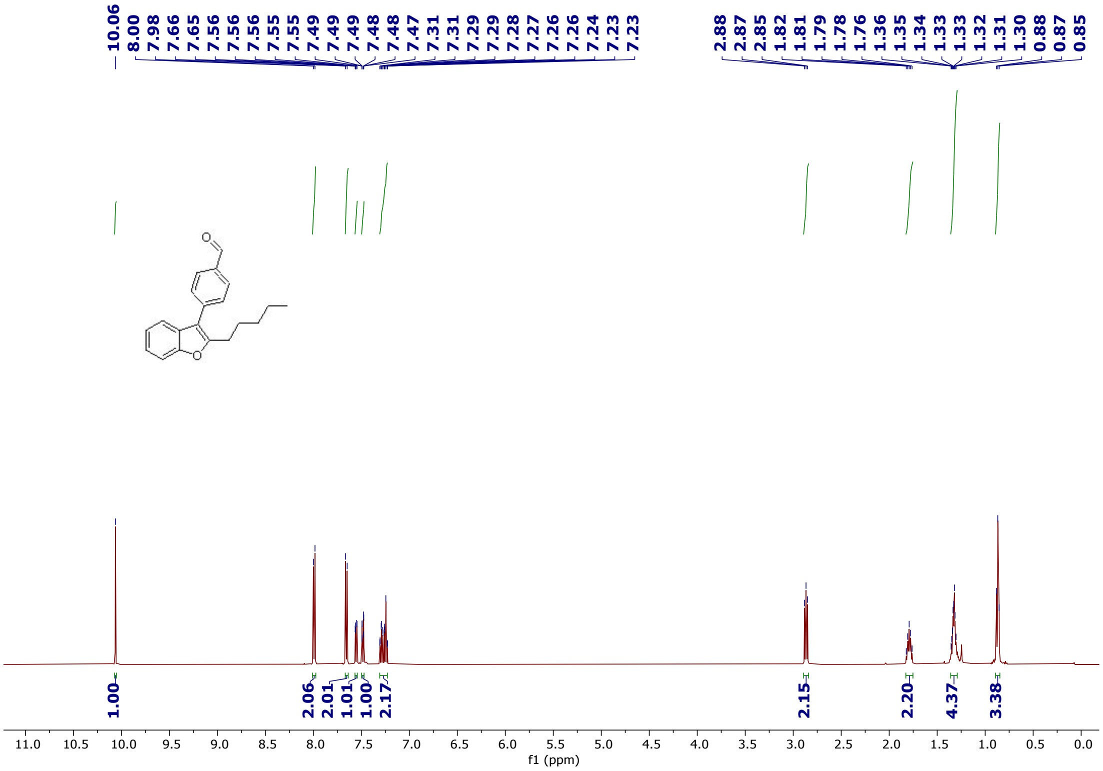

Figure S44: ^{1}H NMR spectrum of 3j

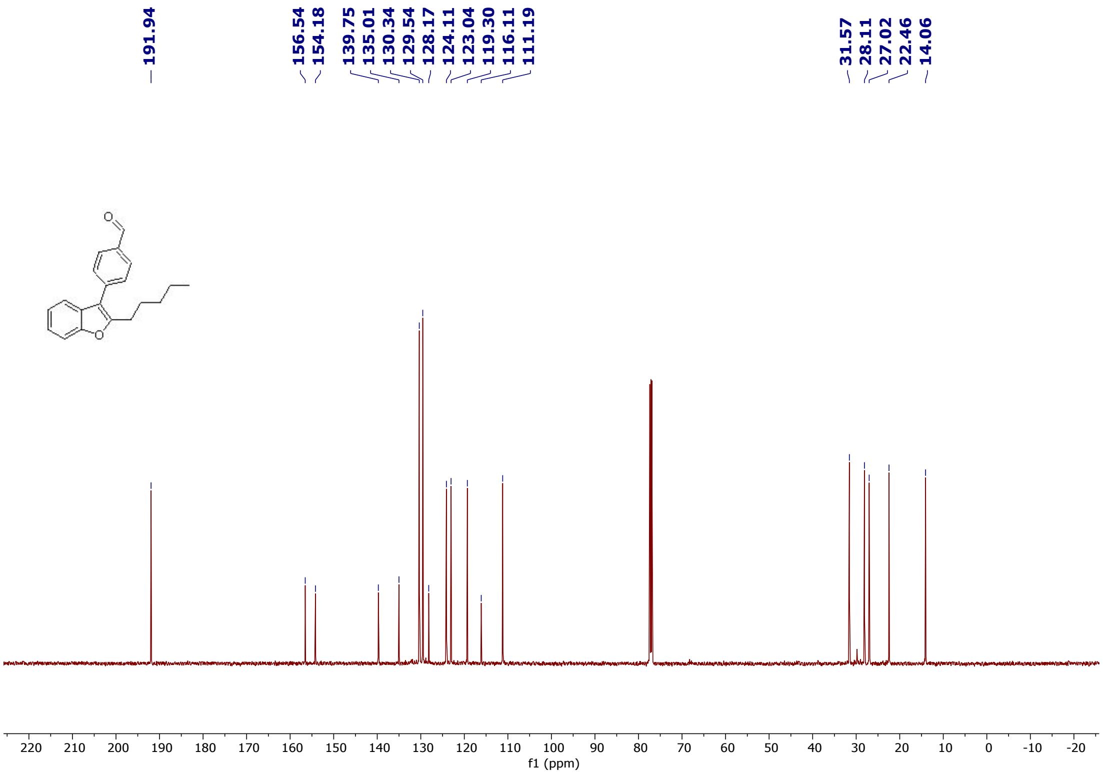

Figure S45: ^{13}C NMR spectrum of 3j

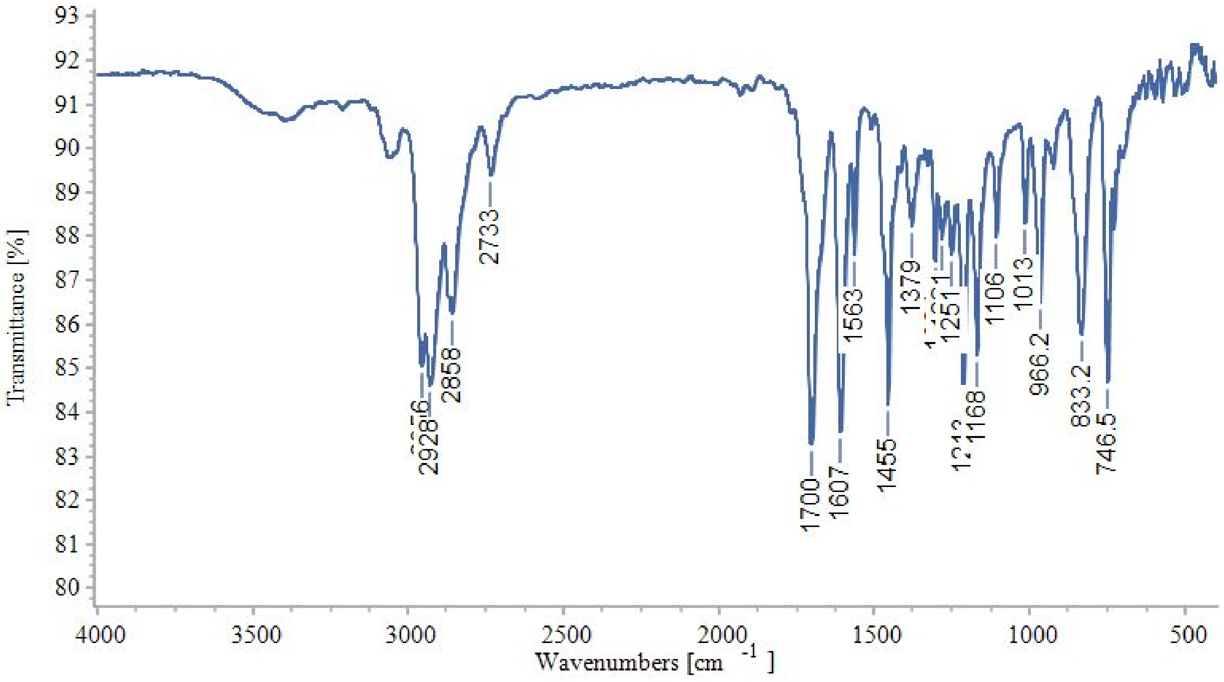

Figure S46: FT-IR spectrum of 3j

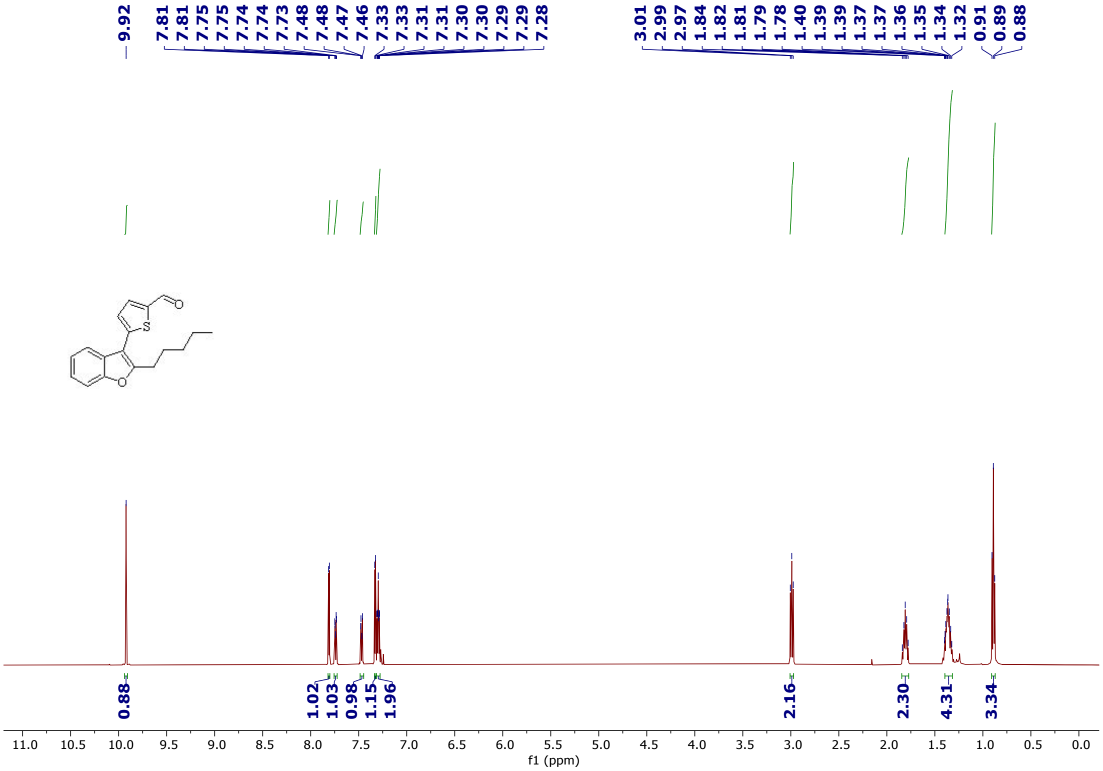

Figure S47: ^{1}H NMR spectrum of 3k

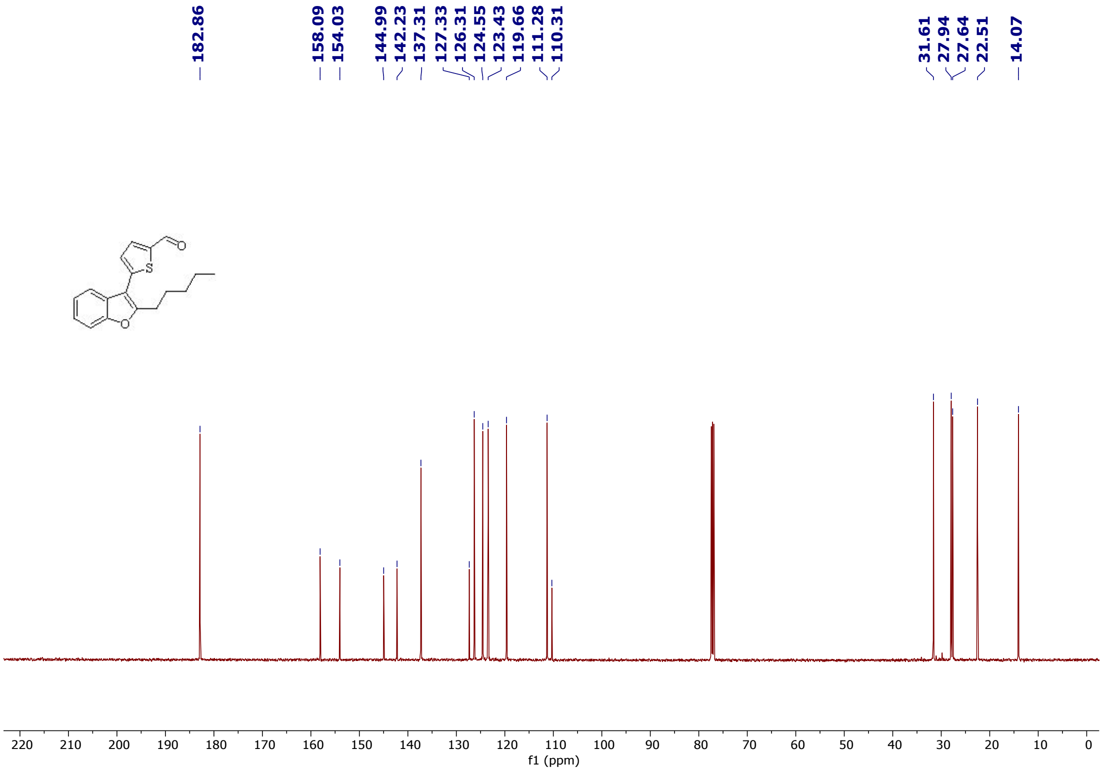

Figure S48: ^{13}C NMR spectrum of 3k

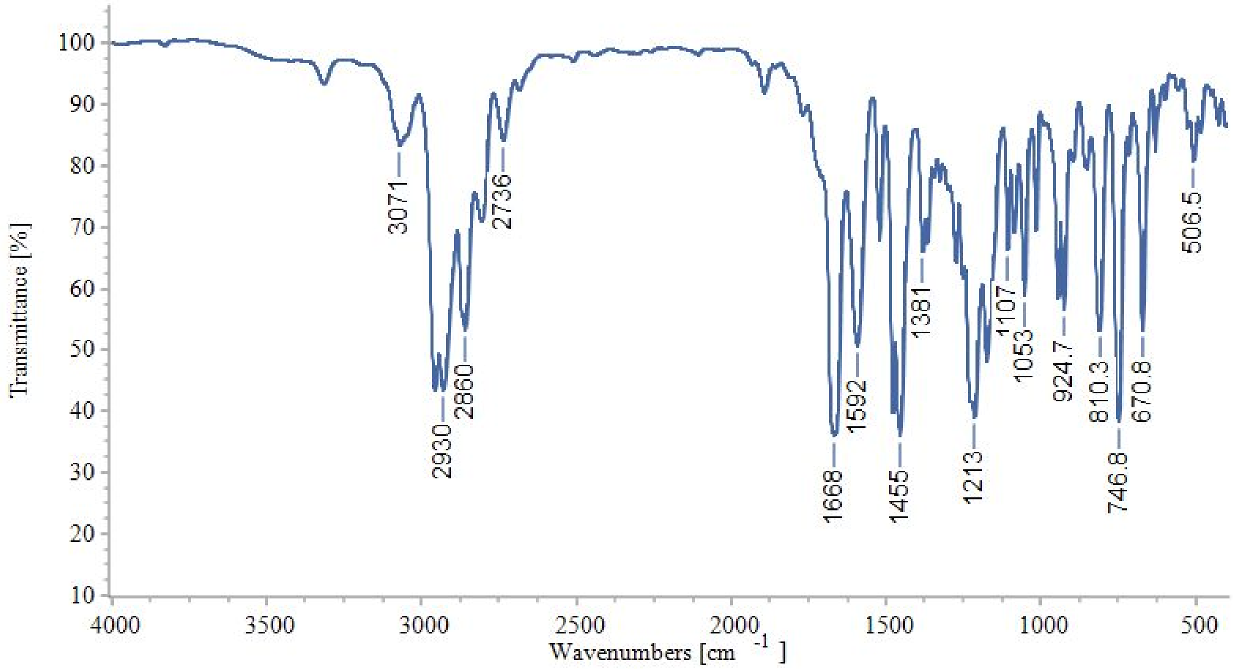

Figure S49: FT-IR spectrum of 3k

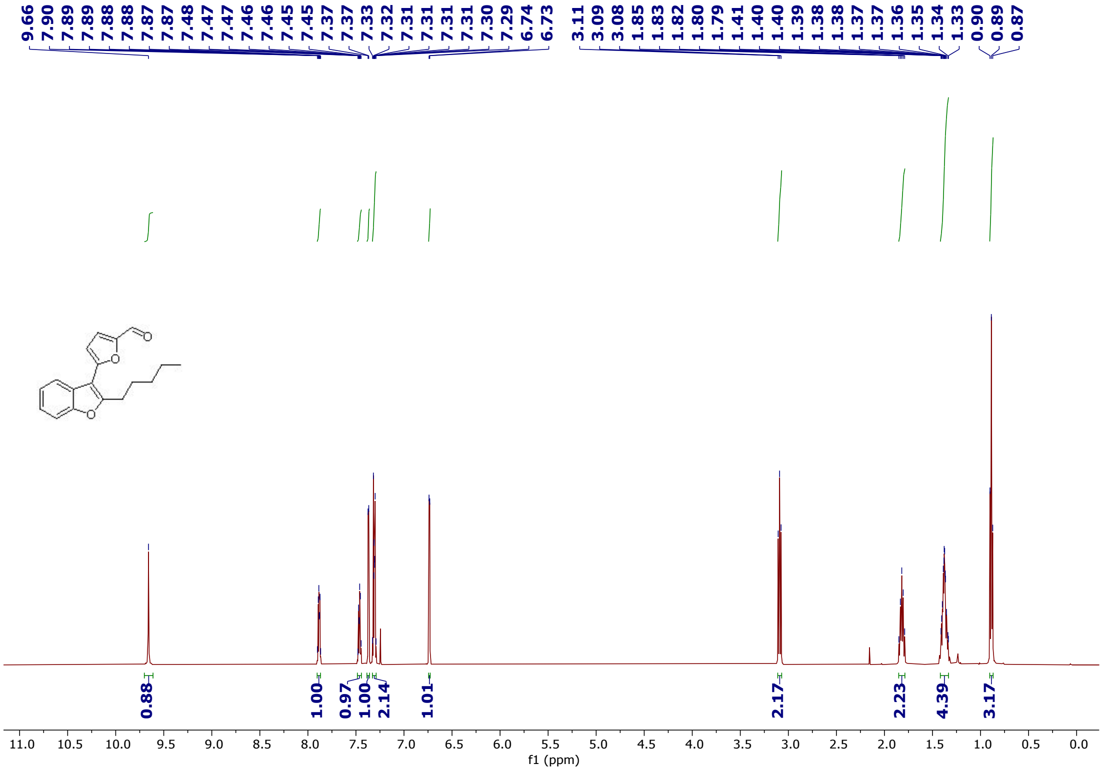

Figure S50: ^{1}H NMR spectrum of 3l

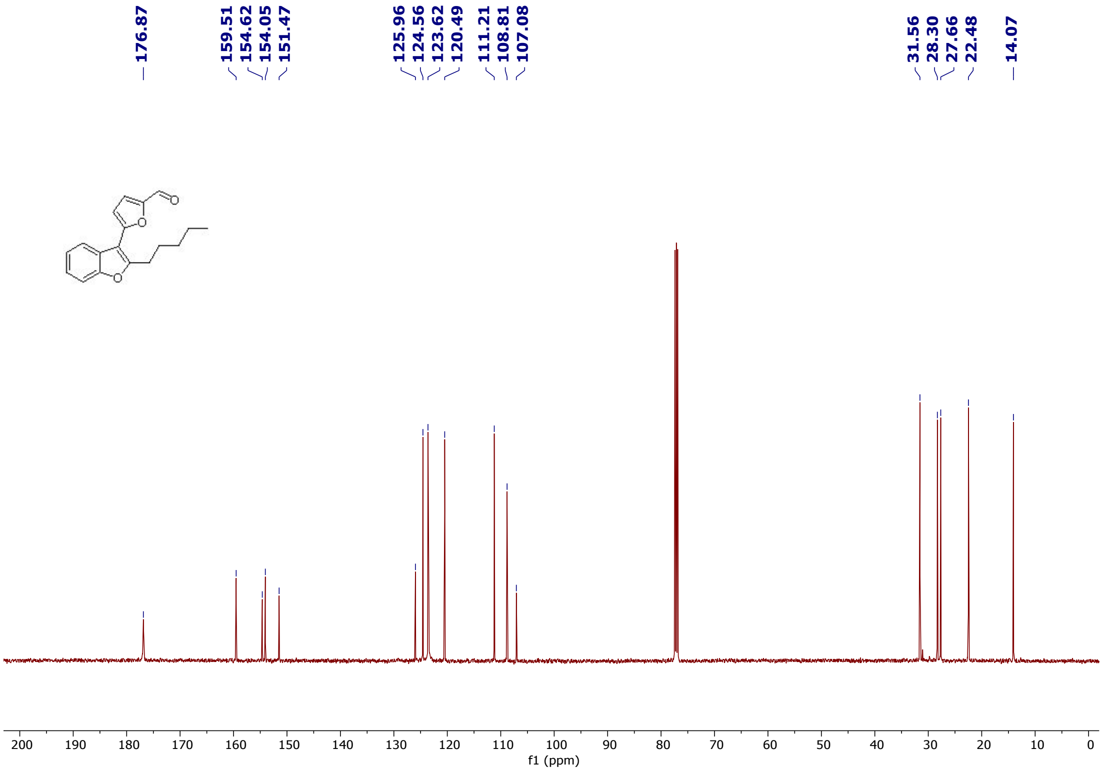

Figure S51: ^{13}C NMR spectrum of 3l

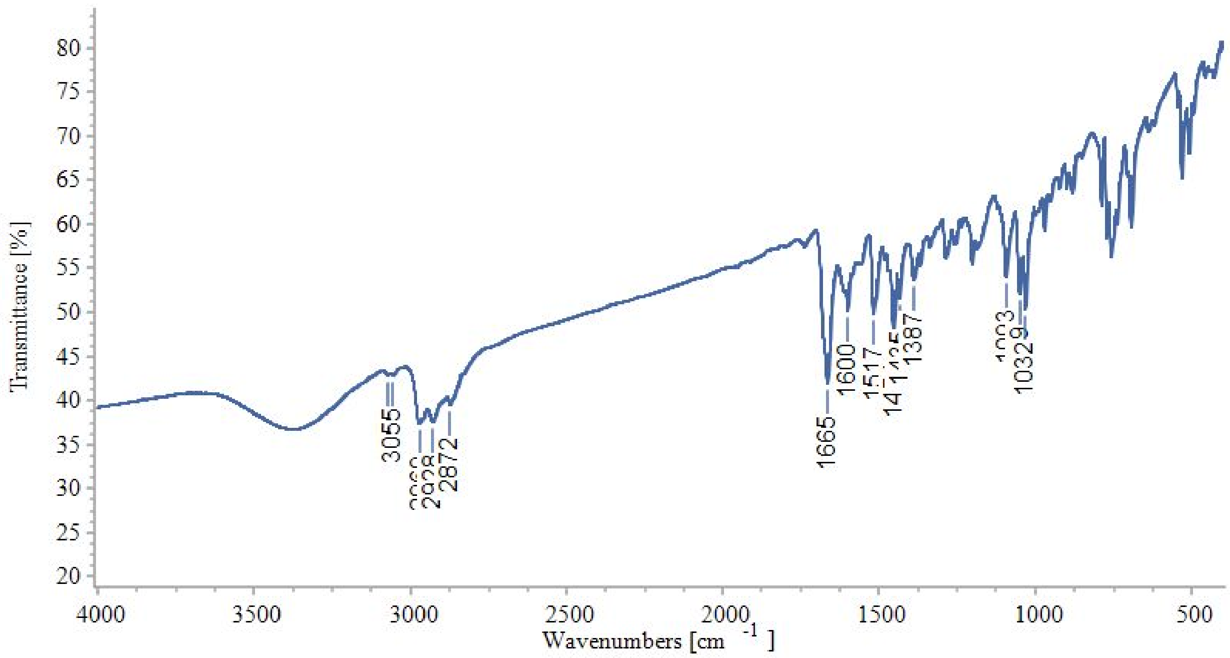

Figure S52: FT-IR spectrum of 3l

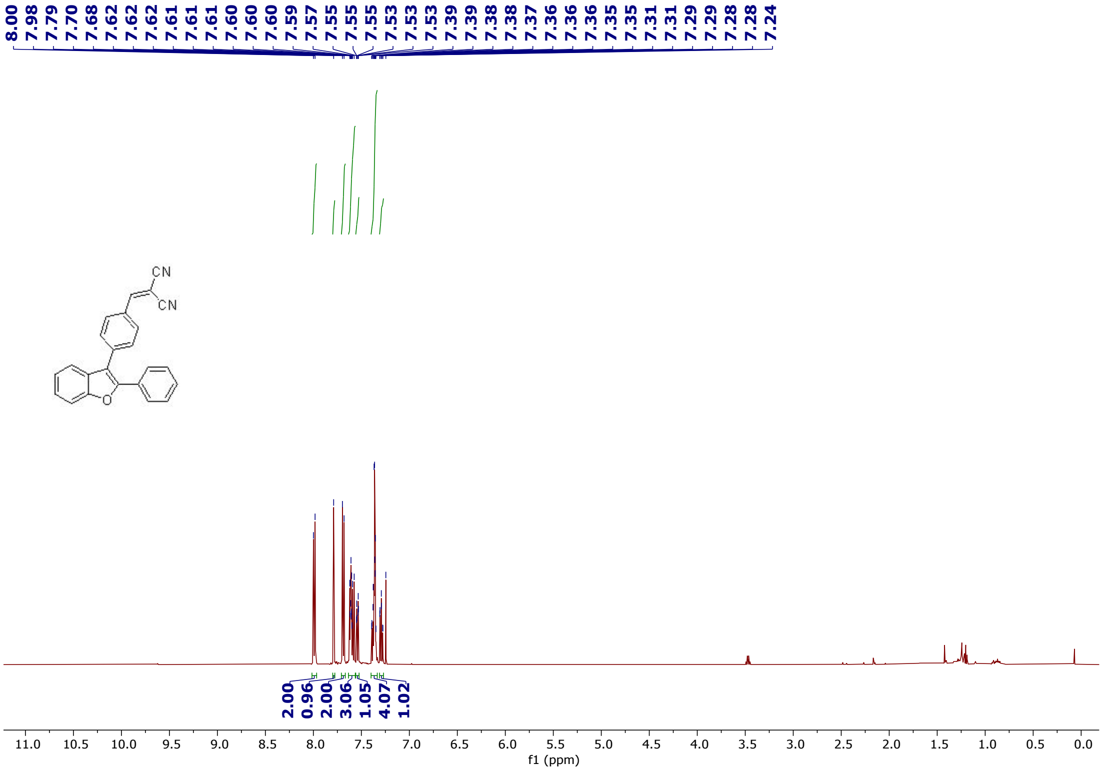

Figure S53: ^{1}H NMR spectrum of 4a

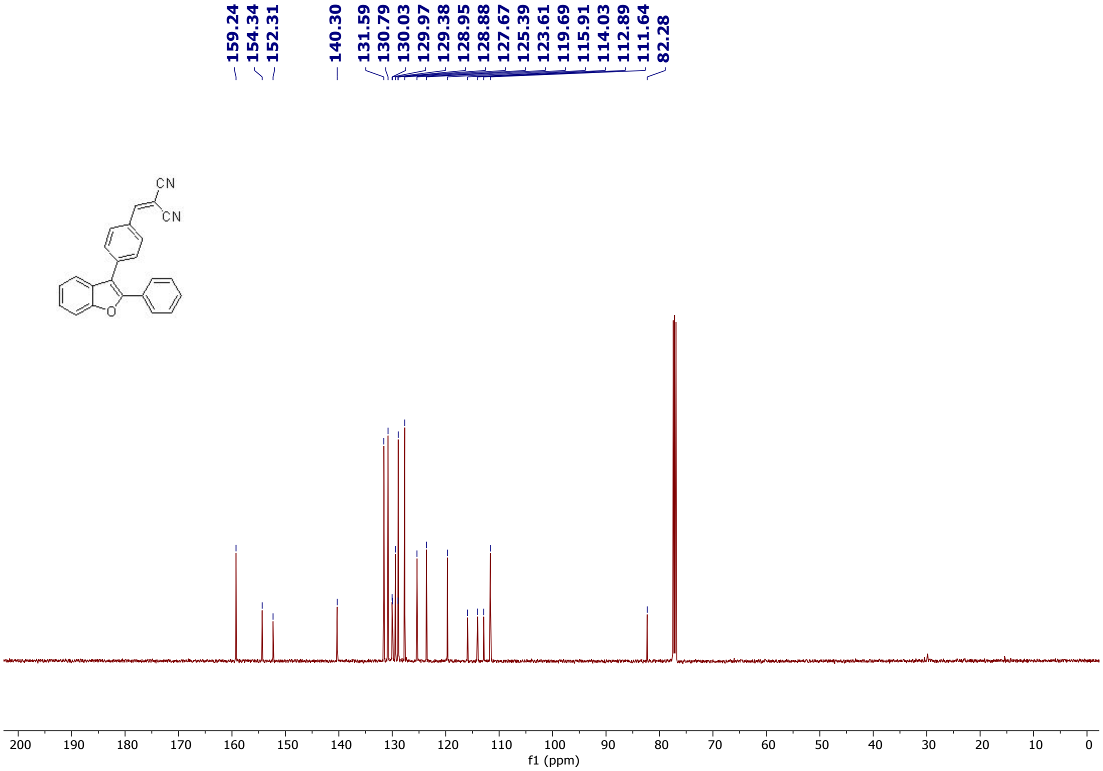

Figure S54: ^{13}C NMR spectrum of 4a

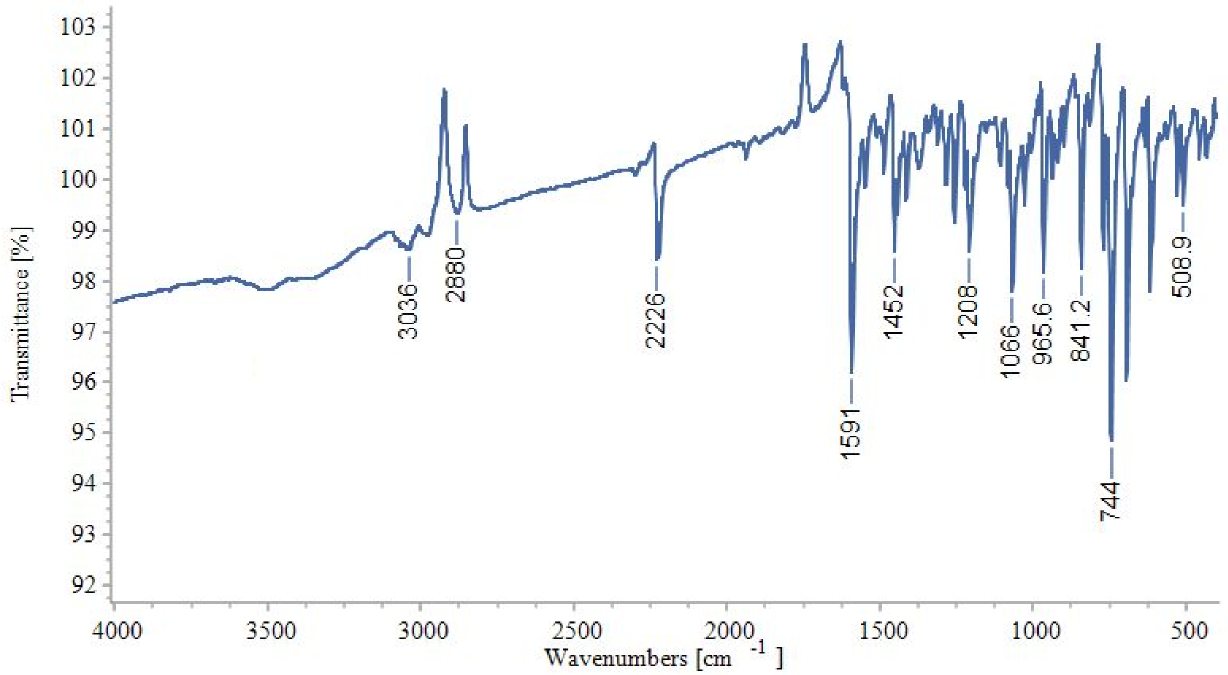

Figure S55: FT-IR spectrum of 4a

H$^+$

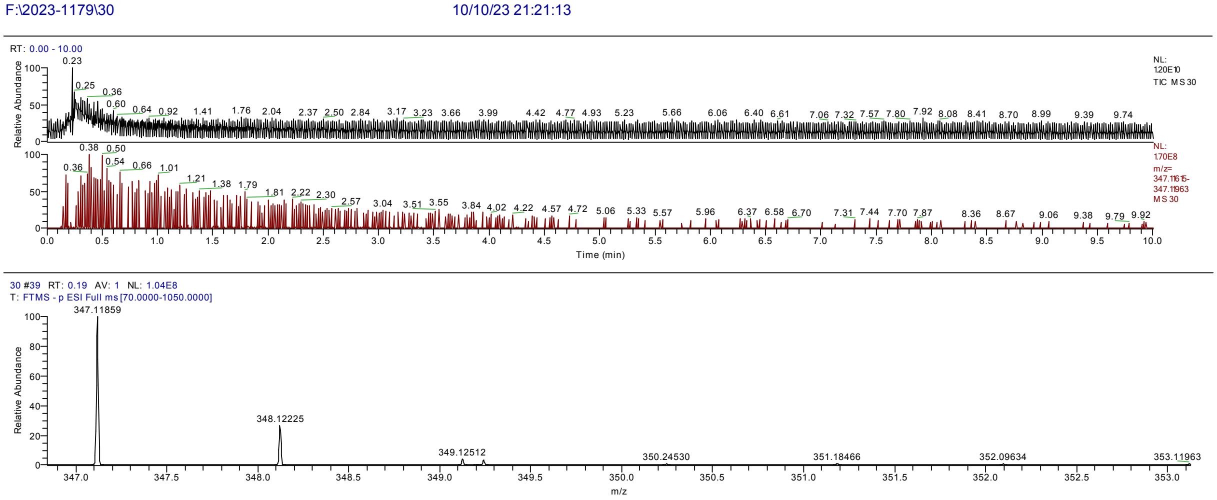

Theoretical

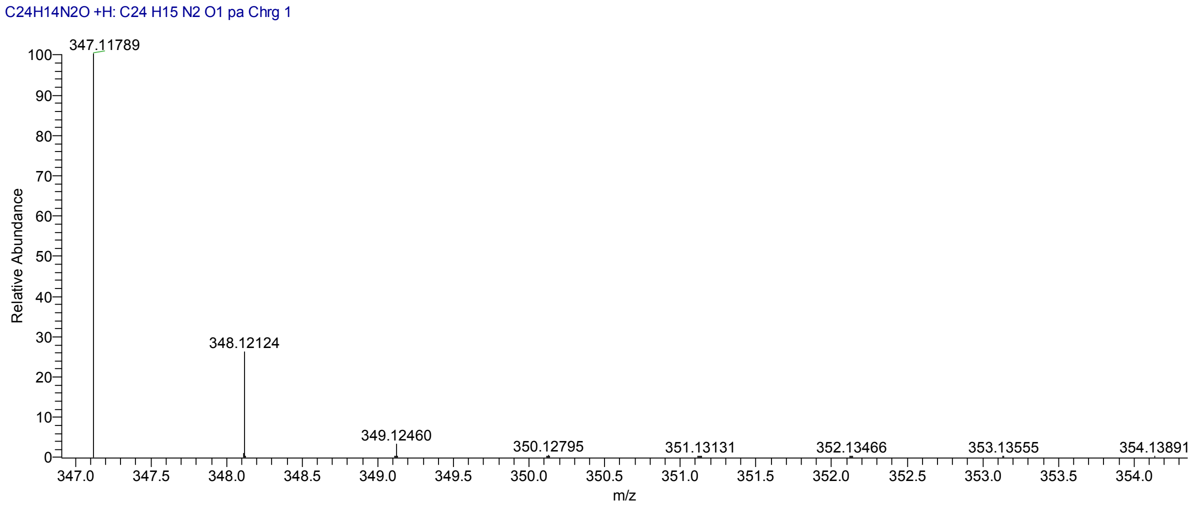

Figure S56: mass spectra of 4a

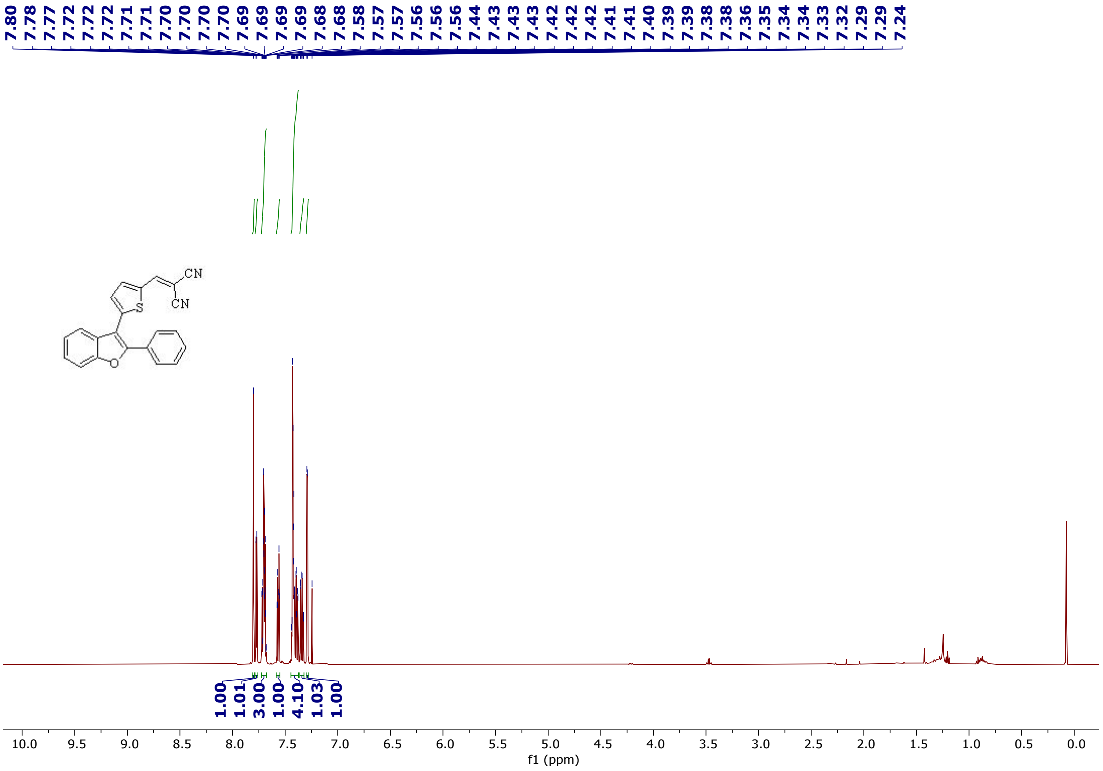

Figure S57: ^{1}H NMR spectrum of 4b

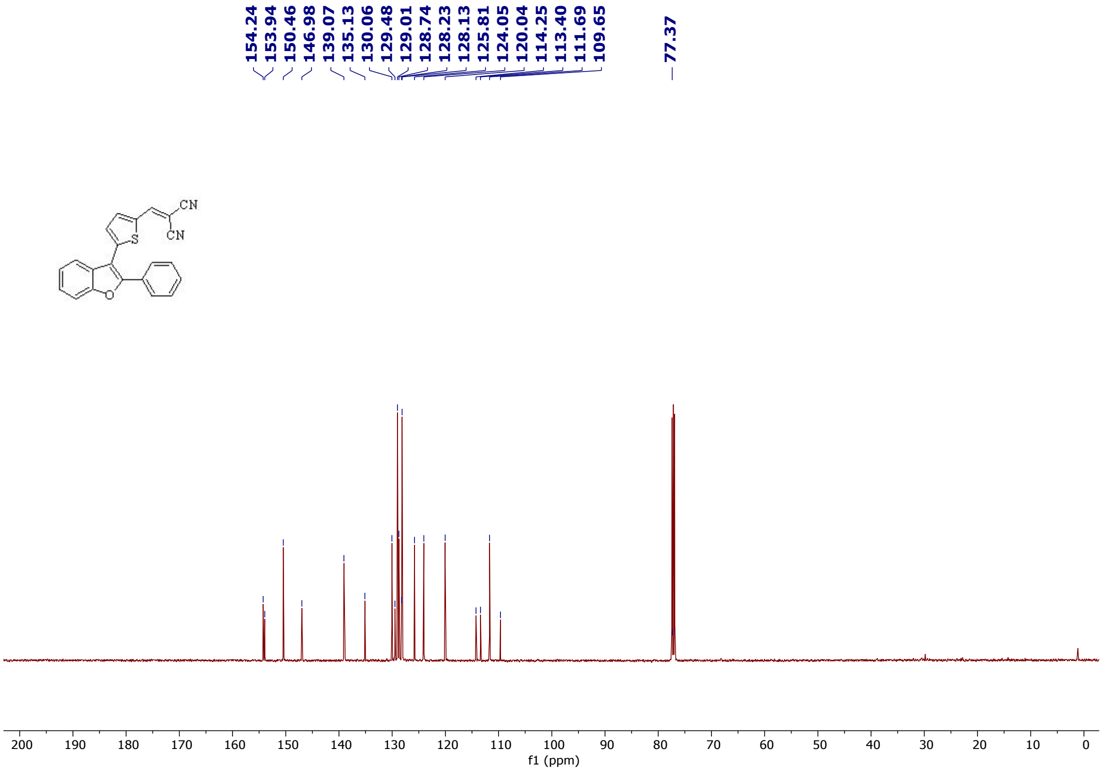

Figure S58: ^{13}C NMR spectrum of 4b

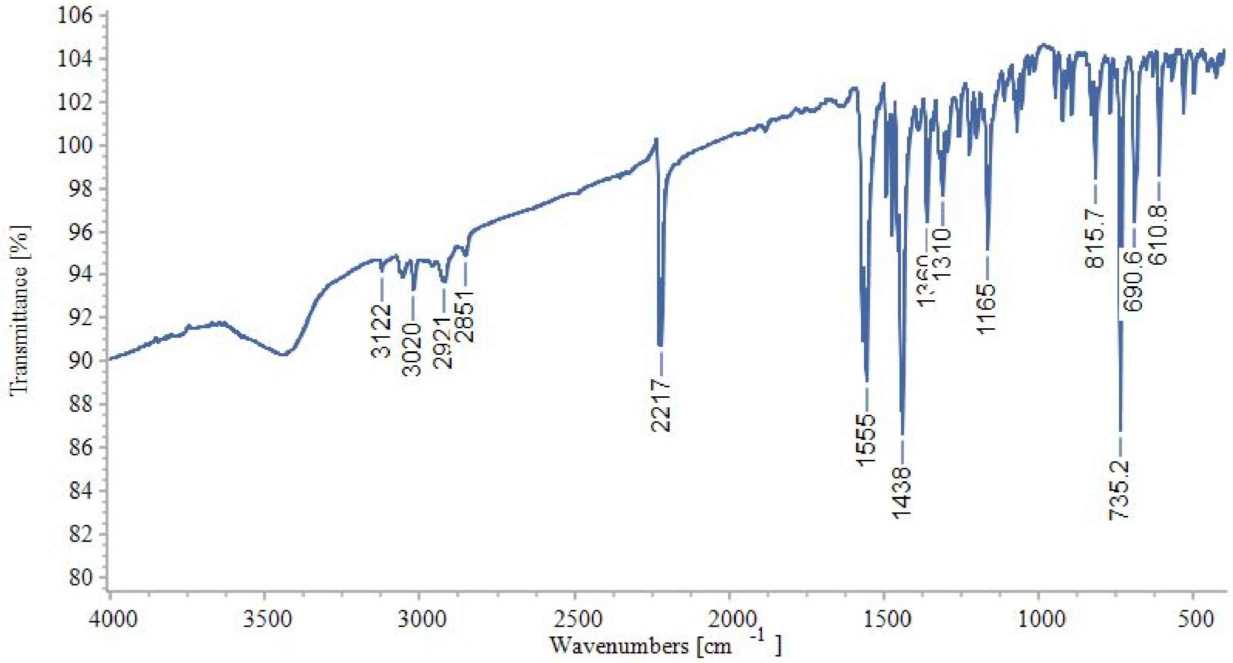

Figure S59: FT-IR spectrum of 4b

H^+

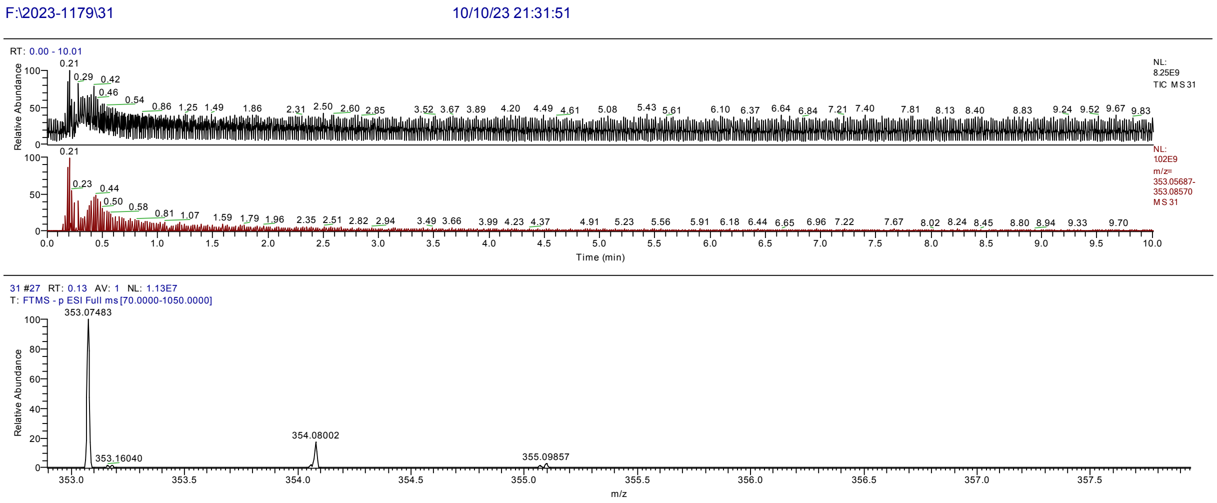

Theoretical

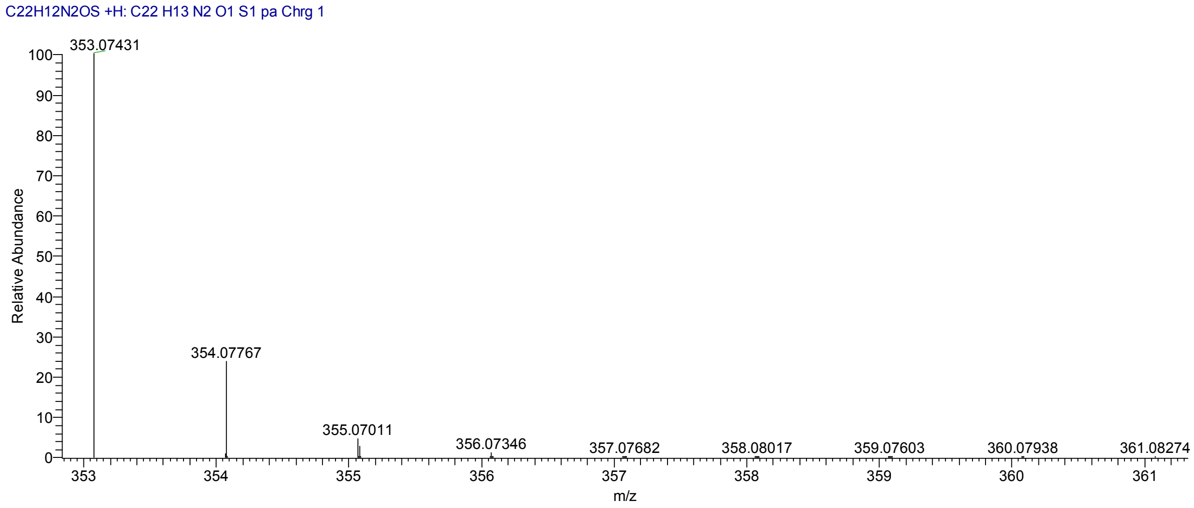

Figure S60: mas spectra of 4b

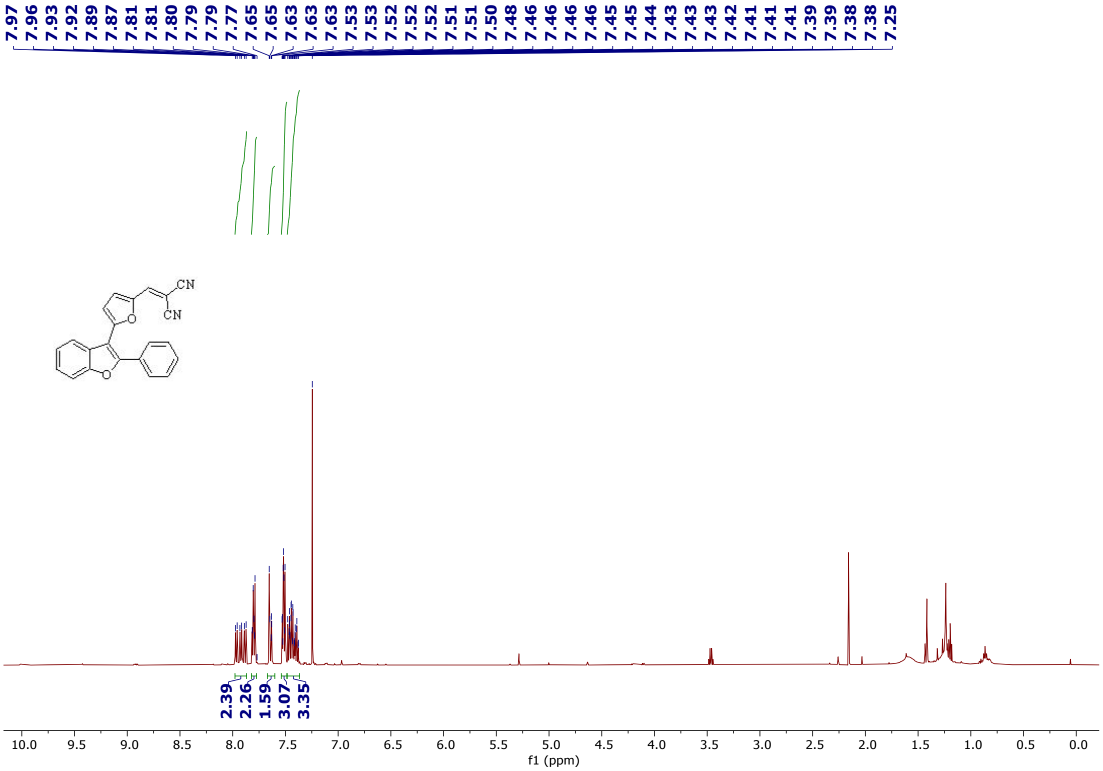

Figure S61: ^{1}H NMR spectrum of 4c

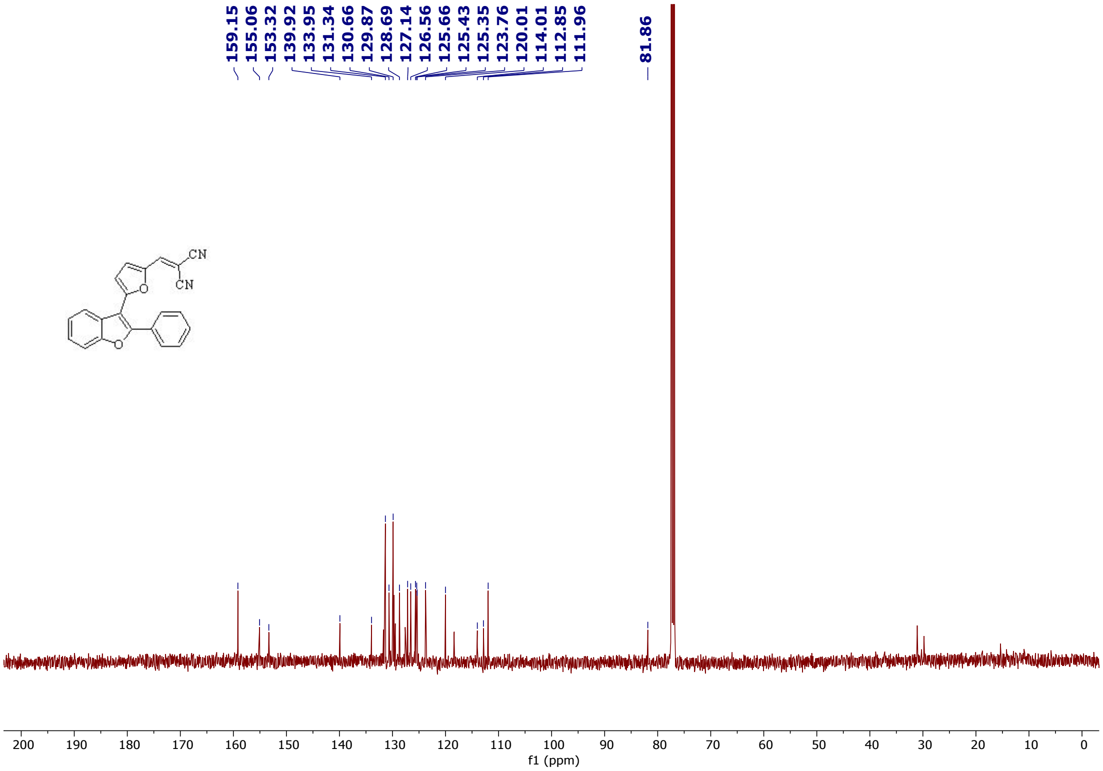

Figure S62: ^{13}C NMR spectrum of 4c

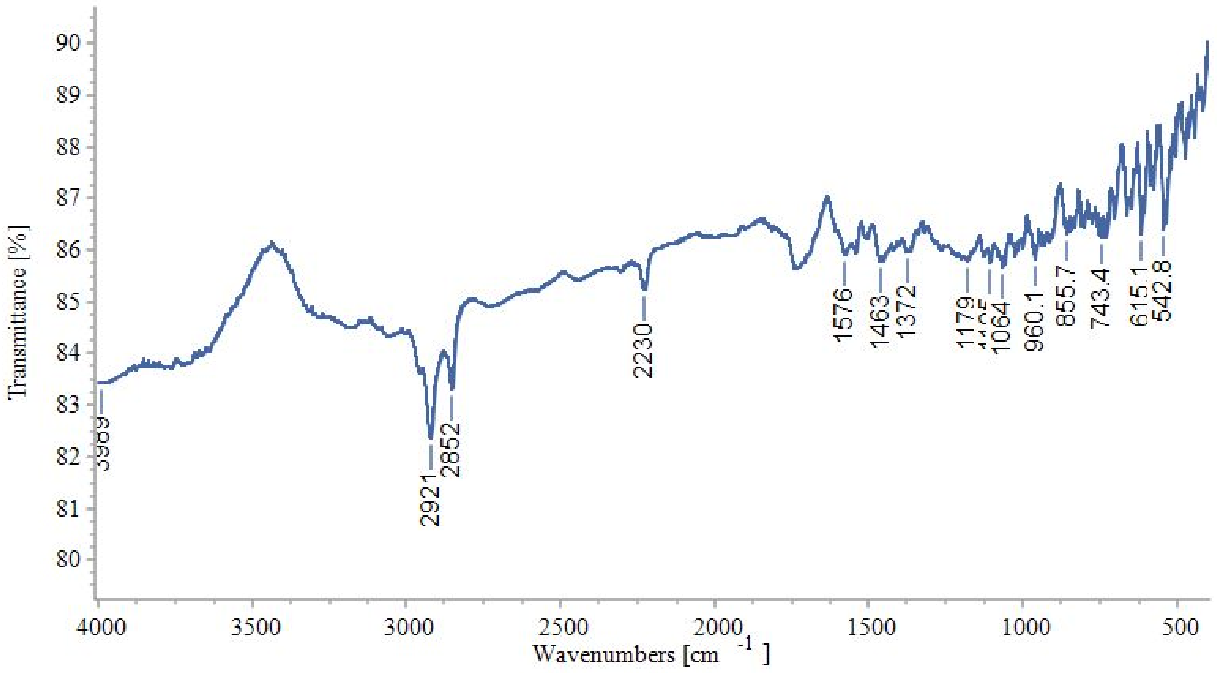

Figure S63: FT-IR spectrum of 4c

Na$^+$

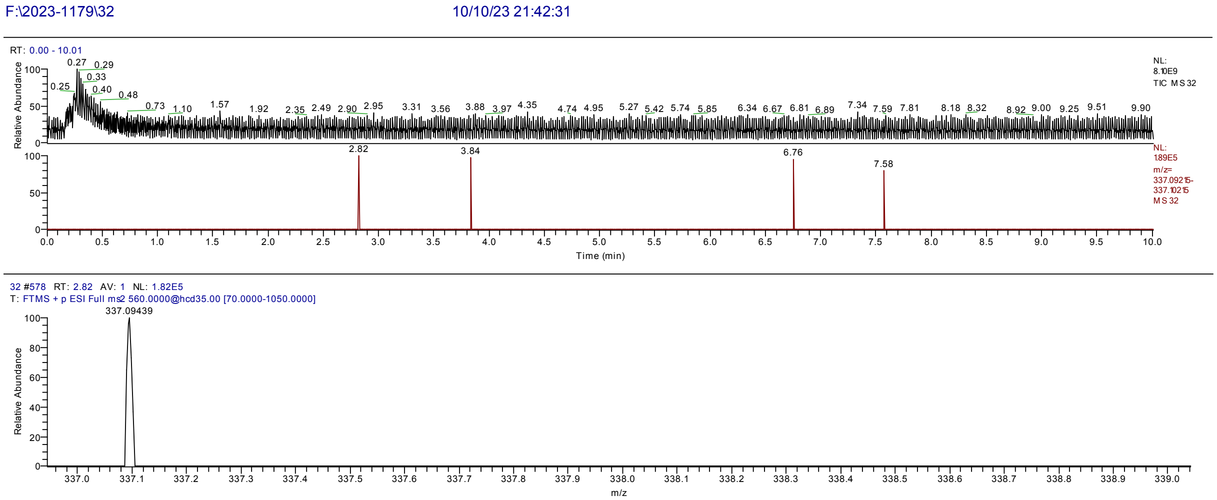

Theoretical

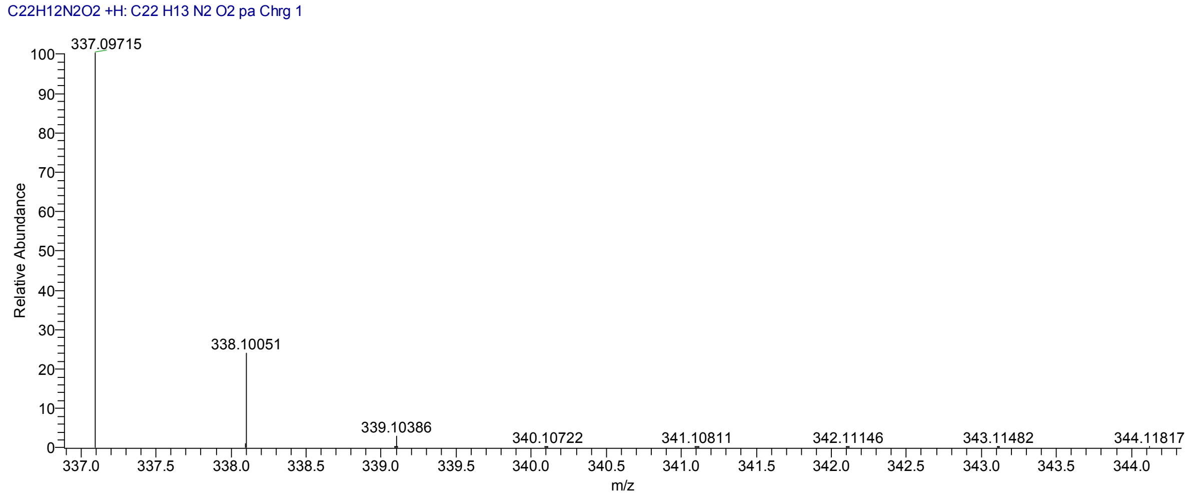

Figure S64: mass spectra of 4c

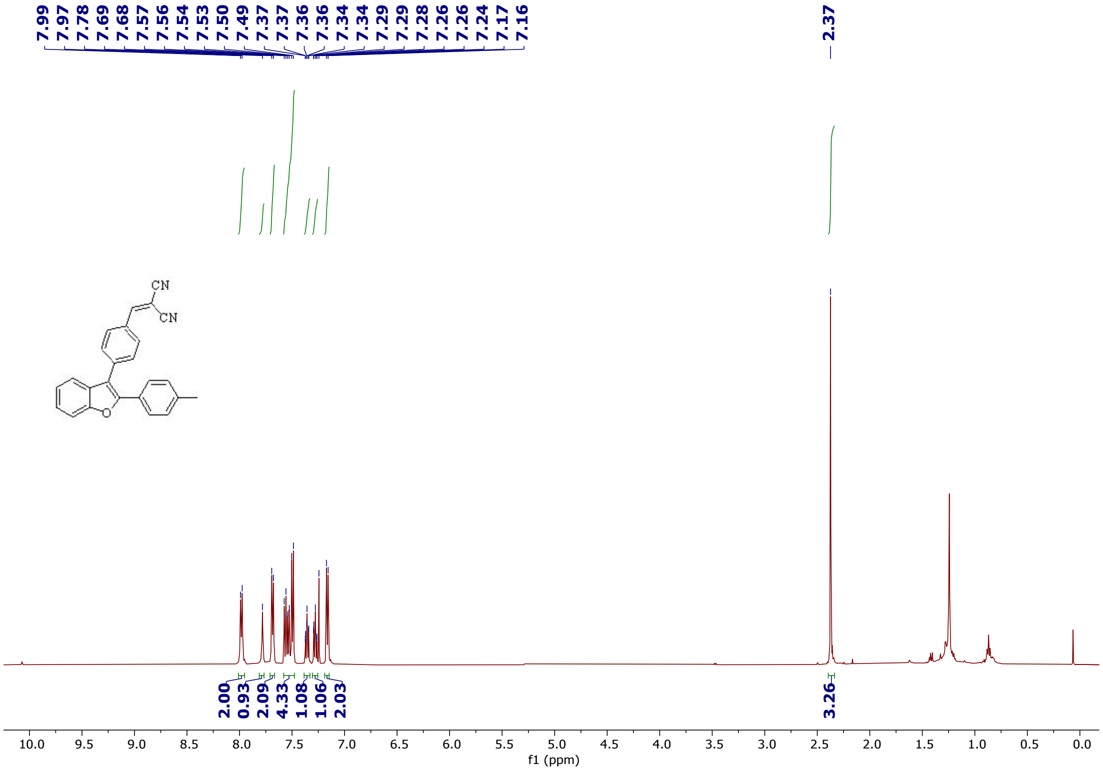

Figure S65: ^{1}H NMR spectrum of 4d

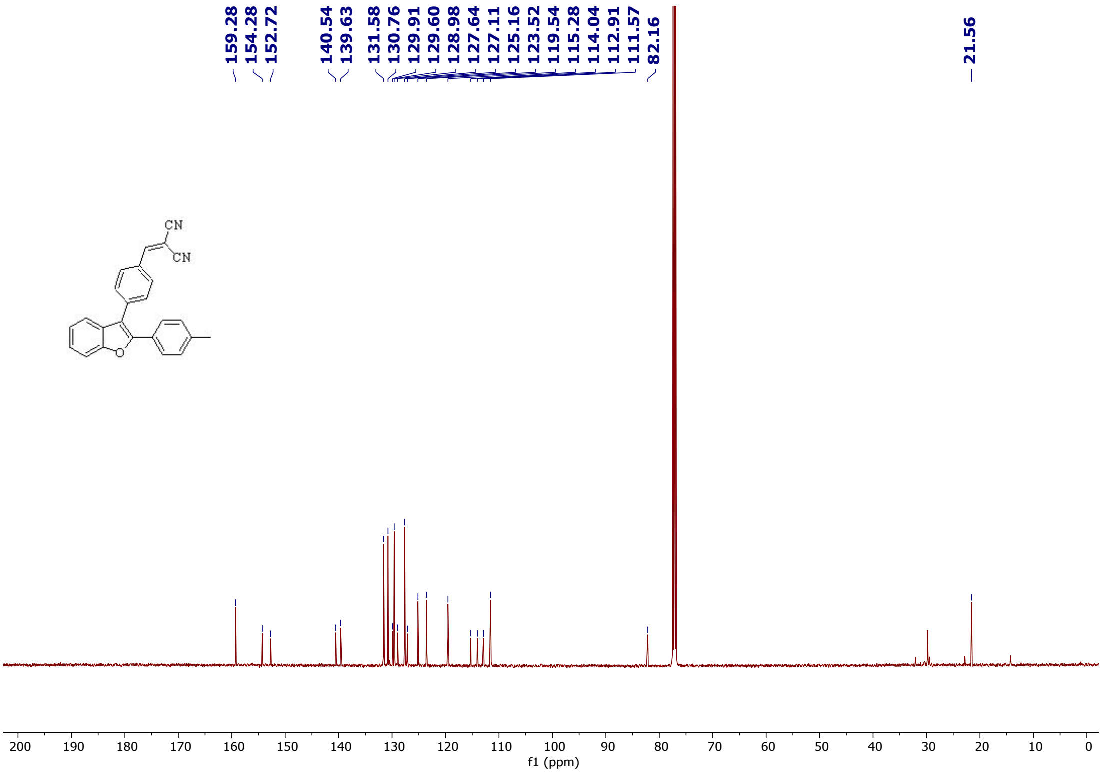

Figure S66: ^{13}C NMR spectrum of 4d

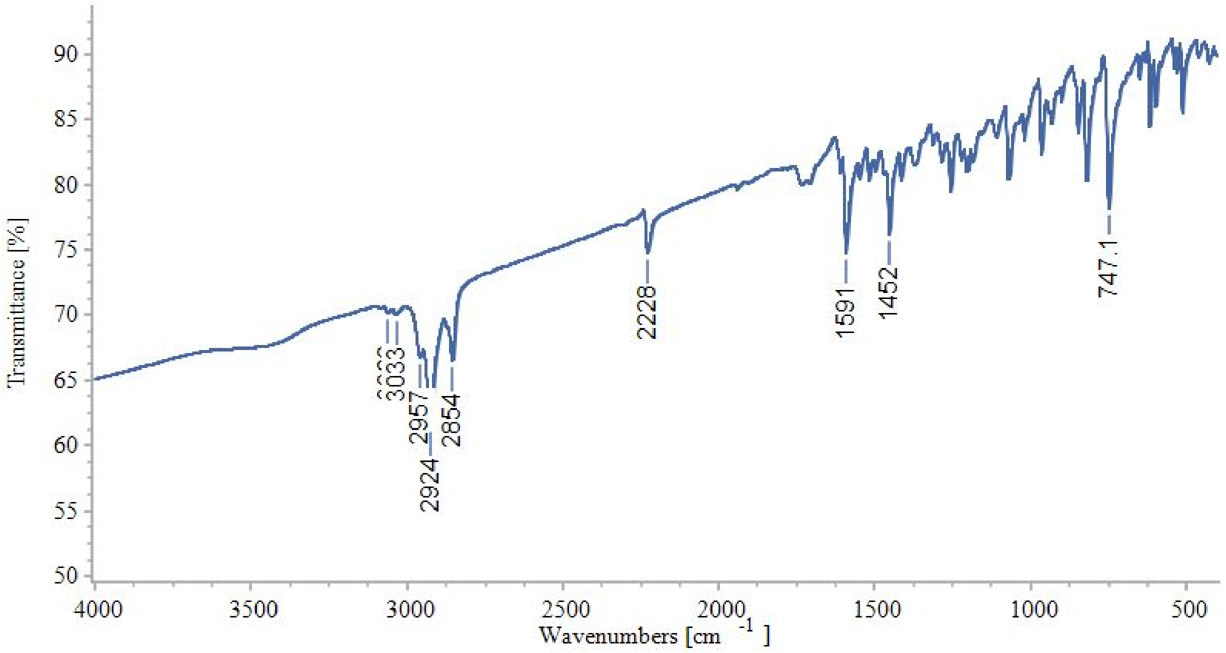

Figure S67: FT-IR spectrum of 4d

H$^+$

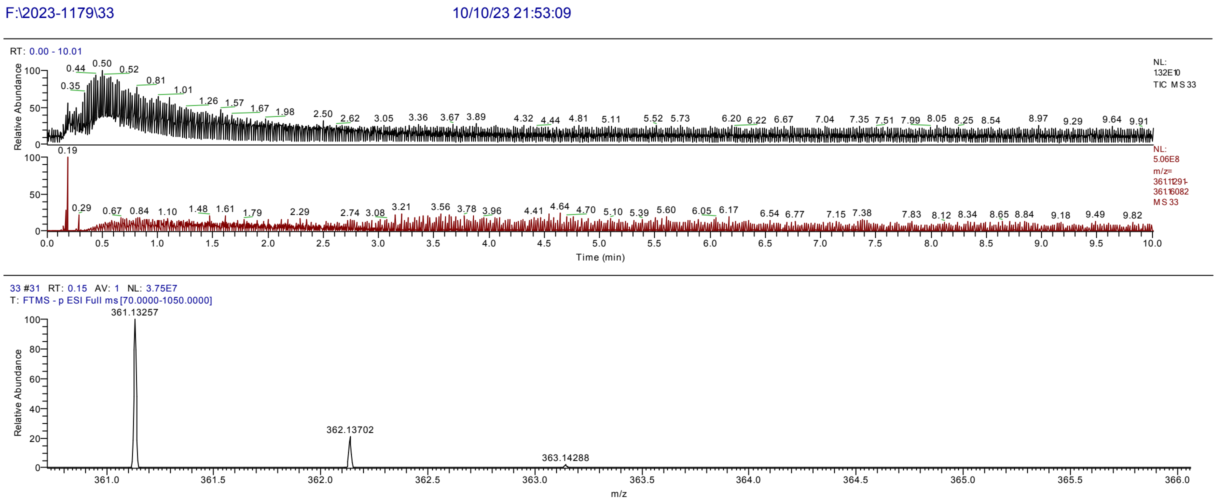

Theoretical

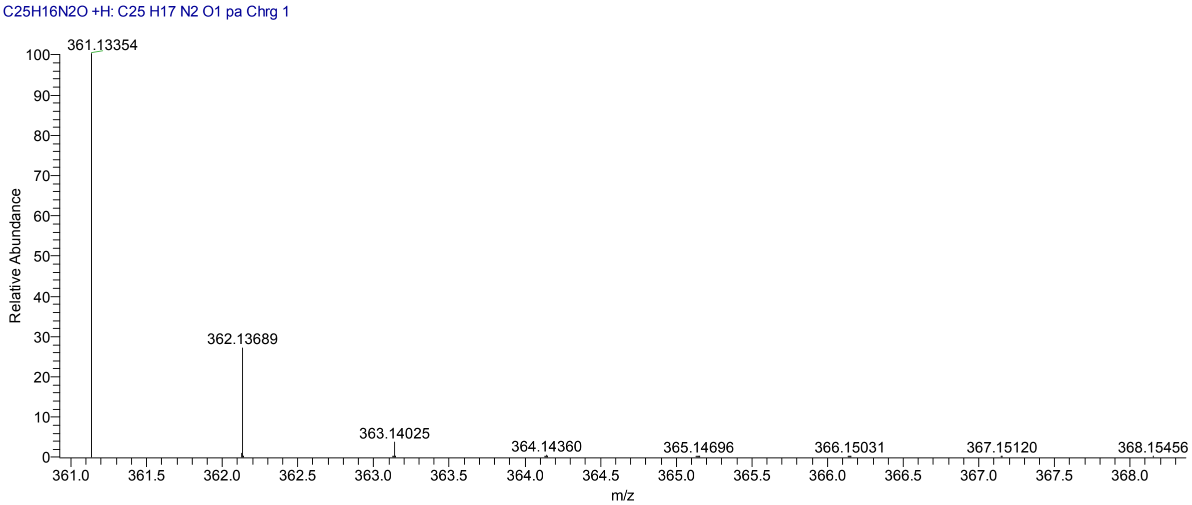

Figure S68: mass spectra of 4d

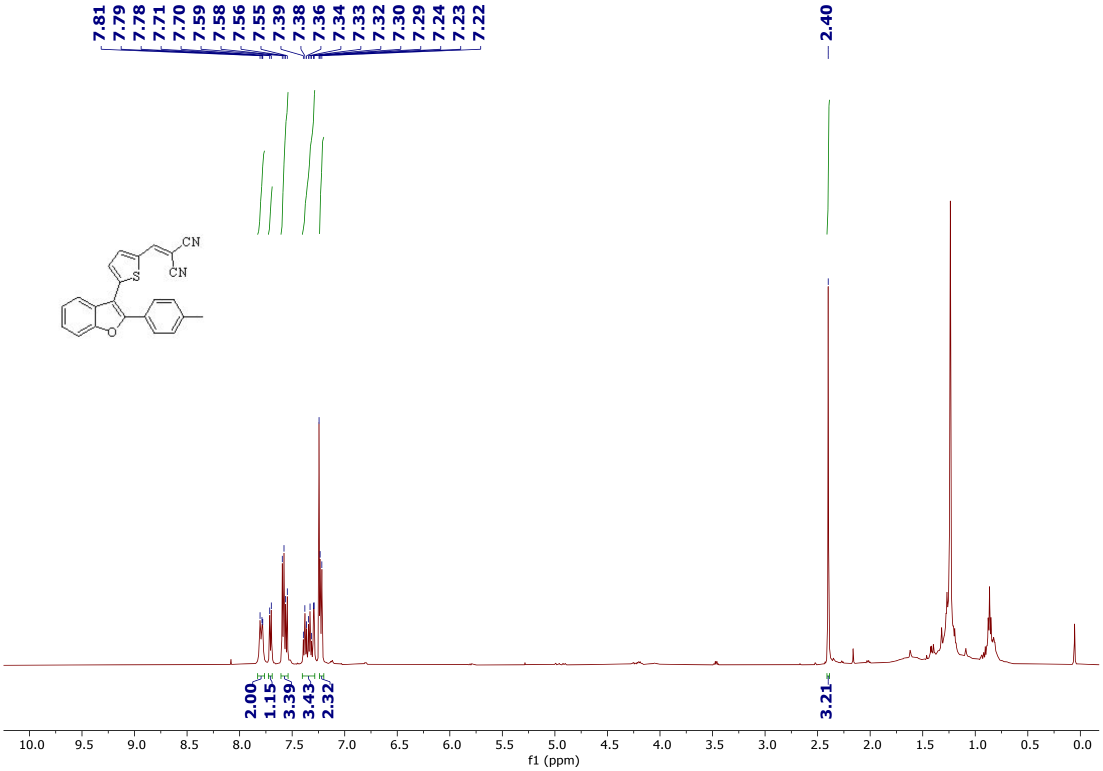

Figure S69: ^{1}H NMR spectrum of 4e

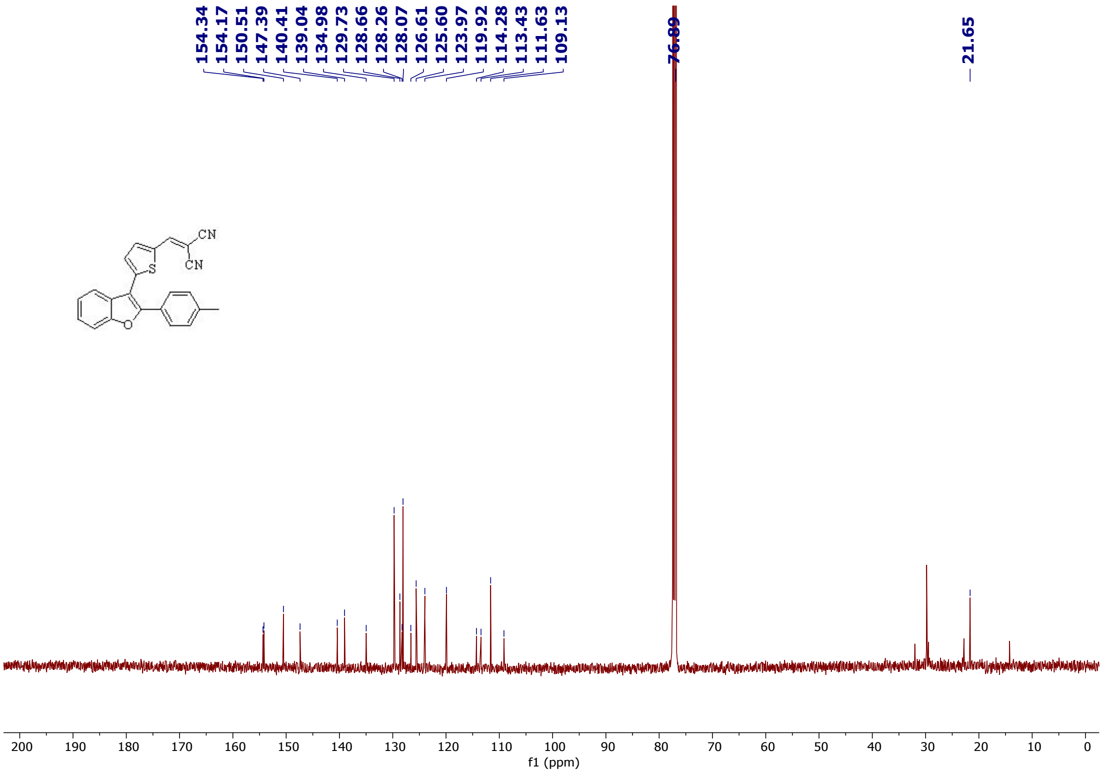

Figure S70: ^{13}C NMR spectrum of 4e

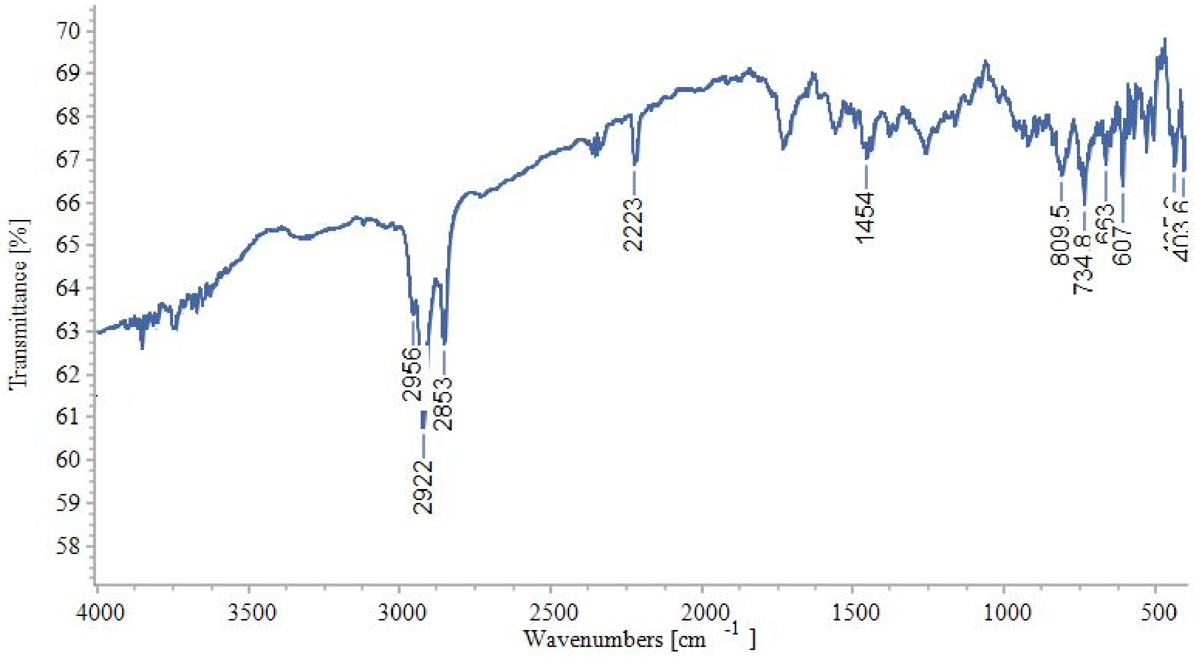

Figure S71: FT-IR spectrum of 4e

H^+

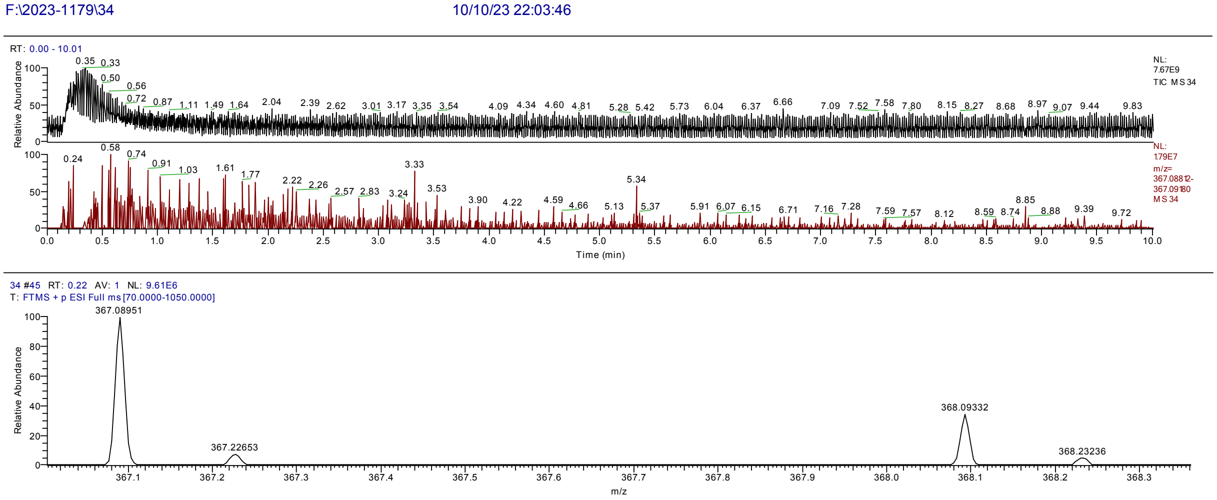

Theoretical

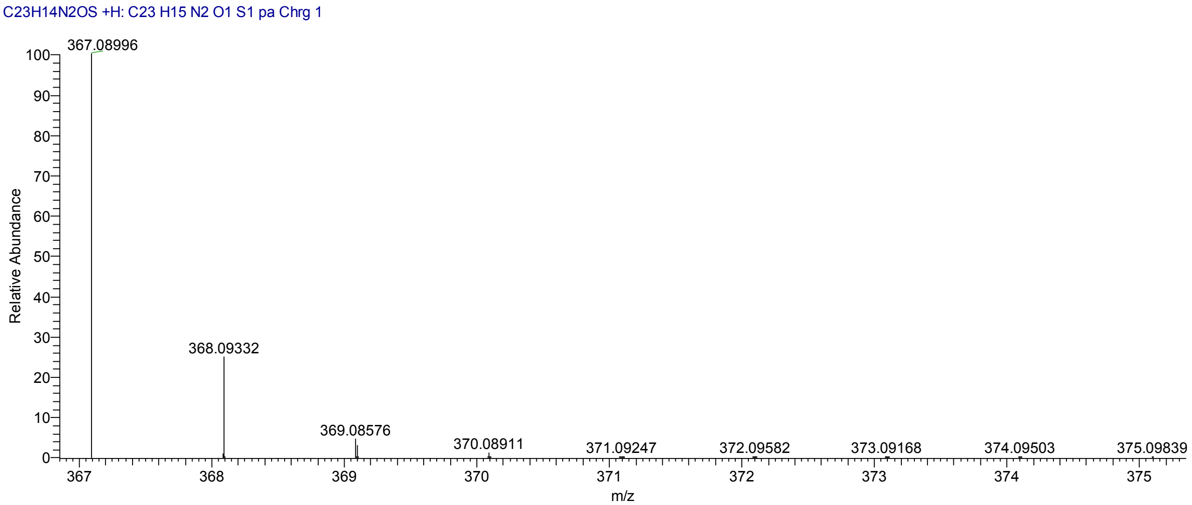

Figure S72: mass spectra of 4e

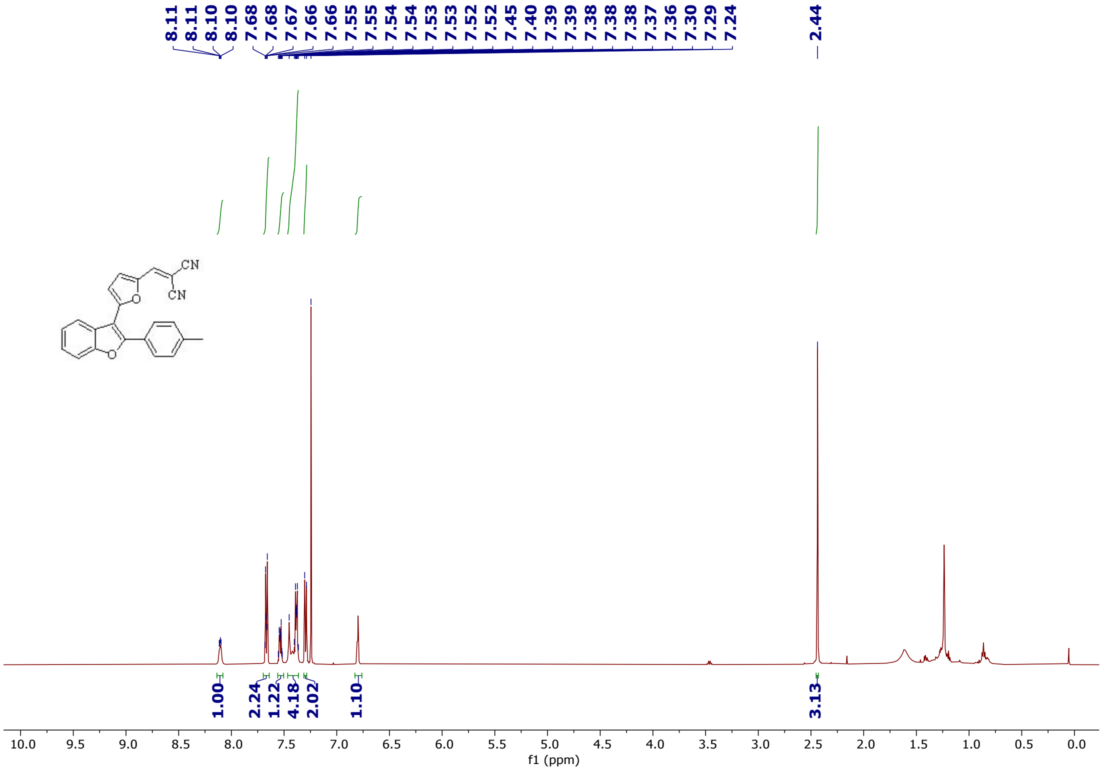

Figure S73: ^{1}H NMR spectrum of 4f

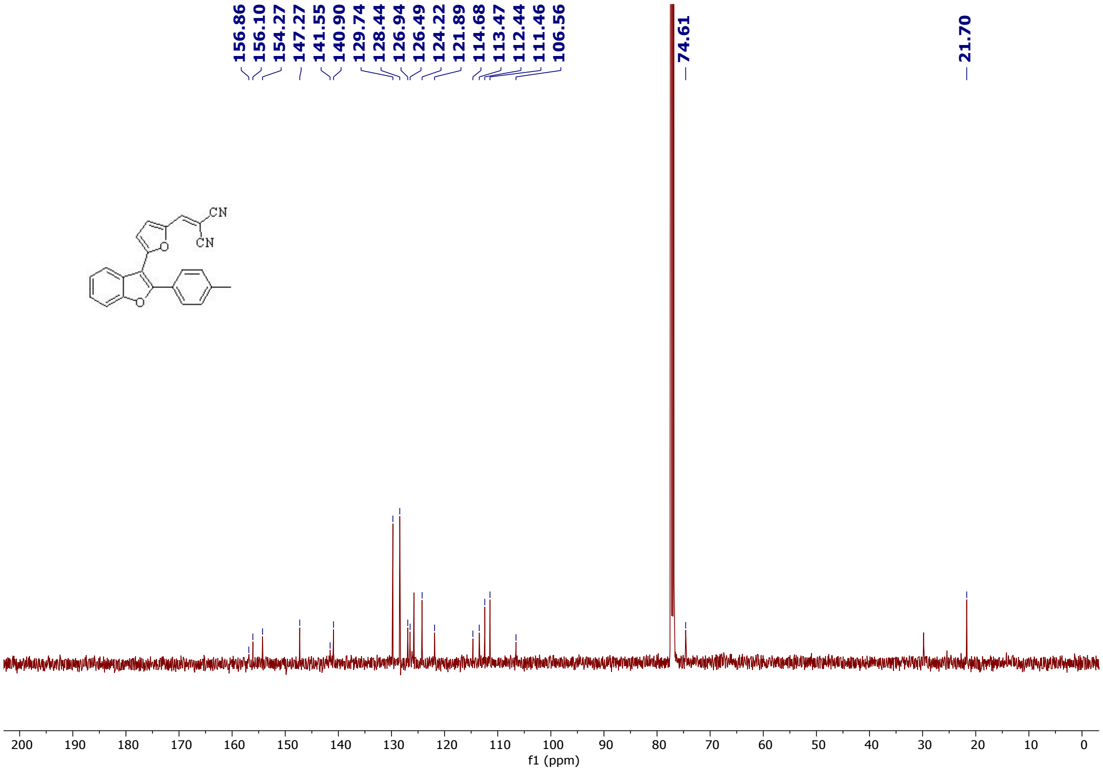

Figure S74: ^{13}C NMR spectrum of 4f

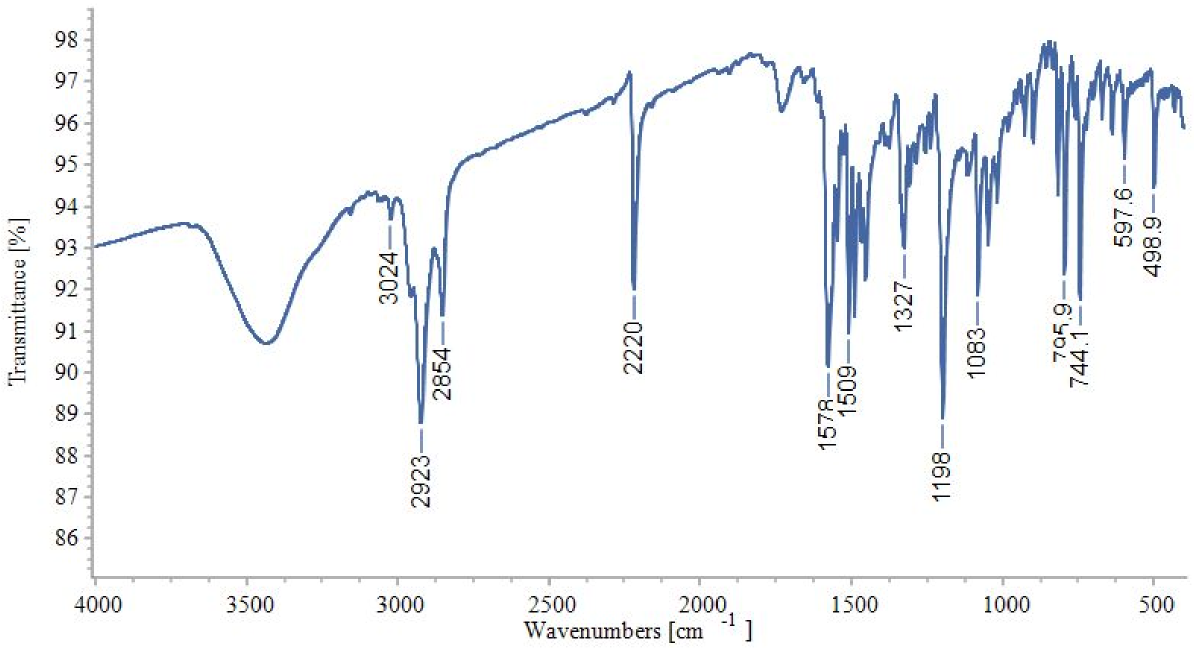

Figure S75: FT-IR spectrum of 4f

H^{+}

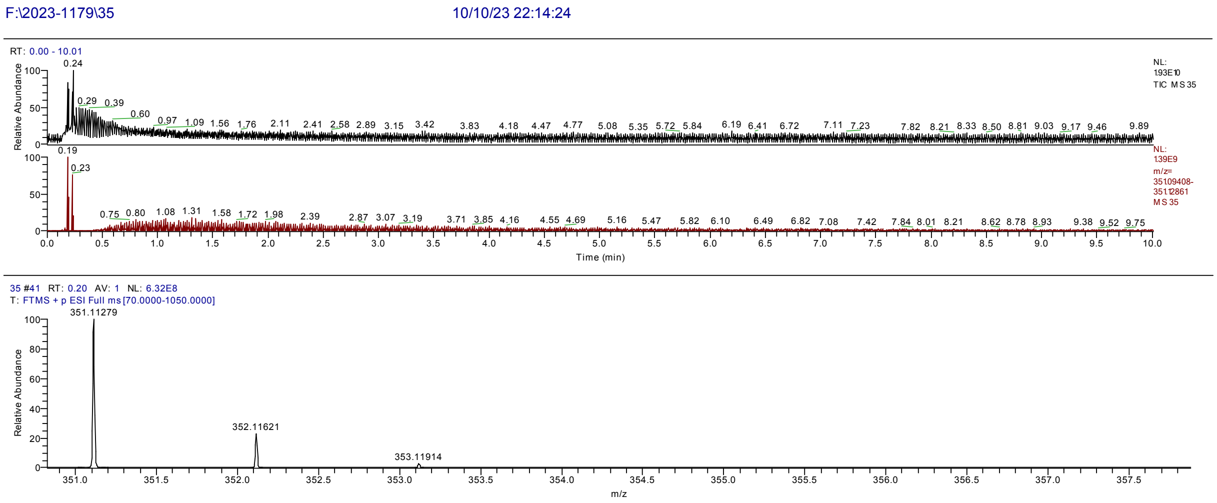

Theoretical

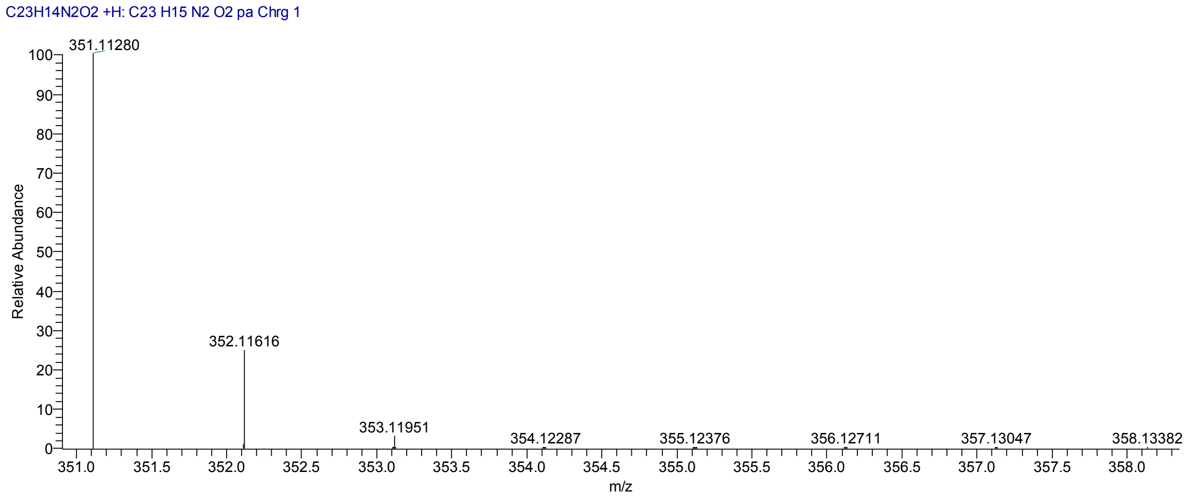

Figure S76: mass spectra of 4f

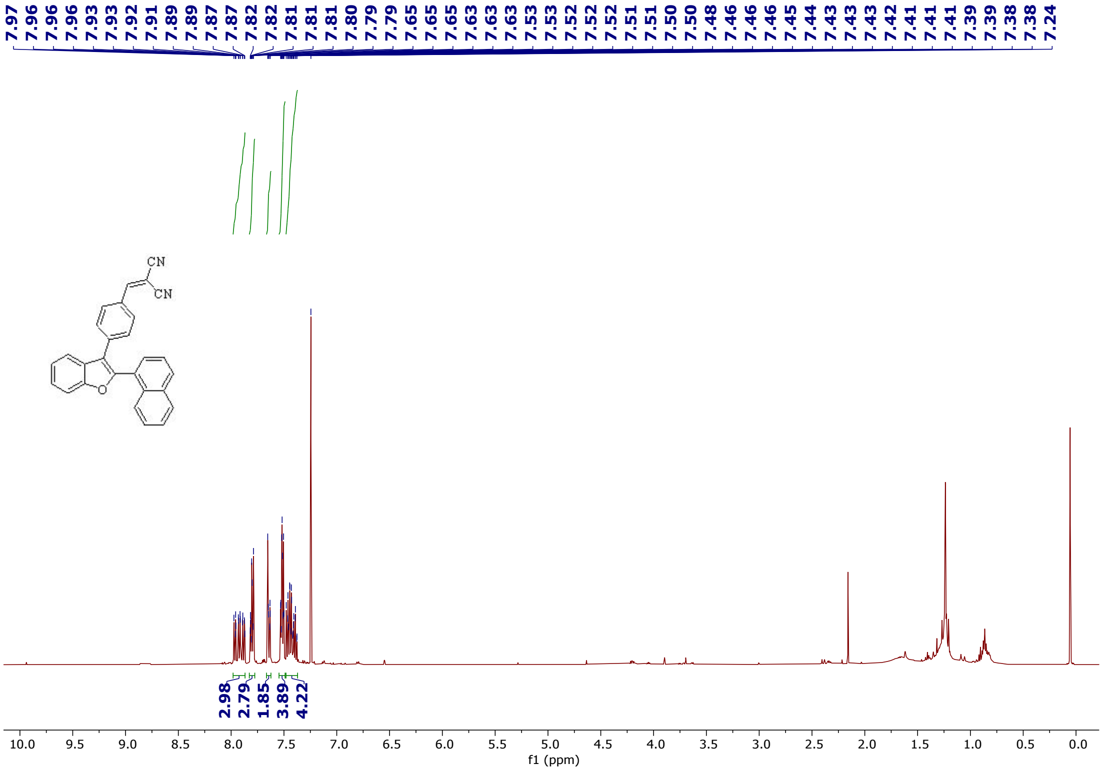

Figure S77: ^{1}H NMR spectrum of 4g

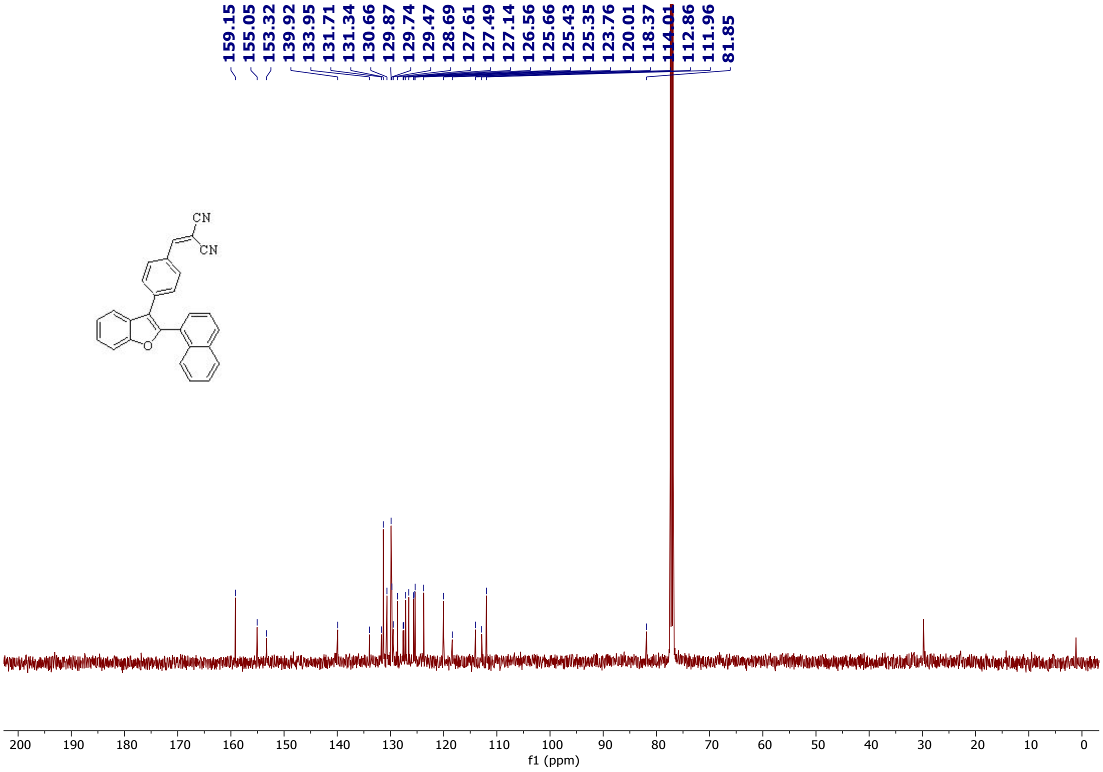

Figure S78: ^{13}C NMR spectrum of 4g

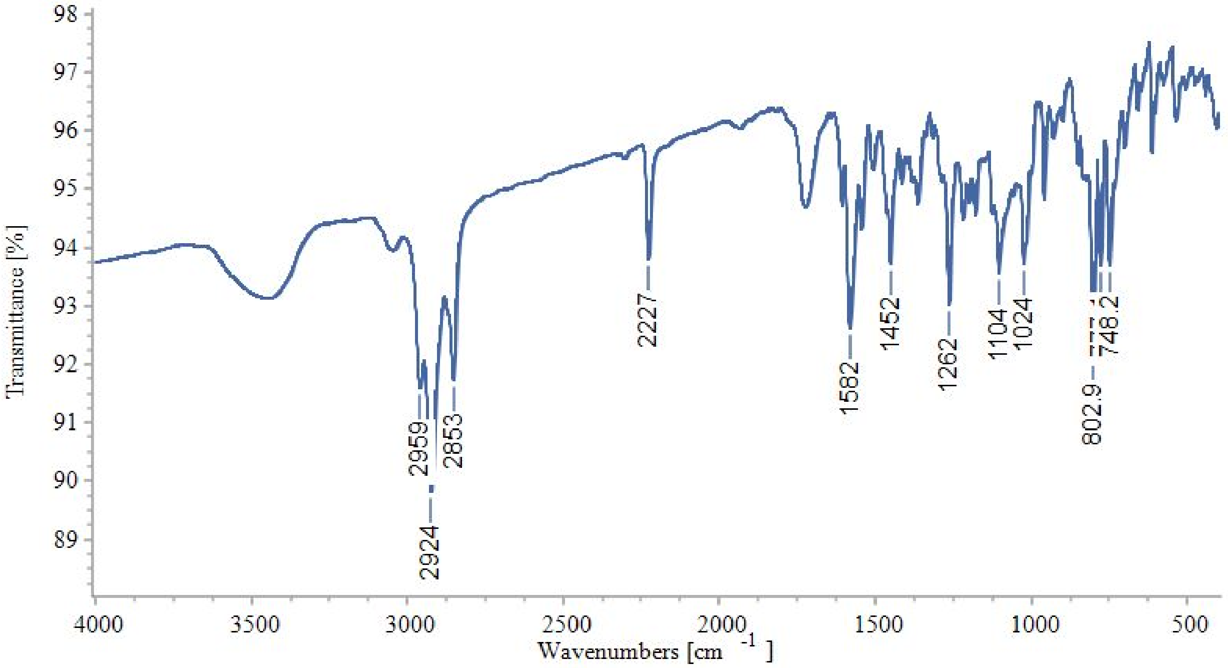

Figure S79: FT-IR spectrum of 4g

H$^+$

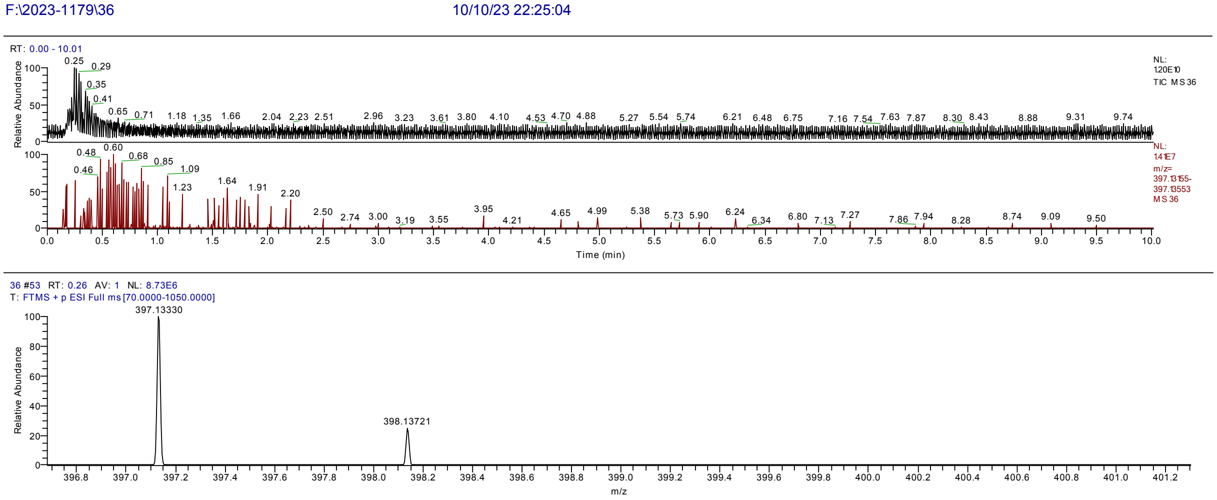

Theoretical

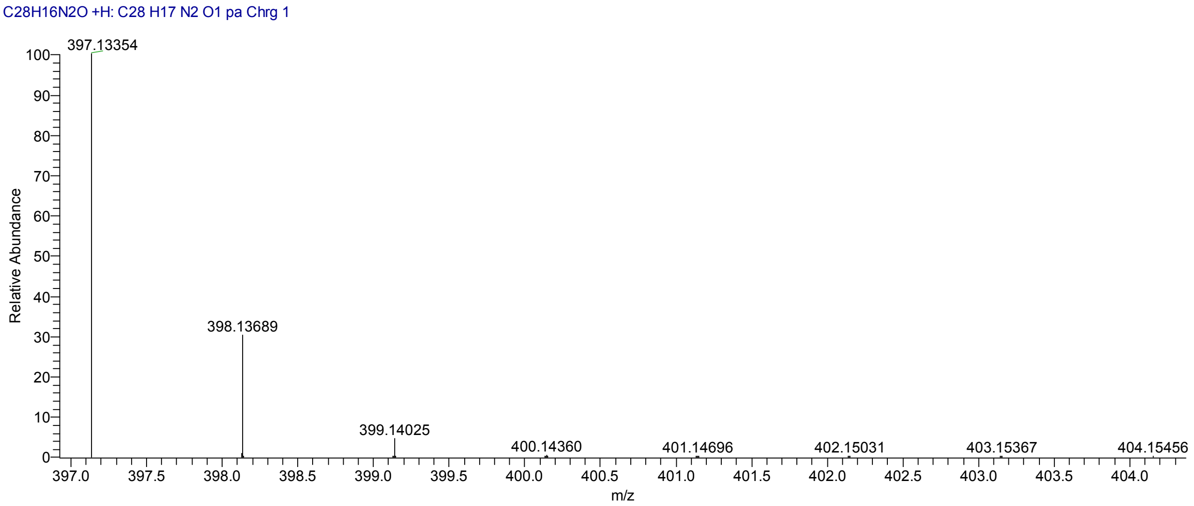

Figure S80: mass spectra of 4g

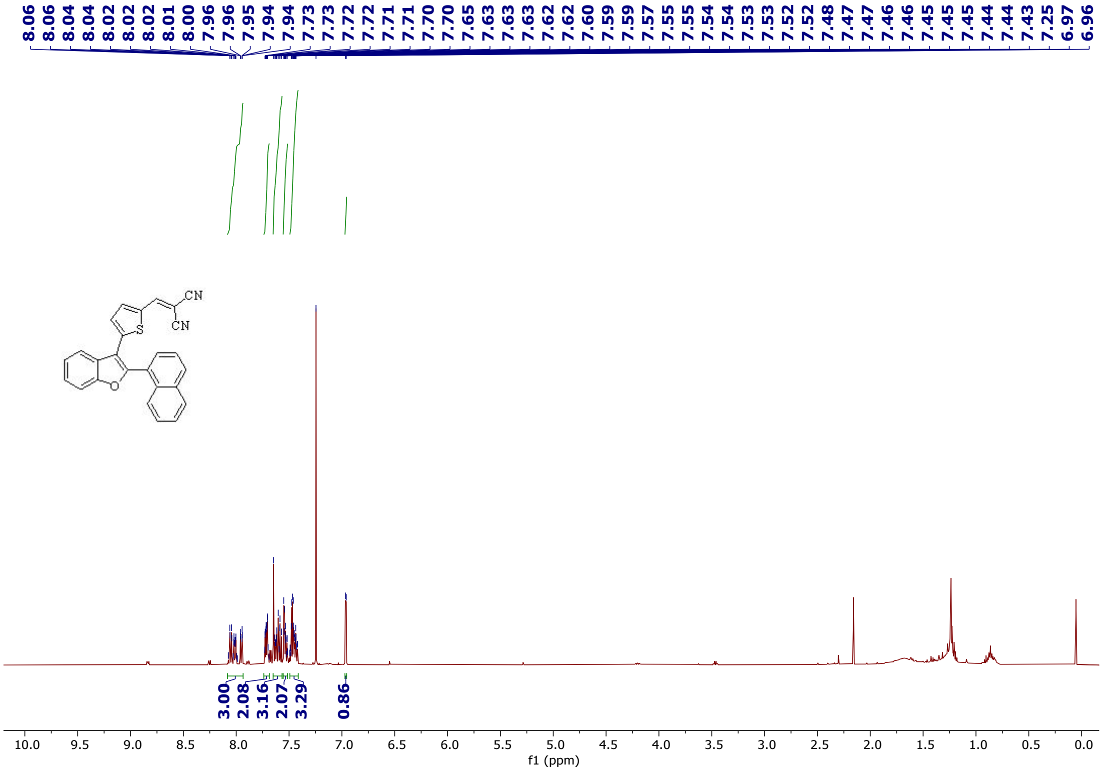

Figure S81: ^{1}H NMR spectrum of 4h

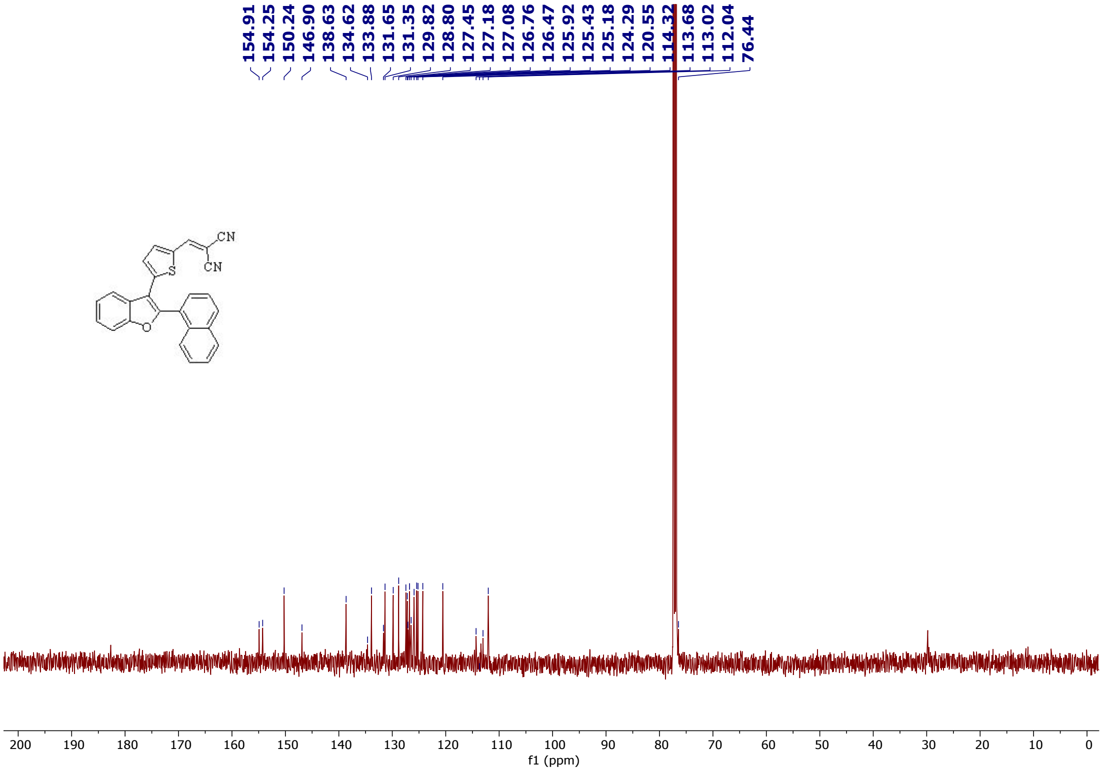

Figure S82: ^{13}C NMR spectrum of 4h

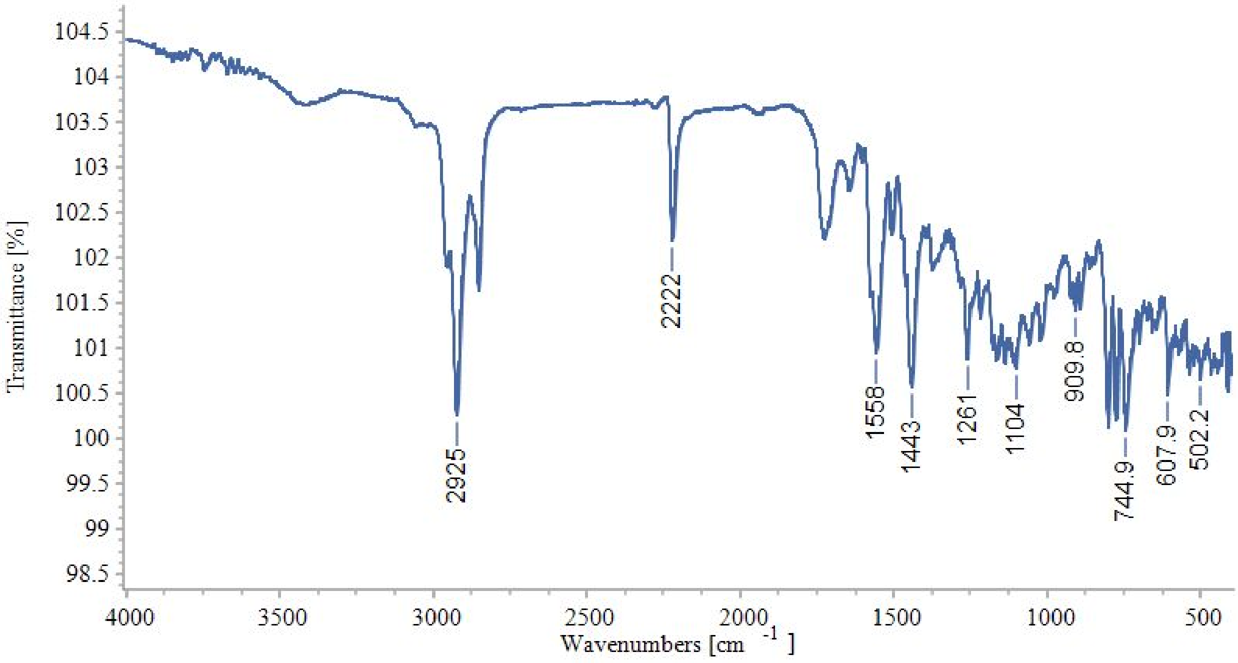

Figure S83: FT-IR spectrum of 4h

H^{+}

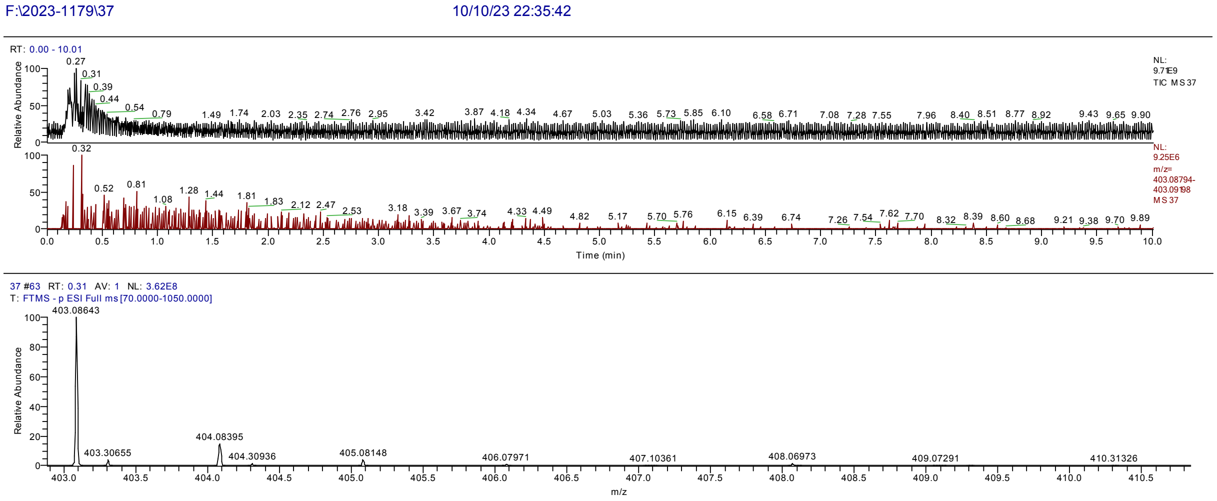

Theoretical

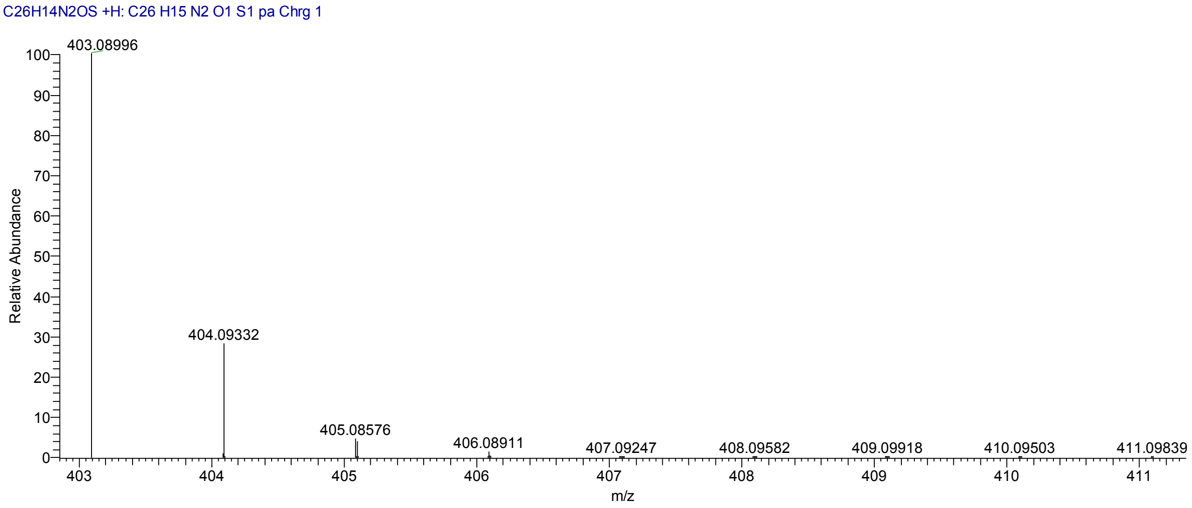

Figure S84: mass spectra of 4h

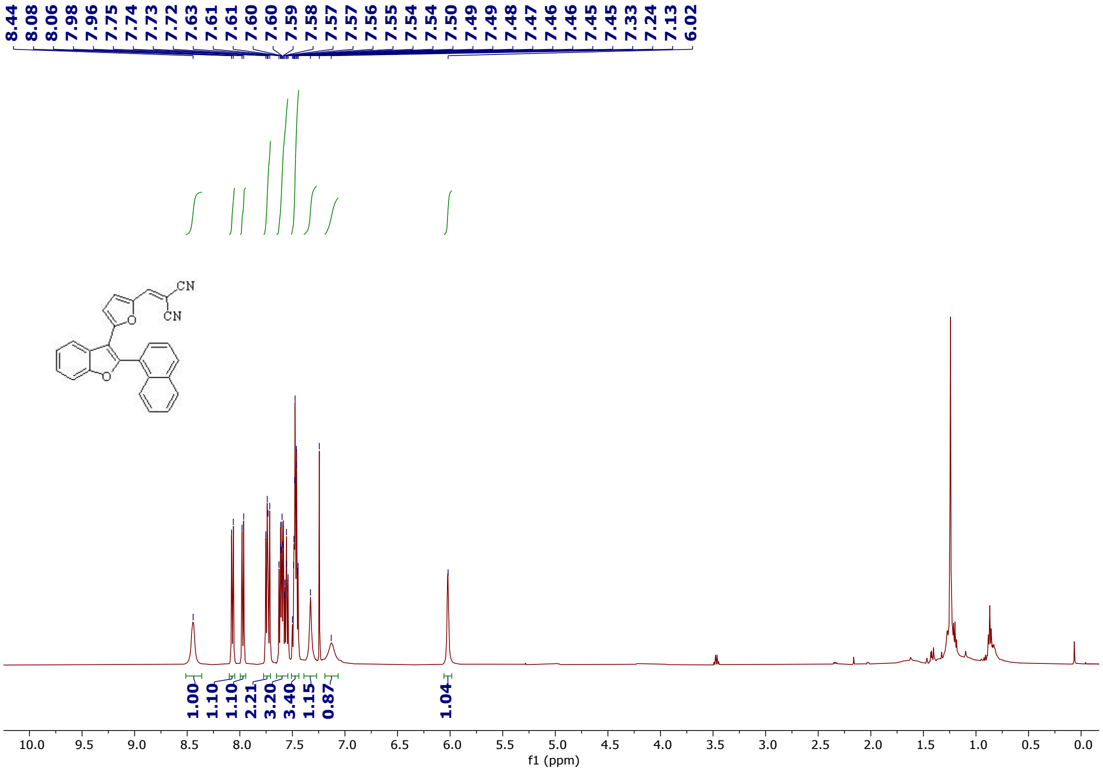

Figure S85: ^{1}H NMR spectrum of 4i

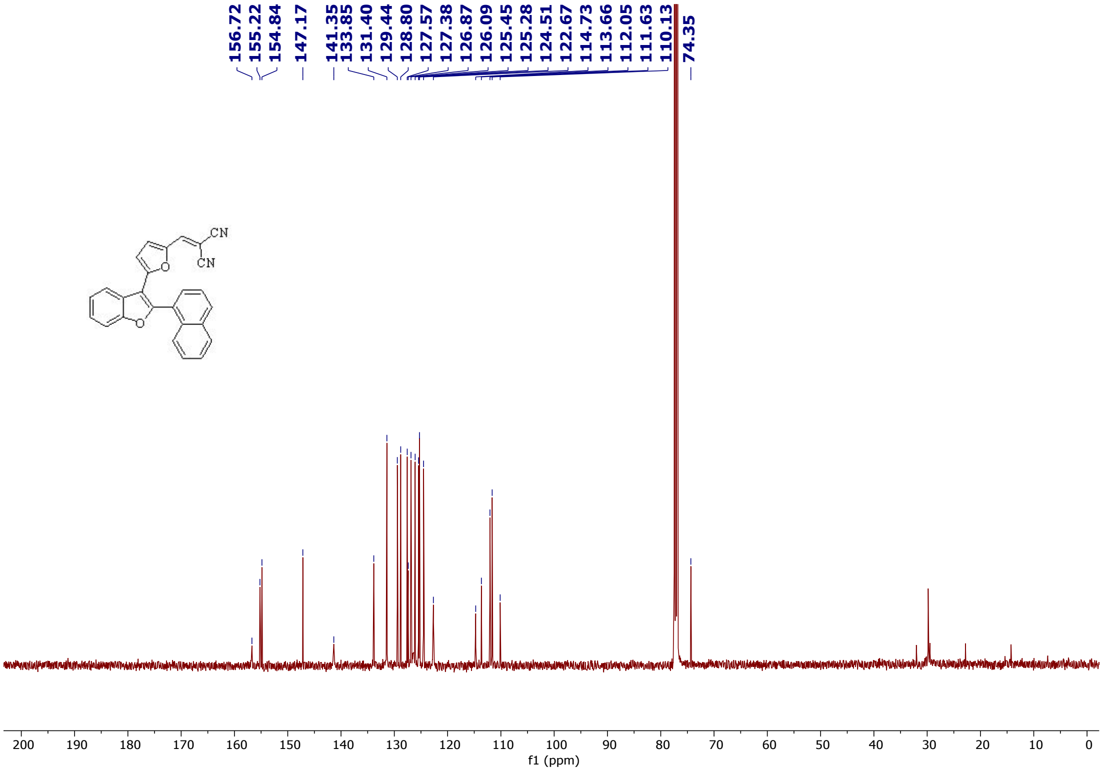

Figure S86: ^{13}C NMR spectrum of 4i

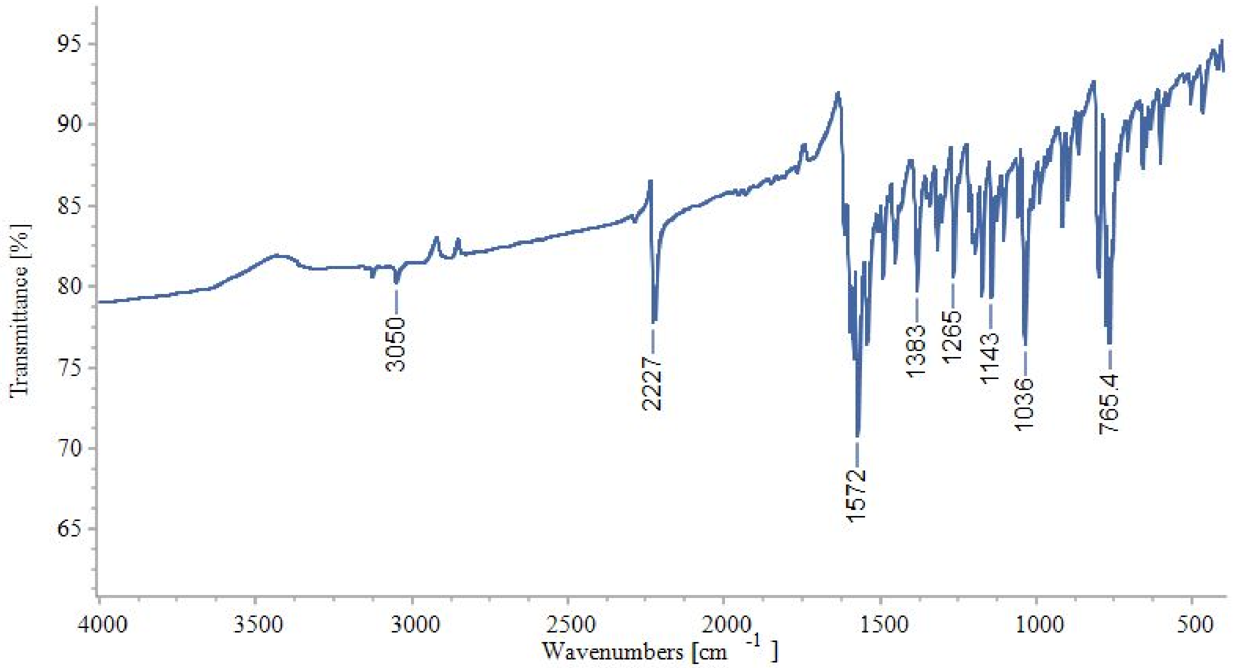

Figure S87: FT-IR spectrum of 4i

H$^+$

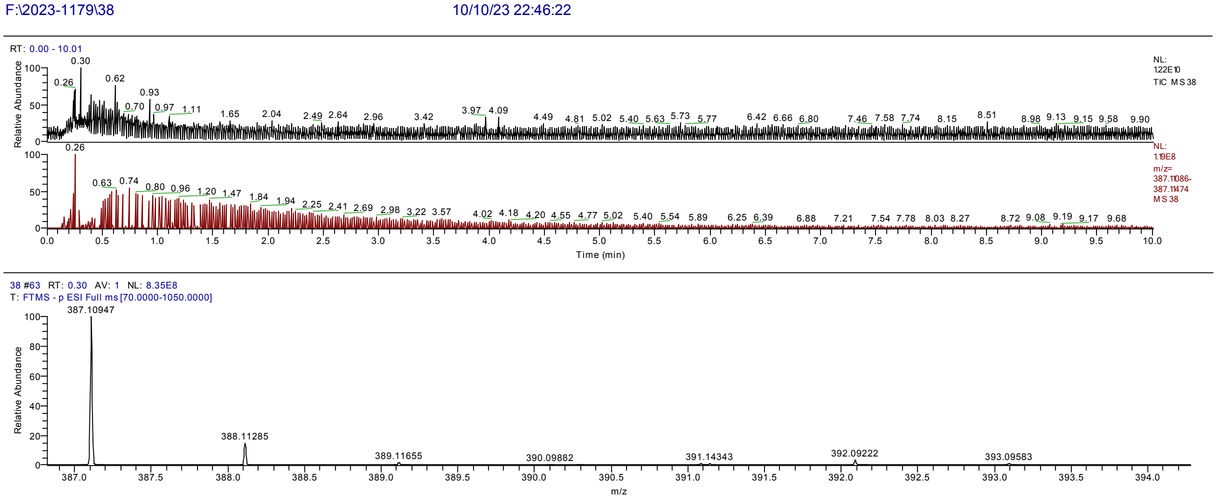

Theoretical

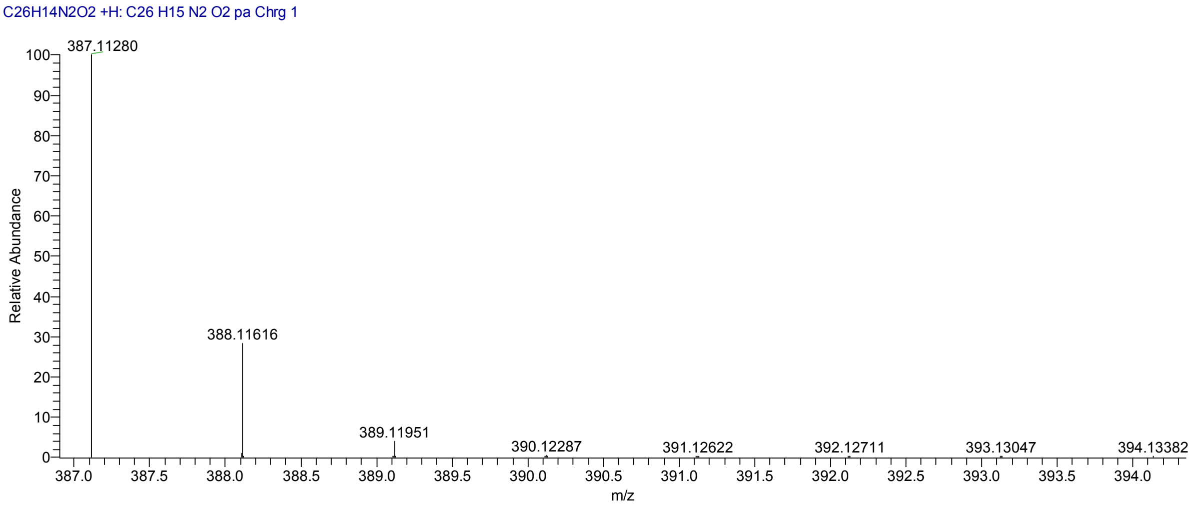

Figure S88: mass spectra of 4i

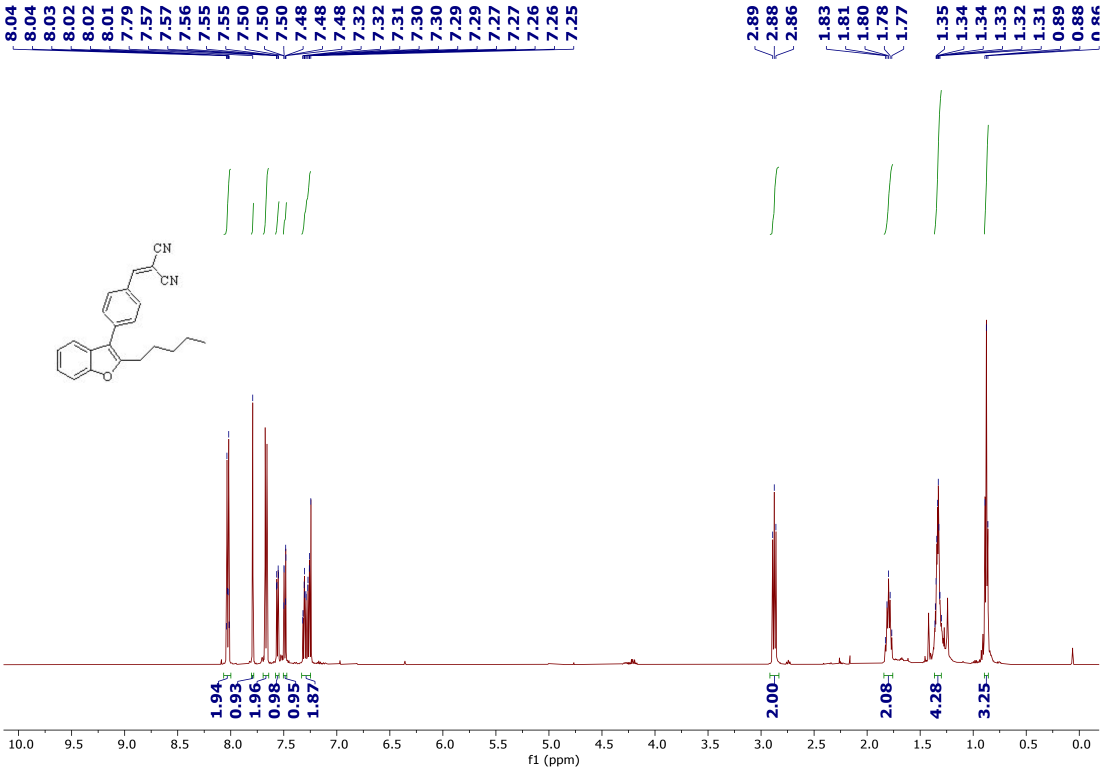

Figure S89: ^{1}H NMR spectrum of 4j

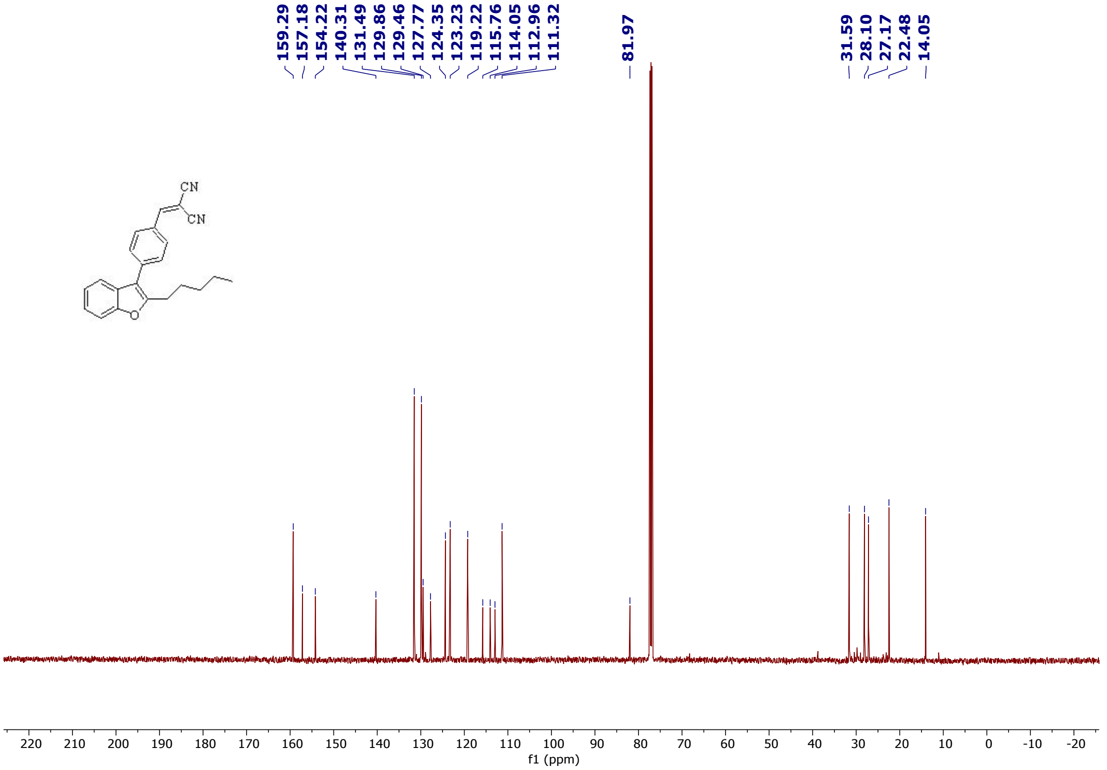

Figure S90: ^{13}C NMR spectrum of 4j

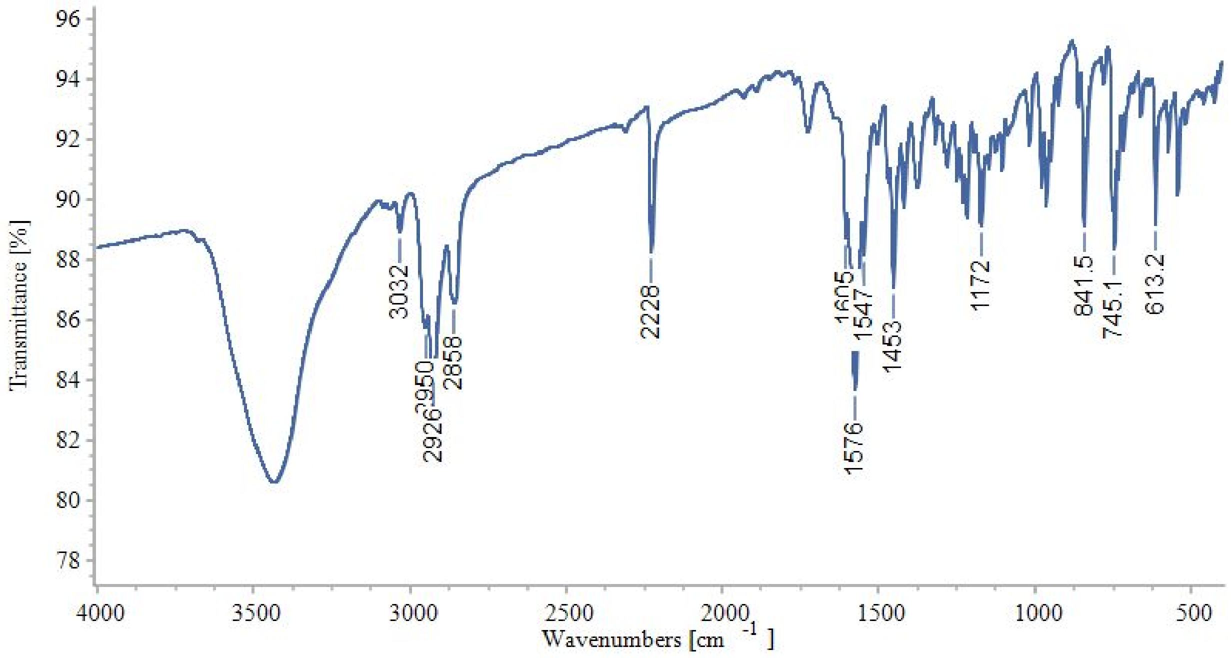

Figure S91: FT-IR spectrum of 4j

H^+

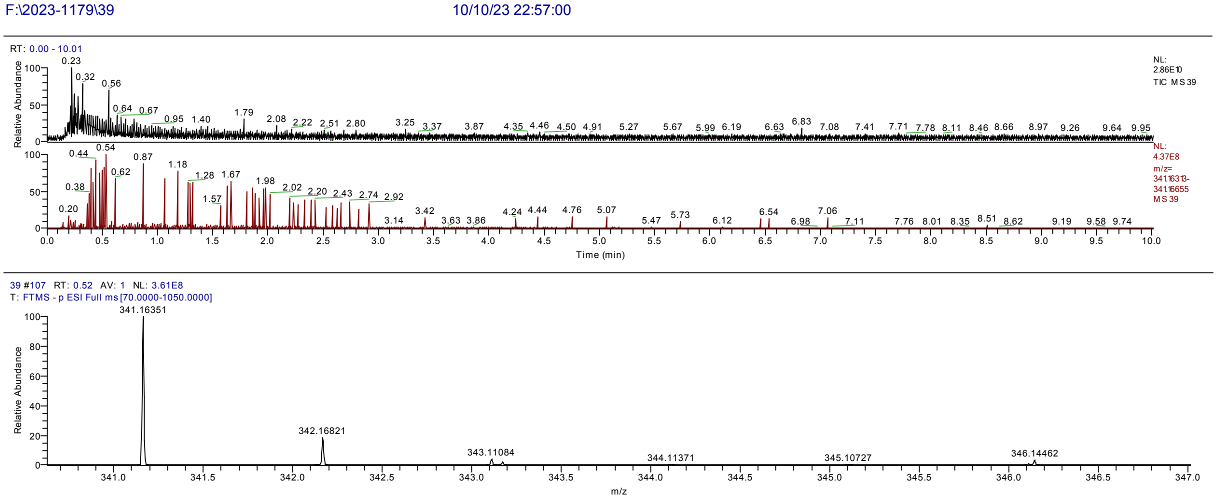

Theoretical

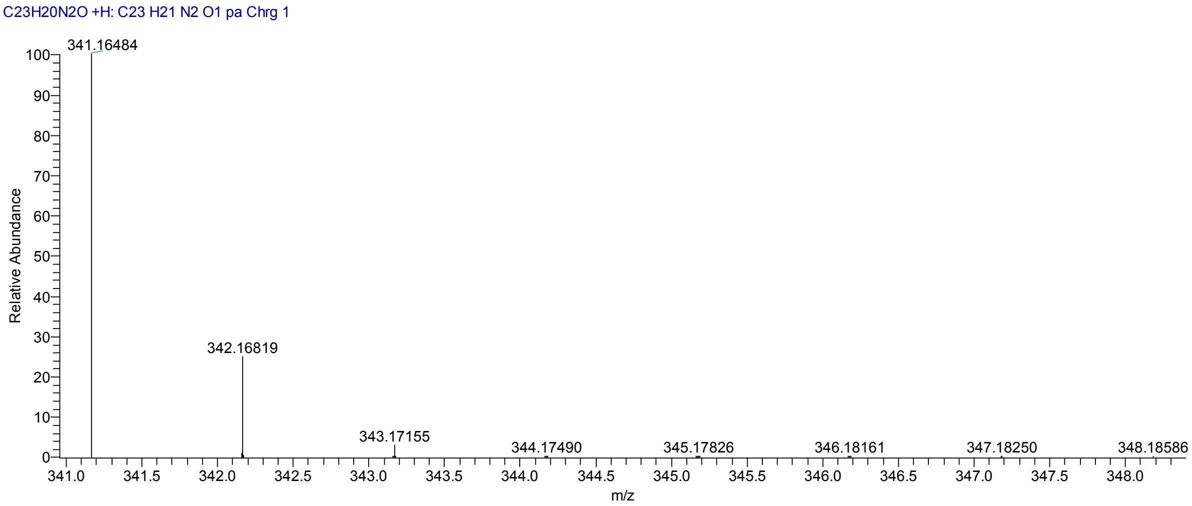

Figure S92: mass spectra of 4j

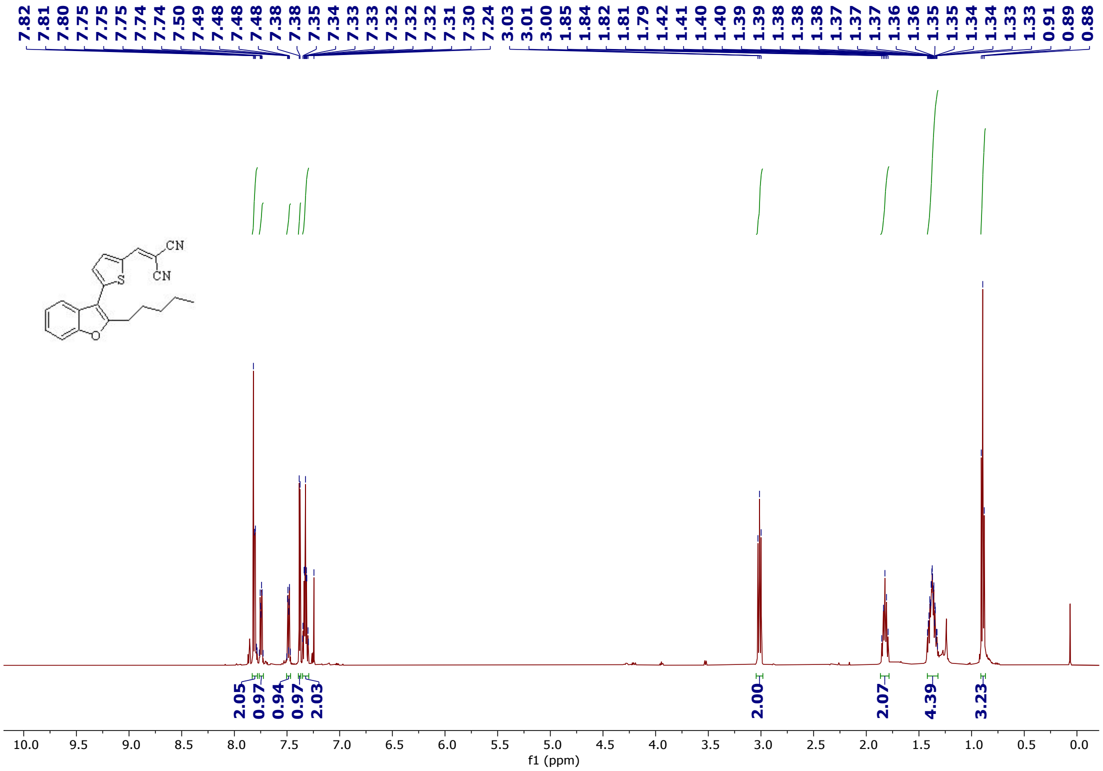

Figure S93: ^{1}H NMR spectrum of 4k

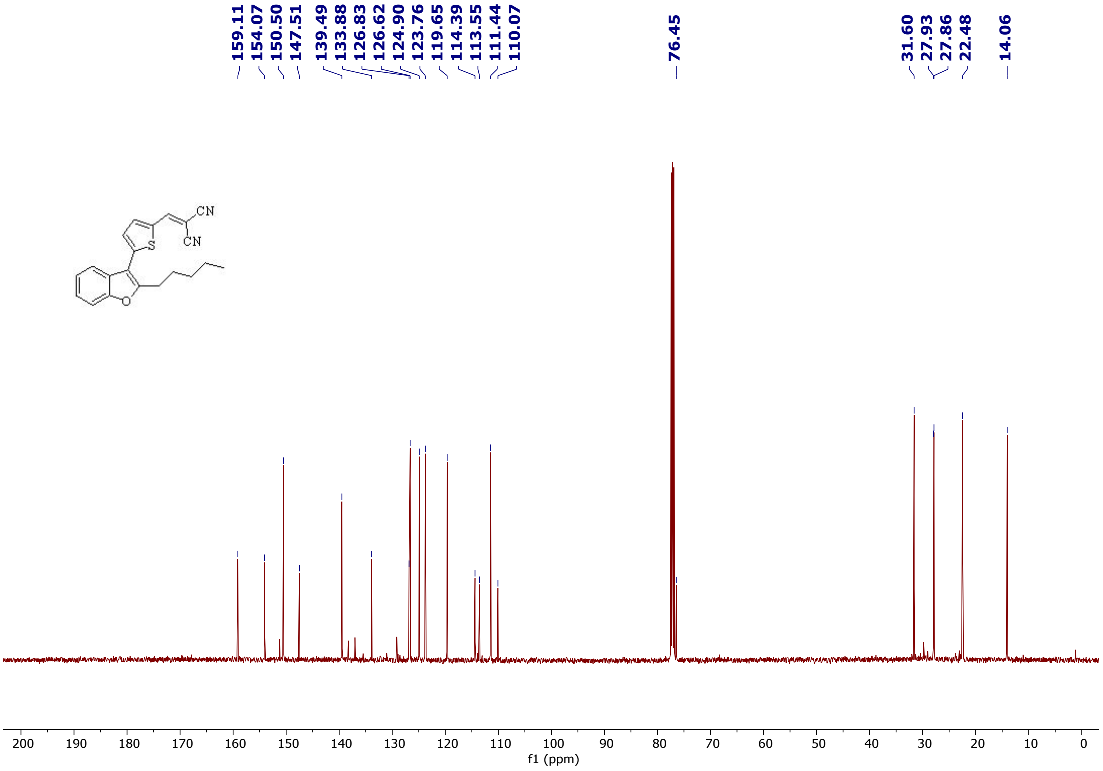

Figure S94: ^{13}C NMR spectrum of 4k

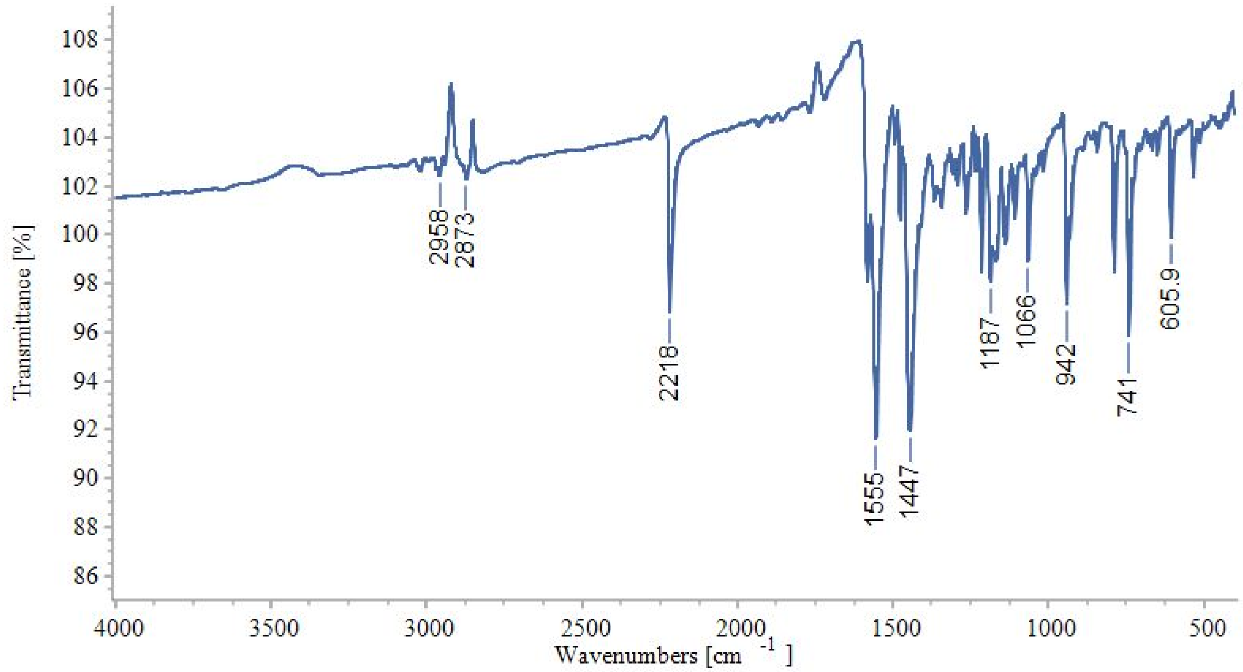

Figure S95: FT-IR spectrum of 4k

H^{+}

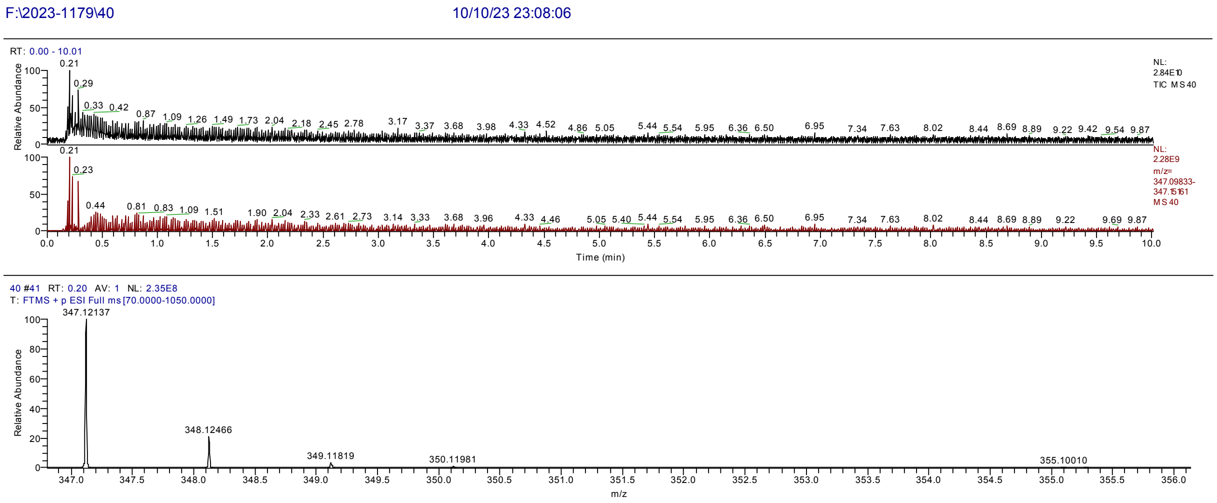

Theoretical

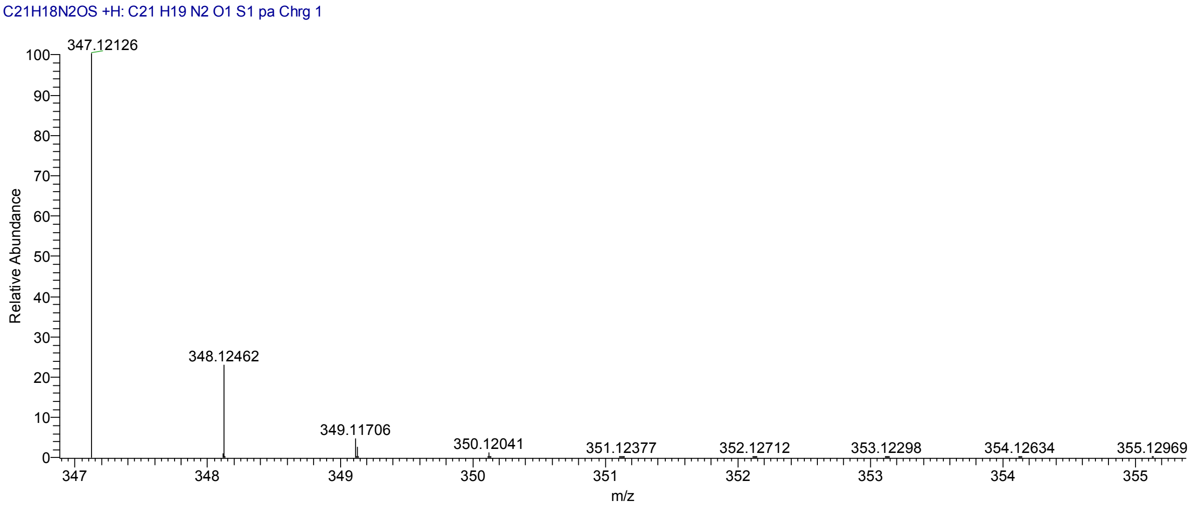

Figure S96: mass spectra of 4k

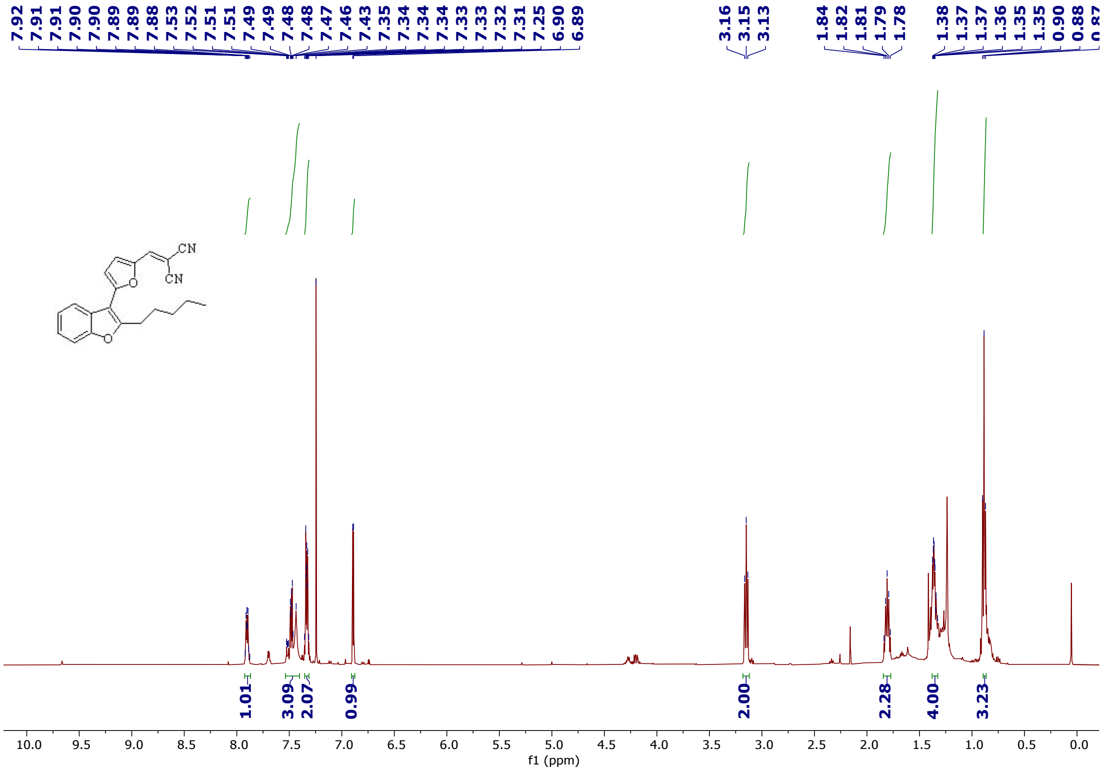

Figure S97: ^{1}H NMR spectrum of 4l

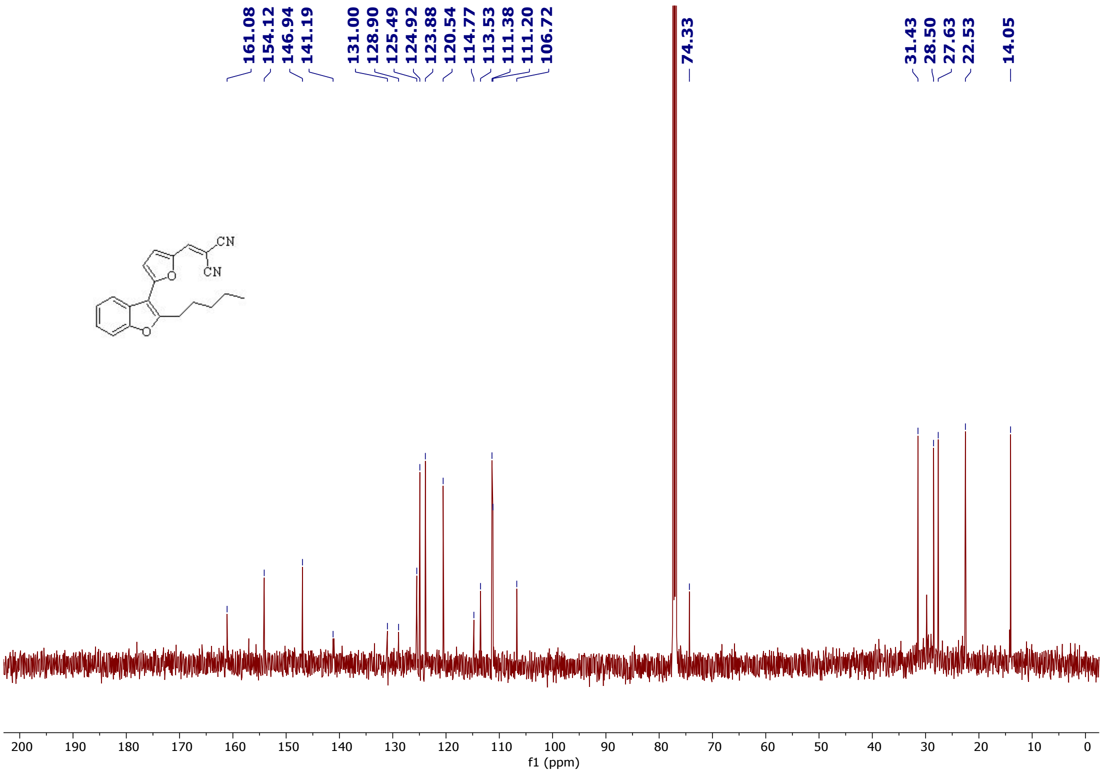

Figure S98: ^{13}C NMR spectrum of 4l

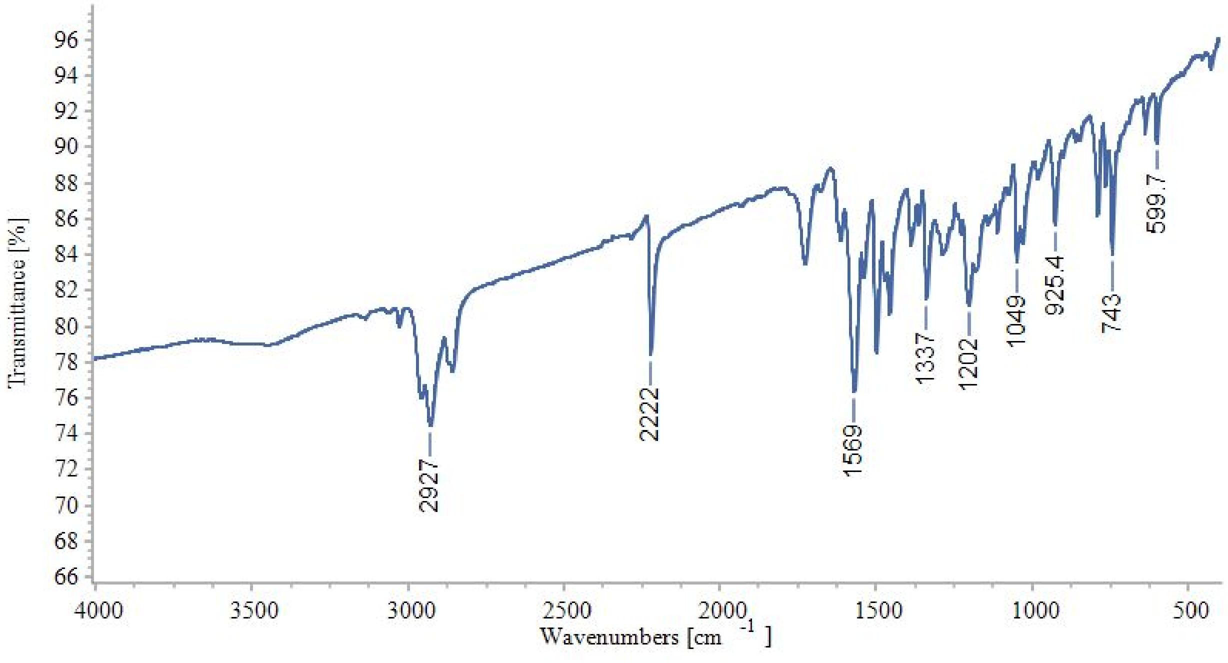

Figure S99: FT-IR spectrum of 4l

H^+

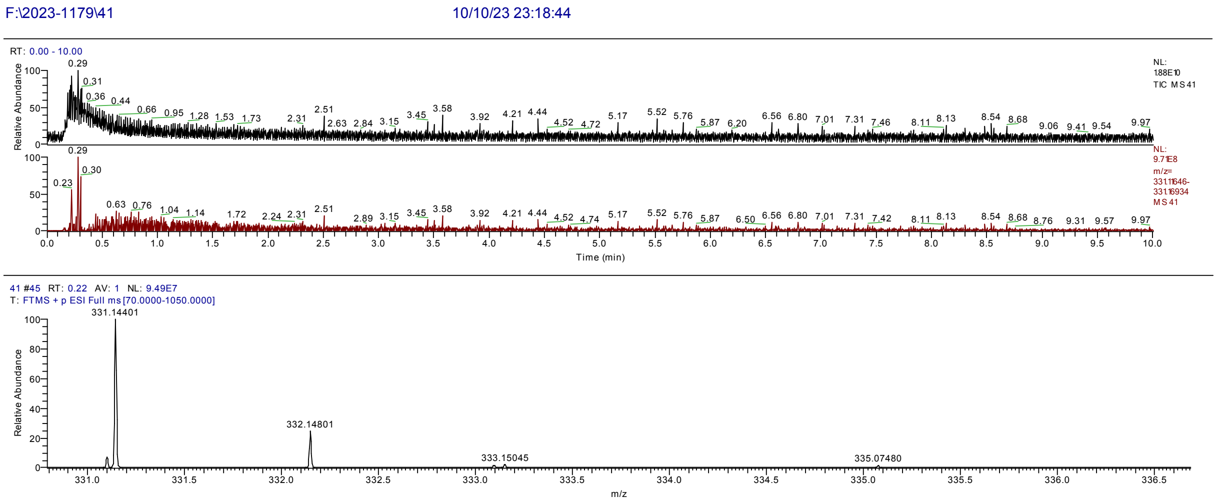

Theoretical

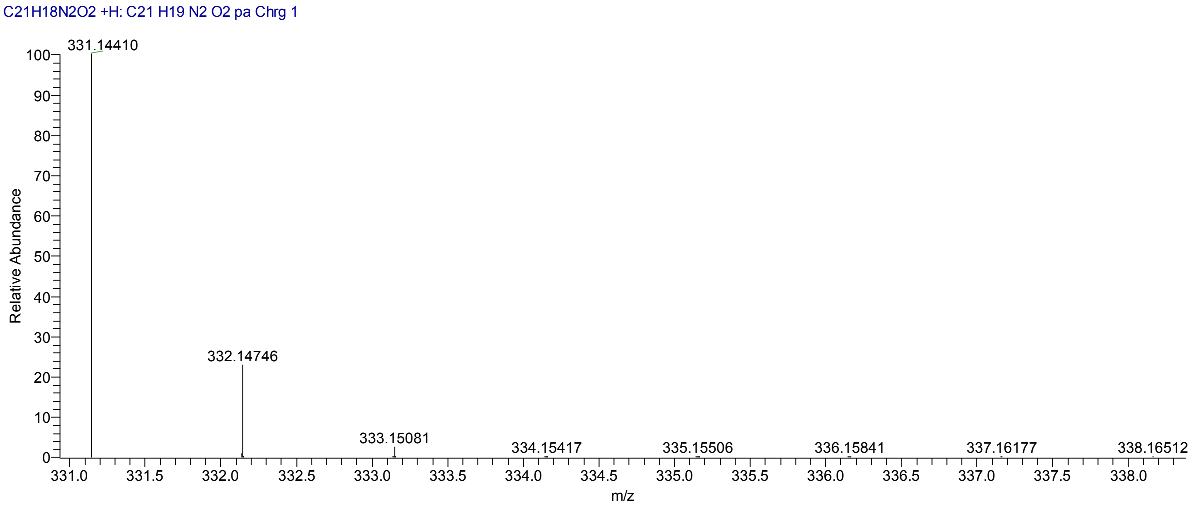

Figure S100: mass spectra of 4l
